# Supplementary material for: An RNA-Binding Protein Secreted by a Bacterial Pathogen Modulates RIG-I Signaling
Source: Cell Host Microbe. 2019 Dec 11;26(6):823–835.e11. doi: 10.1016/j.chom.2019.10.004 (PMC6907008; doi:10.1016/j.chom.2019.10.004)
Supplement: Document S1. Figures S1–S7 and Tables S1 and S2 [file mmc1.pdf]

Cell Host & Microbe, Volume *26*

Supplemental Information

An RNA-Binding Protein Secreted by a Bacterial Pathogen Modulates RIG-I Signaling

Alessandro Pagliuso, To Nam Tham, Eric Allemand, Stevens Robertin, Bruno Dupuy, Quentin Bertrand, Christophe Bécavin, Mikael Koutero, Valérie Najburg, Marie-Anne Nahori, Frédéric Tangy, Fabrizia Stavru, Sergey Bessonov, Andréa Dessen, Christian Muchardt, Alice Lebreton, Anastassia V. Komarova, and Pascale Cossart

SUPPLEMENTAL INFORMATION

Figure S1. Protein sequence alignment of Zea orthologues, related to Figure 1

Multiple alignment of Zea orthologues. Conserved residues are highlighted with colors. The signal peptides at the N-terminus are highlighted in yellow.

Figure S2. RIP-Seq protocol and COG analysis of Zea-interacting RNAs, related to Figure 2

(**A**) Scheme representing the RIP-Seq protocol (see Methods). (**B**) COG functional classes assigned to Zea-bound. Fisher's exact right rank test was run and q-values [-$\log_{10}$(p-value)] were calculated to detect over-represented COG categories.

Figure S3. Extracellular Zea-bound RNAs do not derive from bacterial lysis, related to Figure 1 and Figure 2

(**A**) Confocal microscopy image of *L. monocytogenes* viability by using live/dead staining assay. Live bacteria are stained with SYTO9 (green in the merged channel) and dead bacteria are stained with propidium iodide (PI, red in the merged channel). (**B**) Representative immunoprecipitation (IP) with an anti-Hfq antibody of *L. monocytogenes* bacterial cytosolic extract and culture medium. Hfq runs as an hexamer (60kD). Asterisks show the positions of the IgG heavy. (**C**) RIP-qPCR (n=2) for *LhrA* on RNA isolated from Hfq and control (IgG) immunoprecipitations in the bacterial cytosol and culture medium. The enrichment of *LhrA* was calculated after normalization to the input fraction.; statistical significance determined by two tailed *t*-test between the IgG IP and Hfq IP; †, not detected.

Figure S4. Phage A118, lma-operon, *rli143* and *zea* expression, related to Figure 4

qRT-PCR analysis (n=3) on total RNA extracted from the indicated *L. monocytogenes* strains grown to the exponential phase in MM. Data were normalized to *16S* rRNA level. (**A**) Quantification of selected phage and control genes; values reprents means ± SEM, n=3. (**B**) Quantification of *zea* expression in MM. (**C**) Quantification of selected genes from the lma-monocin locus. Quantification

of (**D**) *rli143* in *L. monocytogenes* and (**E**) *rli143* in *L. innocua*. Data were normalized to *16S* rRNA and level in the *wt* strain. Statistical significance determined by either unpaired ANOVA with multiple testing against *wt* (for A, C and D) or two tailed *t*-test (for B).

Figure S5. Zea protects *rli143* from RNase degradation and regulates the extracellular amount of a subset of *L. monocytogenes* RNA, related to Figure 4

(**A**) *In vitro*-transcribed radiolabeled *rli143* was incubated with either HisZea or GST. RNase I was subsequently added and samples were incubated at 37 ˚C for the indicated time. (**B**) Differential analysis of RNA-Seq quantification of secreted RNAs from *L. monocytogenes wt* and *Δzea*+*zea*$^{+}$ strains (see methods). (**C**) Overlap between the RIP-Seq and the RNA-Seq datasets. A Fisher's exact right rank test shows that the ratio of proportion of RNAs differently expressed and enriched in the RIP-seq is greater than 1 (p = 7.18x10^{-5}). The list highlights the genes found in both datasets. (**D**) qRT-PCR analysis on total intracellular RNA extracted from *L. monocytogenes wt* and *Δzea*+*zea*$^{+}$ for the indicated phage genes. Data were normalized to *16S* rRNA. Statistical significance determined by two tailed *t*-test.

Figure S6. RIG-I binds the *L. monocytogenes* A118 phage RNA during infection, related to Figure 6

Interaction circo plots of *L. monocytogenes* RNAs specifically bound to RIG-I, MDA5 and LGP2 during *L. monocytogenes* infection. A line from the schematized RIG-I to a genome locus indicates a preferential binding to a transcript compared to the negative control mCherry. The asterisks highlight the transcripts that are also enriched in the Zea RIPseq dataset.

Figure S7. Zea localizes both to the host cell cytoplasm and nucleus, related to Figure 6

(A) IP analysis with an anti-Flag antibody of cytosolic and nuclear fractions from LoVo cells infected with *wt* and *zea*$_{Flag}$ bacteria. (**B**) Confocal images of LoVo cells transfected with Flag-tagged Zea,

fixed and processed for immunofluorescence by using an anti-Flag antibody (red); nuclei are stained with DAPI. Scale bar, 10 μm.

Figure S1

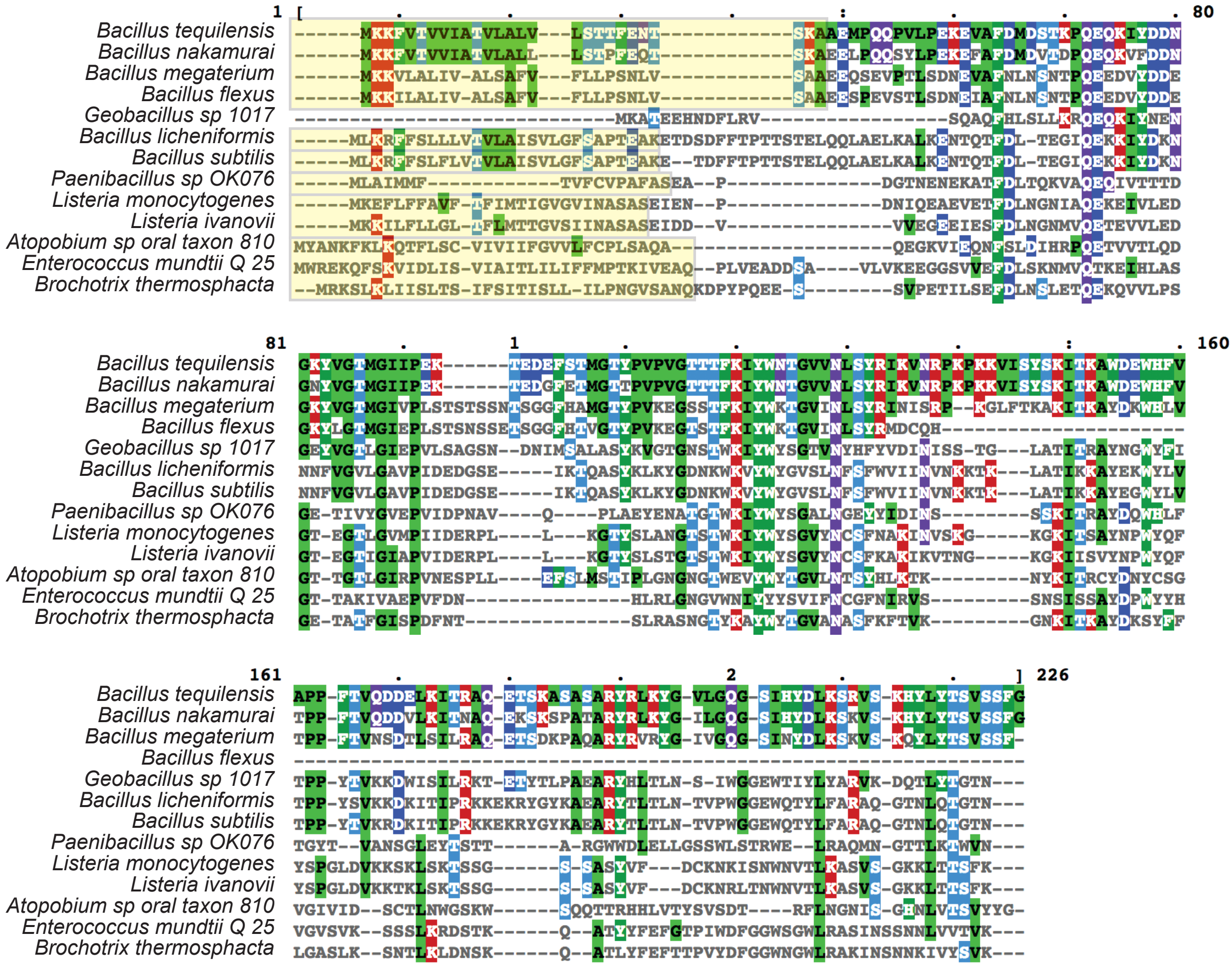
1
80
81
160
161
226
Bacillus tequilensis
Bacillus nakamurai
Bacillus megaterium
Bacillus flexus
Geobacillus sp 1017
Bacillus licheniformis
Bacillus subtilis
Paenibacillus sp OK076
Listeria monocytogenes
Listeria ivanovii
Atopobium sp oral taxon 810
Enterococcus mundtii Q 25
Brochotrix thermosphacta

Figure S2

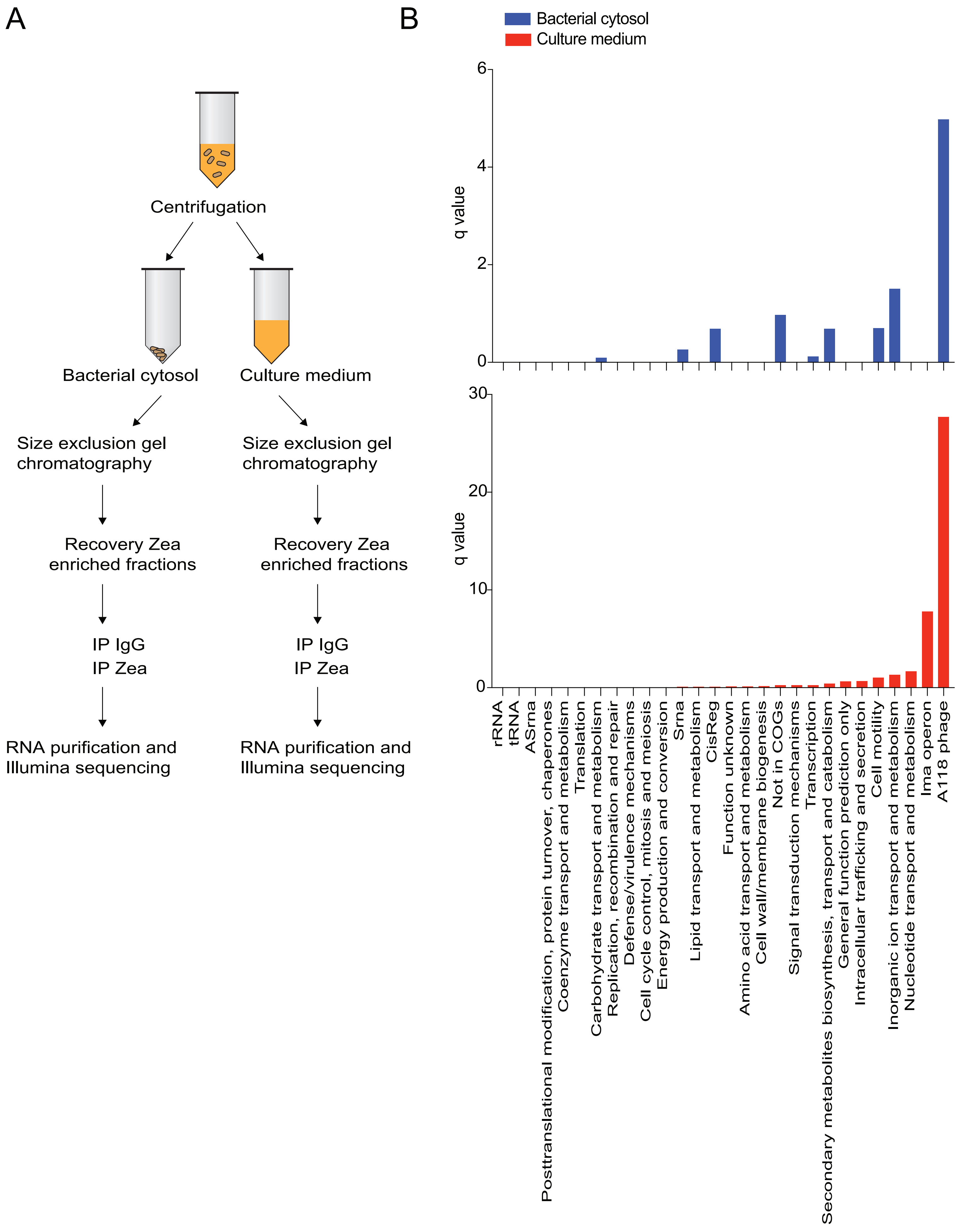
A
Centrifugation
Bacterial cytosol
Culture medium
Size exclusion gel chromatography
Size exclusion gel chromatography
Recovery Zea enriched fractions
Recovery Zea enriched fractions
IP IgG
IP Zea
IP IgG
IP Zea
RNA purification and Illumina sequencing
RNA purification and Illumina sequencing
B
Bacterial cytosol
Culture medium
q value
6
4
2
0
q value
30
20
10
0
rRNA
tRNA
ASrna
Posttranslational modification, protein turnover, chaperones
Coenzyme transport and metabolism
Translation
Carbohydrate transport and metabolism
Replication, recombination and repair
Defense/virulence mechanisms
Cell cycle control, mitosis and meiosis
Energy production and conversion
Srna
Lipid transport and metabolism
CisReg
Function unknown
Amino acid transport and metabolism
Cell wall/membrane biogenesis
Not in COGs
Signal transduction mechanisms
Transcription
Secondary metabolites biosynthesis, transport and catabolism
General function prediction only
Intracellular trafficking and secretion
Cell motility
Inorganic ion transport and metabolism
Nucleotide transport and metabolism
Ima operon
A118 phage

Figure S3

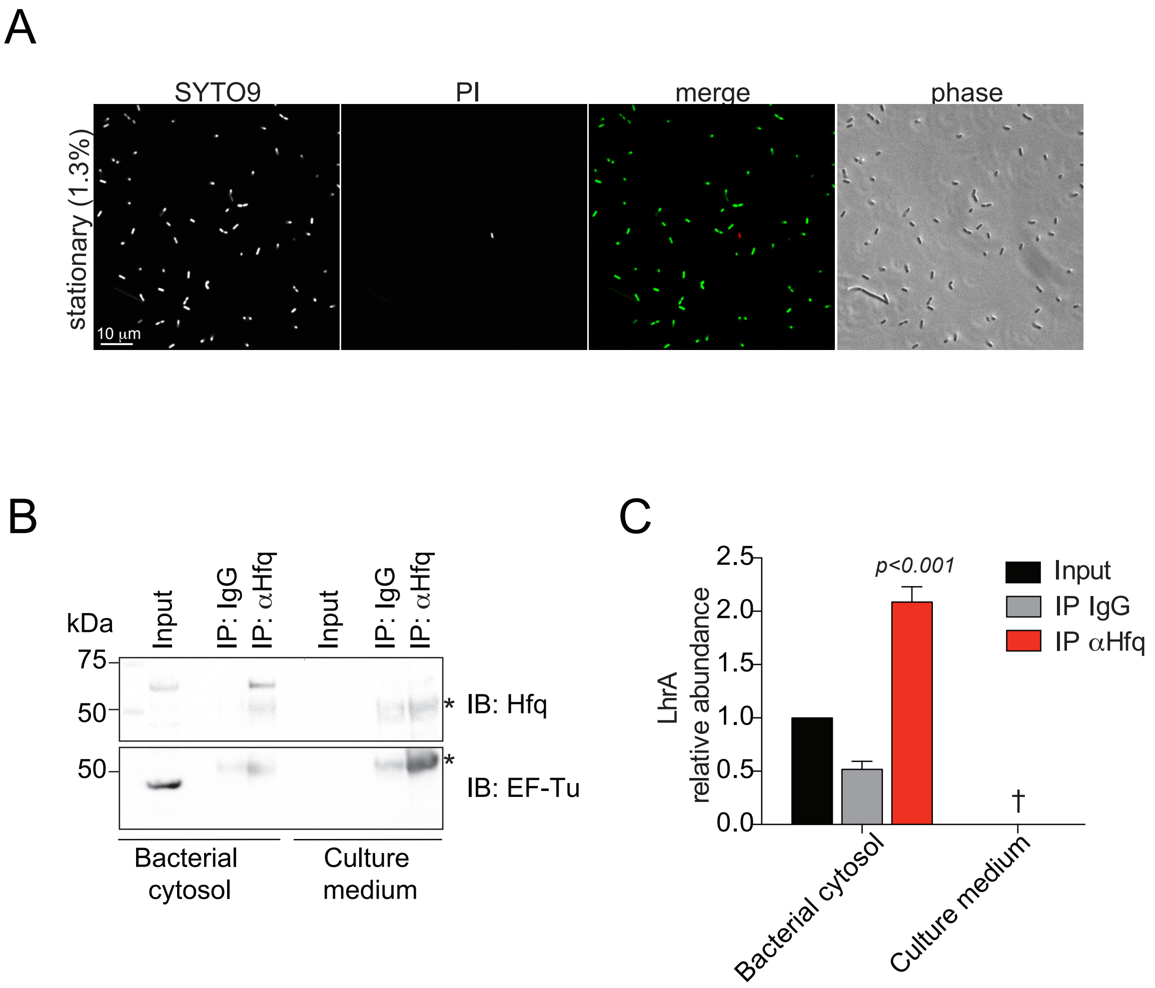
A
SYTO9
PI
merge
phase
stationary (1.3%)
10 µm
B
kDa
Input
IP: IgG
IP: αHfq
Input
IP: IgG
IP: αHfq
75
50
50
* IB: Hfq
* IB: EF-Tu
Bacterial cytosol
Culture medium
C
LhrA relative abundance
2.5
2.0
1.5
1.0
0.5
0.0
p<0.001
†
Input
IP IgG
IP αHfq
Bacterial cytosol
Culture medium

Figure S4

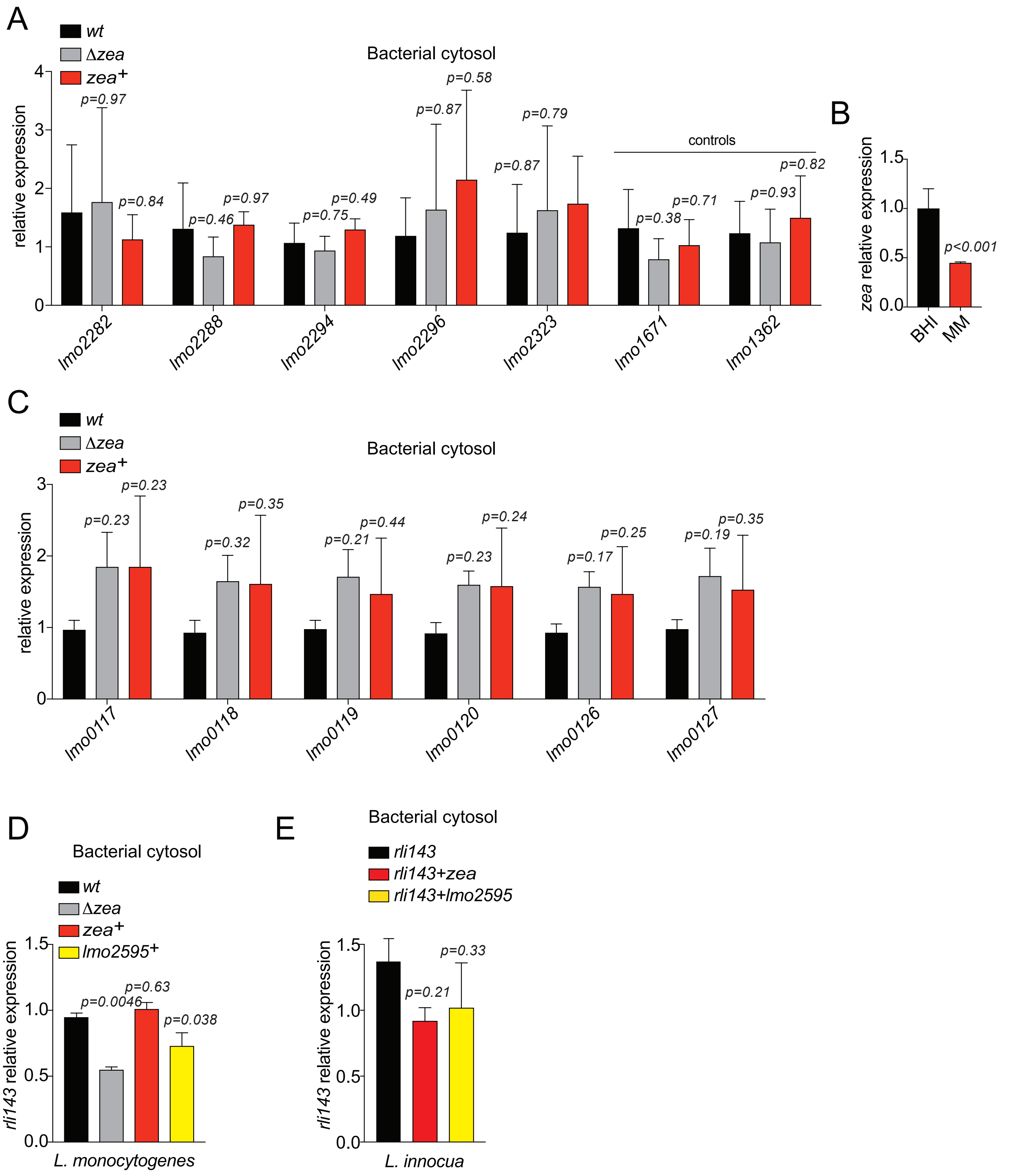

A
wt
Δzea
zea+
Bacterial cytosol
relative expression
controls
p=0.97
p=0.84
p=0.46
p=0.97
p=0.75
p=0.49
p=0.87
p=0.58
p=0.87
p=0.79
p=0.38
p=0.71
p=0.93
p=0.82
lmo2282
lmo2288
lmo2294
lmo2296
lmo2323
lmo1671
lmo1362
B
zea relative expression
p<0.001
BHI
MM
C
wt
Δzea
zea+
Bacterial cytosol
relative expression
p=0.23
p=0.23
p=0.32
p=0.35
p=0.21
p=0.44
p=0.23
p=0.24
p=0.17
p=0.25
p=0.19
p=0.35
lmo0117
lmo0118
lmo0119
lmo0120
lmo0126
lmo0127
D
Bacterial cytosol
wt
Δzea
zea+
lmo2595+
rli143 relative expression
p=0.0046
p=0.63
p=0.038
L. monocytogenes
E
Bacterial cytosol
rli143
rli143+zea
rli143+lmo2595
rli143 relative expression
p=0.21
p=0.33
L. innocua

Figure S5

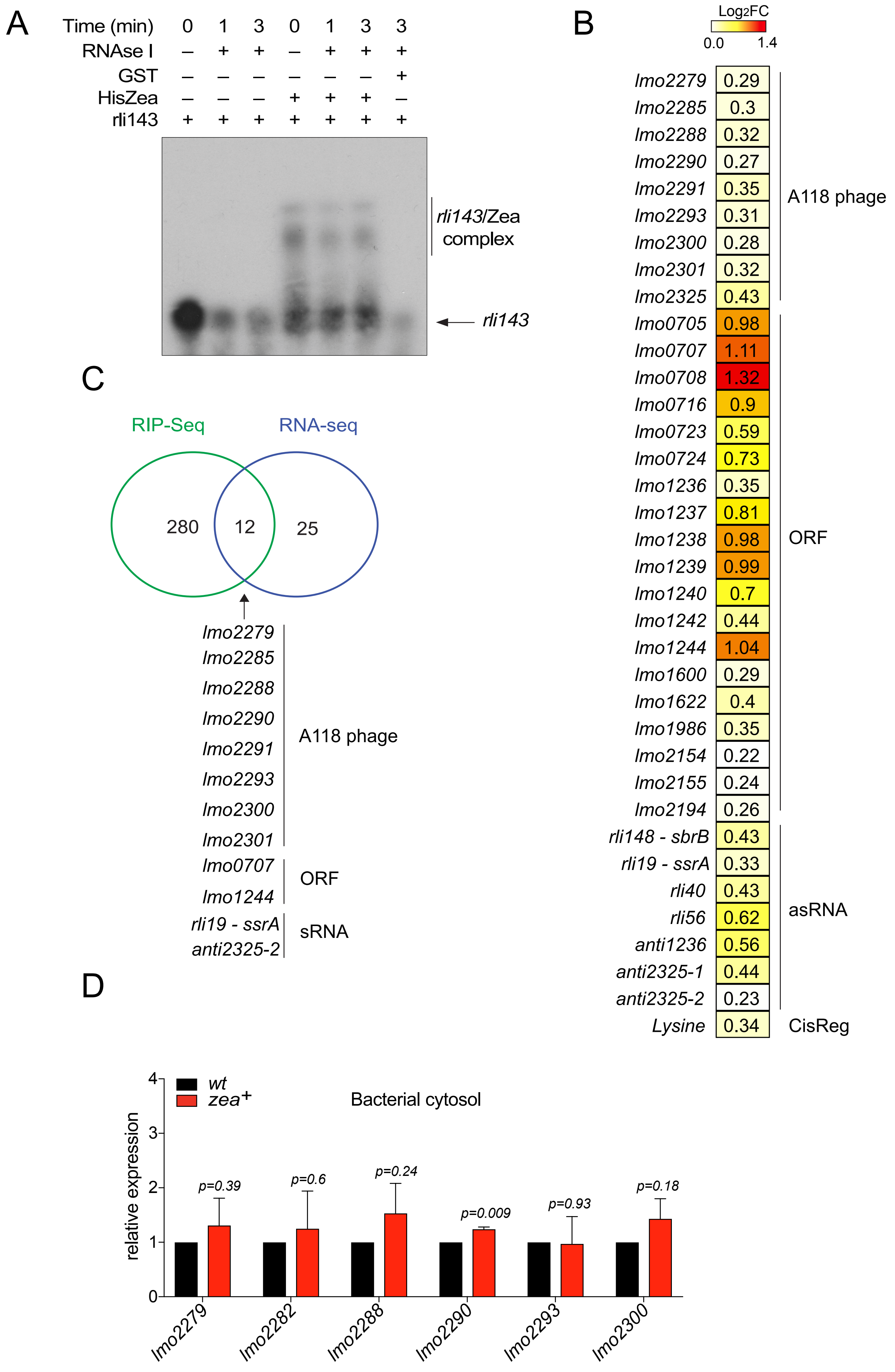

A
Time (min) 0 1 3 0 1 3 3
RNAse I – + + – + + +
GST – – – – – – +
HisZea – – – + + + –
rli143 + + + + + + +
rli143/Zea complex
rli143
B
Log2FC
0.0 1.4
lmo2279 0.29
lmo2285 0.3
lmo2288 0.32
lmo2290 0.27
lmo2291 0.35
lmo2293 0.31
lmo2300 0.28
lmo2301 0.32
lmo2325 0.43
A118 phage
lmo0705 0.98
lmo0707 1.11
lmo0708 1.32
lmo0716 0.9
lmo0723 0.59
lmo0724 0.73
lmo1236 0.35
lmo1237 0.81
lmo1238 0.98
lmo1239 0.99
lmo1240 0.7
lmo1242 0.44
lmo1244 1.04
lmo1600 0.29
lmo1622 0.4
lmo1986 0.35
lmo2154 0.22
lmo2155 0.24
lmo2194 0.26
ORF
rli148 - sbrB 0.43
rli19 - ssrA 0.33
rli40 0.43
rli56 0.62
anti1236 0.56
anti2325-1 0.44
anti2325-2 0.23
asRNA
Lysine 0.34
CisReg
C
RIP-Seq
RNA-seq
280
12
25
lmo2279
lmo2285
lmo2288
lmo2290
lmo2291
lmo2293
lmo2300
lmo2301
A118 phage
lmo0707
lmo1244
ORF
rli19 - ssrA
anti2325-2
sRNA
D
wt
zea+
Bacterial cytosol
relative expression
0
1
2
3
4
p=0.39
p=0.6
p=0.24
p=0.009
p=0.93
p=0.18
lmo2279
lmo2282
lmo2288
lmo2290
lmo2293
lmo2300

Figure S6

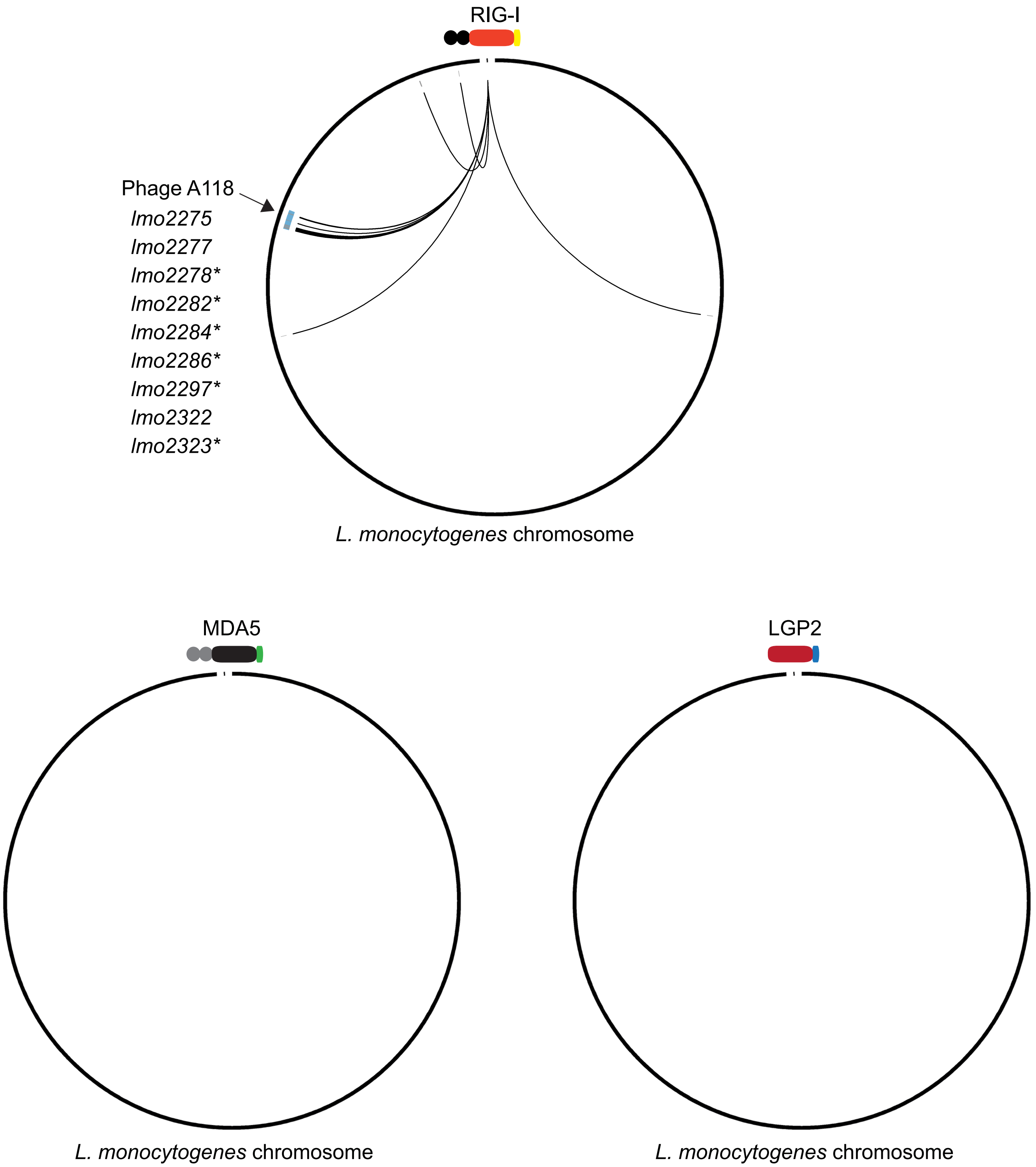

RIG-I
Phage A118
lmo2275
lmo2277
lmo2278*
lmo2282*
lmo2284*
lmo2286*
lmo2297*
lmo2322
lmo2323*
L. monocytogenes chromosome
MDA5
LGP2
L. monocytogenes chromosome
L. monocytogenes chromosome

Figure S7

A

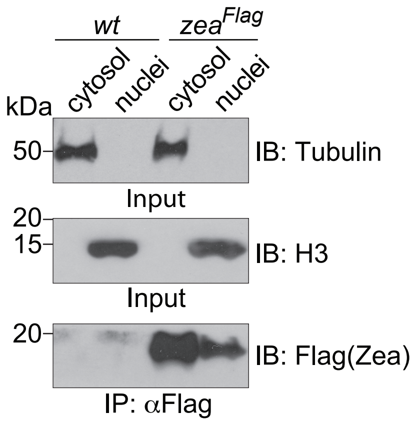
wt
zeaFlag
cytosol
nuclei
cytosol
nuclei
kDa
50
IB: Tubulin
Input
20
15
IB: H3
Input
20
IB: Flag(Zea)
IP: αFlag

B

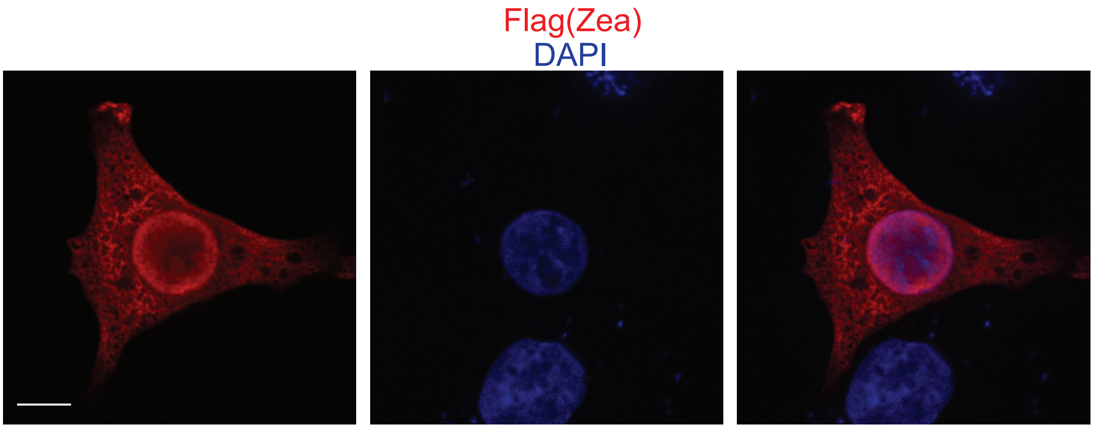
Flag(Zea)
DAPI

Table S1. Identification of Zea-binding RNAs, related to Figure 2

BACTERIAL CYTOSOL	CULTURE MEDIUM
ORF: 89% (Phage 27.7%)	ORF: 94.5% (Phage 14.4%; Lma-monocin locus 3%)
ncRNAs: 11%	ncRNA: 5.5%

Table S2. List of oligonucleotides used in this study, related to STAR Methods

Lmo2282 qPCR rv CCCATCTTTTCTTGTCTTCTTTCCAAATACTTC
Lmo2288 qPCR fw GACAACGTTCTAAGAGTGATTGATTTAACGG
Lmo2288 qPCR rv GCGAGGTTAGCTCTGAACACATCAGAC
lmo2290 qPCR fw GCGTTTGGTTGCCGCATCGCTTATGATG
lmo2290 qPCR rv CCACTGGCAATACAGAAGCATTAATAGTAAATG
lmo2294 qPCR fw GAAAGTAGCAAAACCGATAACAAATGC
lmo2294 qPCR rv CCCGCGATTTGATTTGATTTTGAGAAATCAACAC
lmo2296 qPCR fw CACAGCGGGCGGATTACTTGTTAATATGC
lmo2296 qPCRrv GCATTCAAAGGGTCGTCTCCCGAGATG
lmo2323 qPCR fw GCCGCGATGCAAGTTTTAGCAGAAACTC
lmo2323 qPCR rv CGCGGTTCGTTGTAACTATCAATTAGC
rli18 qPCR fw GATGATATAGCAGGAGATTGCTATG
rli18 qPCR rv GCGTGTTTTACATCCAGTATGGATAG
lmo2615 qPCR fw AACCGTGTTGCTAAAGTAGTTAAAGGTGG
lmo2615 qPCR rv GCATCCTCAACAGCTTTACGGATAGC
lmo1671 qPCR fw TGGTTGCTCAGACGAGAGCGACACAAAAG
lmo1671 qPCR rv CAGAACCAGGCGGATAAATGCTGTG
lmo1362 qPCR fw GGTAGTGCCTCACTTGAAGATTCTCTCG
lmo1362 qPCR rv CACCATCCGCTTCAAAAGGAATTTCTTCTC
Zea qPCR fw GGGAATATAGCTCAAGAGAAAGAAATTG
Zea qPCR rv ACCCTTACTAACATTAATCTTAGCATTAAACGA
rli143 qPCR fw AGTAGAAAAGACCAATGTGGGTTCT
rli143 qPCR rv CAGTTTTTCATCTATAGGTTAATTGCATTAC
lmo0117 qPCR fw CCTACAACGATGATCGAGATGATTG
lmo0117 qPCR rv GCGTATCTATCTTTTCGACTCCC
lmo0118 qPCR fw GCCAACAAGGTCTAACTGTAAAC
lmo0118 qPCR rv CGAAATATTTCGCCAATTCTTTGTGAG
lmo0119 qPCR fw GGAATTAACATCAGGGATTAGTTTAGTTGC
lmo0119 qPCR rv CAGGTACTAAGCCTTTTTCTTCCAC
lmo0120 qPCR fw GAAATGAACCATGATTTTGATTTGGA
lmo0120 qPCR rv CGAAGCATAGTAGATTCAAAAATCACC
lmo126 qPCR fw CGGGTTGACAGAATATGTGTCAG
lmo126 qPCR rv CTCATAAAGCCATCCGTAGTGTATG
lmo127 qPCR fw TTGTGTCGGTTTCAGAGGAAGAG
lmo127 qPCR rv CACCGTTCCTTCACCTATCGTATC
LhrA qPCR fw GGGGATAAGACCCTATCATGGTTATAATGAC
LhrA qPCR rv GACGCTCGAAATAAACATTTCCAGCG
IFNβ qPCR fw CAGCAATTTTCAGTGTCAGAAGC
IFNβ qPCR rv TCATCCTGTCCTTGAGGCAGT
IFNγ qPCR fw GCATCGTTTTGGGTTCTCTTG
IFNγ qPCR rv AGTTCCATTATCCGCTACATCTG
IL8 qPCR fw CAAGAGCCAGGAAGAAACCA
IL8 qPCR rv AGCACTCCTTGGCAAAACTG
rli143 T7 fw GAATTTAATACGACTCACTATAGGAGTAGAAAAGACCAATGTGGGTTC
rli143 T7 rv ACAGTTTTTCATCTATAGGTTAATTGC
rli92 T7 fw GAATTTAATACGACTCACTATAGGGTGTAAAATGCAGCTTGAGTAAAATGAATGAG
rli92 T7 rv GTATAAAACAAACGTGACGTAAAACCAAATC
