# Supplementary material for: An RNA-Binding Protein Secreted by a Bacterial Pathogen Modulates RIG-I Signaling
Source: Cell Host Microbe. 2019 Dec 11;26(6):823–835.e11. doi: 10.1016/j.chom.2019.10.004 (PMC6907008; doi:10.1016/j.chom.2019.10.004)
Supplement: Document S2. Article plus Supplemental Information [file mmc2.pdf]

Article

Cell Host & Microbe

An RNA-Binding Protein Secreted by a Bacterial Pathogen Modulates RIG-I Signaling

Graphical Abstract

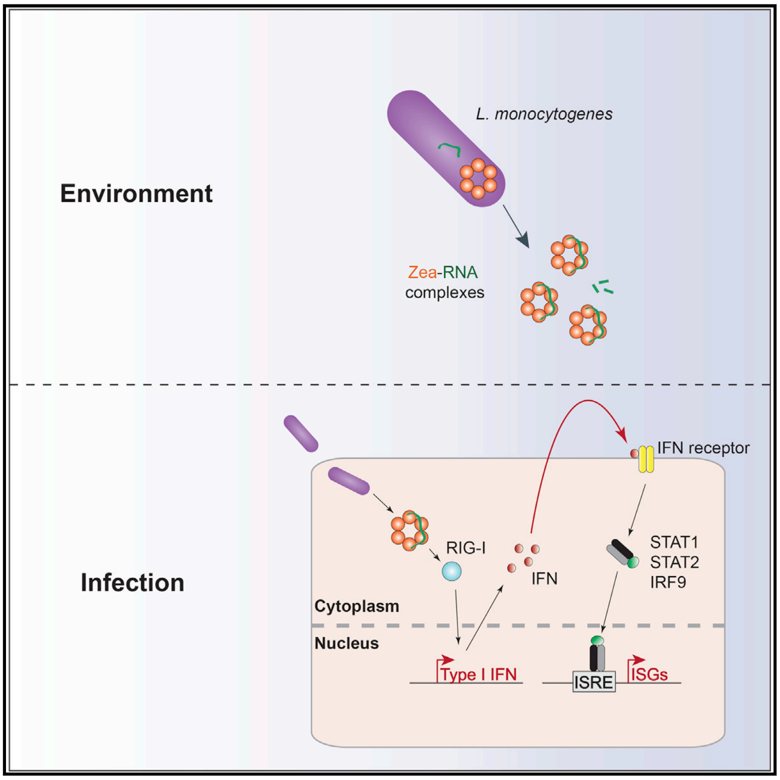

Authors

Alessandro Pagliuso, To Nam Tham, Eric Allemand, ..., Alice Lebreton, Anastassia V. Komarova, Pascale Cossart

Correspondence

alessandro.pagliuso@inra.fr (A.P.), pcossart@pasteur.fr (P.C.)

In Brief

Extracellular RBPs have only been described in eukaryotes. Pagliuso et al. report that *L. monocytogenes* secretes an RNA-binding protein, Zea, which associates extracellularly with *L. monocytogenes* RNA. During infection, Zea binds RIG-I and modulates the type I IFN response. These results reveal that RNA can traffic between organisms via RBPs.

Highlights

- *L. monocytogenes* secretes an RNA-binding protein, Zea
- Zea binds and protects *L. monocytogenes* RNA, resulting in extracellular RNA accumulation
- During infection, Zea binds RIG-I and modulates RIG-I-dependent IFN response
- Zea plays a role in *L. monocytogenes* virulence in mice

Pagliuso et al., 2019, Cell Host & Microbe *26*, 823–835
December 11, 2019 © 2019 The Authors. Published by Elsevier Inc.
https://doi.org/10.1016/j.chom.2019.10.004

Cell Host & Microbe
Article

CellPress

An RNA-Binding Protein Secreted by a Bacterial Pathogen Modulates RIG-I Signaling

Alessandro Pagliuso,[1,2,3,16,*] To Nam Tham,[1,2,3,17] Eric Allemand,[4,17] Stevens Robertin,[1,2,3,17] Bruno Dupuy,[5] Quentin Bertrand,[6] Christophe Bécavin,[7] Mikael Koutero,[1,2,3] Valérie Najburg,[8,9] Marie-Anne Nahori,[1,2,3] Frédéric Tangy,[8,9] Fabrizia Stavru,[1,2,3] Sergey Bessonov,[10,11,12] Andréa Dessen,[6,13] Christian Muchardt,[4] Alice Lebreton,[14,15] Anastassia V. Komarova,[8,9] and Pascale Cossart[1,2,3,18,*]

[1]Unité des Interactions Bactéries-Cellules, Institut Pasteur, Paris, France
[2]U604 Inserm, Paris, France
[3]USC2020 INRA, Paris, France
[4]Unité de régulation épigénétique, Institut Pasteur, UMR3738 CNRS, Paris, France
[5]Laboratoire Pathogenèse des Bactéries Anaérobies, Institut Pasteur, Paris, Université de Paris, Paris, France
[6]Université Grenoble Alpes, CNRS, CEA, Institut de Biologie Structurale (IBS), Bacterial Pathogenesis Group, Grenoble, France
[7]Hub de bioinformatique et biostatistique - Centre de Bioinformatique, Biostatistique et Biologie Intégrative, Unité mixte de Service et Recherche 3756 Institut Pasteur - Centre National de la Recherche Scientifique, Paris 75015, France
[8]Unité de Génomique Virale et Vaccination, Institut Pasteur, Paris 75015, France
[9]CNRS UMR-3569, Paris, France
[10]Department I of Internal Medicine, University Hospital Cologne, Cologne, Germany
[11]Cologne Excellence Cluster on Cellular Stress Response in Aging-Associated Diseases (CECAD), University of Cologne, Cologne, Germany
[12]Department of Translational Epigenetics and Tumor Genetics, University Hospital Cologne, Cologne, Germany
[13]Brazilian Biosciences National Laboratory (LNBio), CNPEM, Campinas, SP, Brazil
[14]Équipe Infection et Devenir de l'ARN, Institut de biologie de l'Ecole normale supérieure (IBENS), Ecole normale supérieure, CNRS, Inserm, PSL Université Paris, Paris 75005, France
[15]INRA, IBENS, 75005 Paris, France
[16]Present address: Micalis Institute, Inra, AgroParisTech, Université Paris-Saclay, Equipe Epigénétique et Microbiologie Cellulaire, Jouy-en-Josas, France
[17]These authors contributed equally
[18]Lead Contact
*Correspondence: alessandro.pagliuso@inra.fr (A.P.), pcossart@pasteur.fr (P.C.)
https://doi.org/10.1016/j.chom.2019.10.004

SUMMARY

RNA-binding proteins (RBPs) perform key cellular activities by controlling the function of bound RNAs. The widely held assumption that RBPs are strictly intracellular has been challenged by the discovery of secreted RBPs. However, extracellular RBPs have been described in eukaryotes, while secreted bacterial RBPs have not been reported. Here, we show that the bacterial pathogen *Listeria monocytogenes* secretes a small RBP that we named Zea. We show that Zea binds a subset of *L. monocytogenes* RNAs, causing their accumulation in the extracellular medium. Furthermore, during *L. monocytogenes* infection, Zea binds RIG-I, the non-self-RNA innate immunity sensor, potentiating interferon-β production. Mouse infection studies reveal that Zea affects *L. monocytogenes* virulence. Together, our results unveil that bacterial RNAs can be present extracellularly in association with RBPs, acting as "social RNAs" to trigger a host response during infection.

INTRODUCTION

RNA-binding proteins (RBPs) are found in all living organisms. By binding RNAs, RBPs assemble in ribonucleoprotein complexes that dictate the fate and the function of virtually every cellular RNA molecule. In bacteria, RBPs interact with their cognate RNAs via classical RNA-binding domains (RBDs), structurally well-defined signatures that recognize specific RNA sequences and/or motifs (reviewed in Holmqvist and Vogel, 2018). Previously thought to be mainly involved in transcriptional regulation, bacterial RBPs have now been implicated in a wide variety of cellular processes such as translation, RNA turnover, decay, processing, and stabilization (Holmqvist and Vogel, 2018). Although bacterial RBPs regulate vital functions in bacterial physiology, their number remains limited. It is therefore conceivable that many bacterial RBPs remain to be discovered.

One feature of all bacterial RBPs described so far is their exquisite intracellular localization. Few extracellular RBPs have been described only in eukaryotes and shown to stabilize RNA in the extracellular *milieu* and participate in cell-to-cell communication (Arroyo et al., 2011; Vickers et al., 2011; Wang et al., 2010; Shurtleff et al., 2016; Maori et al., 2019). At present, no secreted RBPs have been identified in bacteria. A recent study screened

This is an open access article under the CC BY license (http://creativecommons.org/licenses/by/4.0/).

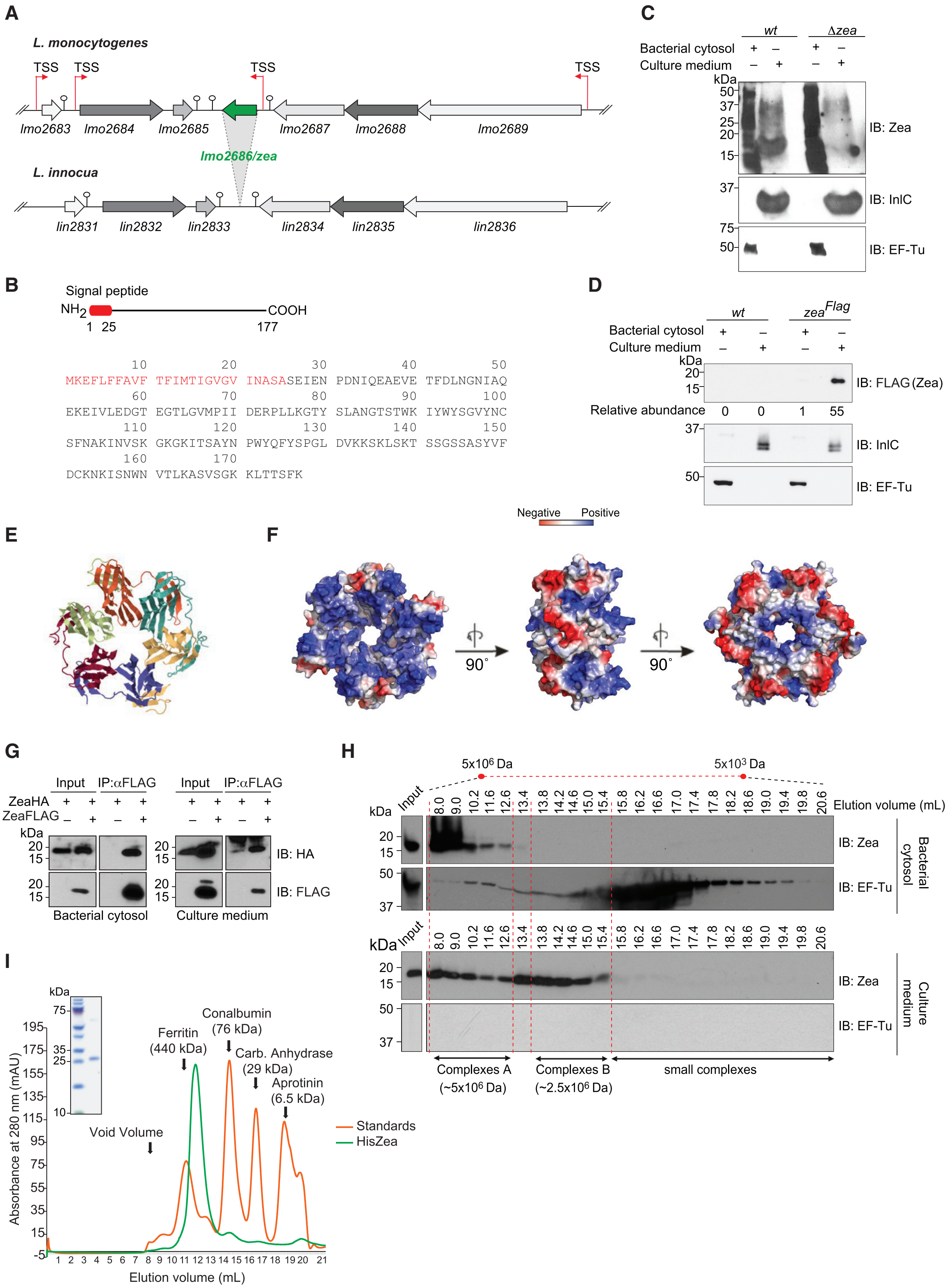

Figure 1. Zea Is a Secreted Oligomeric Protein of *L. monocytogenes*

(A) Syntheny analysis of the *lmo2686/zea*-containing genomic locus between *L. monocytogenes* and *L. innocua*. Arrows and stem and circle represent the transcriptional start sites (TSSs) and the transcriptional terminators, respectively.

(B) Schematic representation and primary sequence of the Zea protein. The N-terminal signal peptide is highlighted in red.

(legend continued on next page)

thousands of secreted effectors of Gram-negative symbionts and bacterial pathogens for the presence of known RBDs and failed to unambiguously identify any RBPs (Tawk et al., 2017). It is therefore likely that secreted bacterial RBPs harbor unconventional RBDs, which render them undetectable by using conservation-based searches.

In this study, we report the identification of a secreted bacterial RBP, the *Listeria monocytogenes* protein Lmo2686. We provide evidence that Lmo2686 is secreted in the culture supernatant, where it is associated with a subset of *L. monocytogenes* RNAs. Protein sequence analysis of Lmo2686 revealed the absence of any canonical RBD, suggesting a non-canonical mode of RNA binding. We show that Lmo2686 induces the extracellular accumulation of its RNA targets, possibly by protecting them from degradation. Furthermore, during infection of mammalian cells, Lmo2686 interacts with RIG-I and modulates RIG-I-dependent type I interferon (IFN) response. We further show that Lmo2686 affects *L. monocytogenes* virulence *in vivo*. Based on these findings, we propose to rename this protein Zea—as Zea, also known as Hecate, is an ancient Greek goddess who protected and guided the travelers. The presence of Zea orthologs in other bacterial species revealed that secretion of RBPs is a conserved phenomenon in prokaryotes.

RESULTS

Zea Is a Secreted Protein of *L. monocytogenes*

The *lmo2686/zea* open-reading frame is 534 bp long (Figure 1A). *zea* is found in half of the *L. monocytogenes* strains sequenced to date as well as in the animal pathogen *Listeria ivanovii* (Bécavin et al., 2017). Orthologs of *zea* are also found in other species, mainly bacteria of the genus *Bacillus* (Figure S1). *zea* is absent from the genome of the nonpathogenic species *L. innocua* (Glaser et al., 2001) and *Listeria marthii* (Graves et al., 2010), which suggests that it may contribute to *L. monocytogenes* virulence (Figure 1A).

RNA sequencing (RNA-seq) data have revealed a transcriptional start site upstream of the start codon of *zea* (Figure 1A) (Wurtzel et al., 2012). *zea* appears constitutively expressed at 37°C, albeit at low levels, and is slightly upregulated under microaerophilic conditions and at 4°C (Bécavin et al., 2017; Wurtzel et al., 2012).

The *zea* gene encodes a protein of 177 amino acids (aa) (Figure 1B). Analysis of the Zea protein sequence predicted the presence of an N-terminal signal peptide of 25 aa for Sec-mediated secretion, resulting in a putative 152 aa-mature protein with a basic isoelectric point (pI = 8.4) (Figure 1B). Of note, the signal peptide is conserved in almost all the Zea orthologs, suggesting that the major function of the protein is outside bacteria (Figure S1). We could not identify any other domain of known function.

The presence of a signal peptide prompted us to test whether Zea could be secreted. We generated three antibodies against three peptides of the C terminus of the protein and used them to assess the presence of Zea in the *L. monocytogenes* cytosol and in the culture medium. Immunoblot analysis revealed that Zea could be recovered from the culture medium, indicating secretion of the protein (Figure 1C). Culture medium collected from the *zea*-deleted strain (*Δzea*) did not show any immunoreactive band, thus confirming the specificity of our antibodies. The secretion of Zea was also confirmed by engineering a *L. monocytogenes* strain carrying a chromosomally integrated copy of the C-terminally FLAG-tagged *zea* gene under the control of a constitutive promoter (zea^{FLAG}) (Figure 1D). Quantitative analysis of the distribution of Zea between bacterial cytosol and culture medium in stationary phase revealed a strong accumulation in the extracellular medium, indicating that the protein was efficiently secreted (Figure 1D).

Zea Is an Oligomeric Protein that Interacts with RNA

The structure of Zea was previously solved by X-ray crystallography at a resolution of 2.75 Å and deposited in the Protein Data Bank (PDB) by Minasov and colleagues (PDB: 4K15). Zea is a toroid-shaped homohexamer in which every monomer essentially contacts the neighboring molecule via a beta sheet-hairpin-beta sheet unit (Figure 1E). As this structure is not shared by any other polypeptide of known function, the role of Zea and its orthologs is currently unknown.

Nevertheless, we noticed several other proteins that assemble as a torus (e.g., in Hfq) have the intrinsic capability to bind RNA (Babitzke et al., 1995; Lee et al., 2007; Antson et al., 1995; Vogel and Luisi, 2011; Thomsen and Berger, 2009). Interestingly, Zea shows a positively charged surface on one side of the torus due to the presence of several lysine residues, which might accommodate the negatively charged RNA (Figure 1F). These features led us to hypothesize that Zea might bind RNA.

Before addressing this hypothesis, we sought to verify whether the oligomeric state of Zea observed by X-ray crystallography also existed under physiological conditions, ruling out possible crystallization artifacts. We used three different approaches: (1) co-immunoprecipitation of hemagglutinin (HA)- and FLAG-tagged versions of Zea (Figure 1G), (2) size-exclusion chromatography of *L. monocytogenes* cytosol and culture medium (Figure 1H), and (3) size-exclusion gel chromatography of recombinant His-tagged Zea expressed and purified from *E. coli* (Figure 1I). Collectively, our data show that Zea has a high tendency to oligomerize, in line with the hexameric structure

(C and D) Bacterial cytosol and culture medium from (C) *L. monocytogenes* WT and *Δzea* strains and from (D) WT and a FLAG-tagged Zea-overexpressing *L. monocytogenes* strain (zea^{FLAG}) were immunoblotted with the indicated antibodies (n = 2).

(E) Ribbon diagram of hexameric Zea.

(F) Electrostatic potential surface representation of hexameric Zea.

(G) Immunoprecipitation (IP) of Zea with an anti-FLAG antibody from bacterial cytosol and culture medium from a *L. monocytogenes* strain co-overexpressing Zea^{FLAG} and Zea^{HA} (n = 2). Immunoblot of input and immunoprecipitated proteins were probed with an anti-FLAG and anti-HA antibodies.

(H) ZeaFLAG elution profile from size exclusion gel chromatography (n = 2).

(I) 280 nm (mAU) absorbance monitoring of a gel filtration profile of recombinant purified HisZea (green line; n = 2). The elution profile of protein markers is indicated with the orange line. Purified HisZea was analyzed by SDS-PAGE and Coomassie blue staining (top left-hand panel).

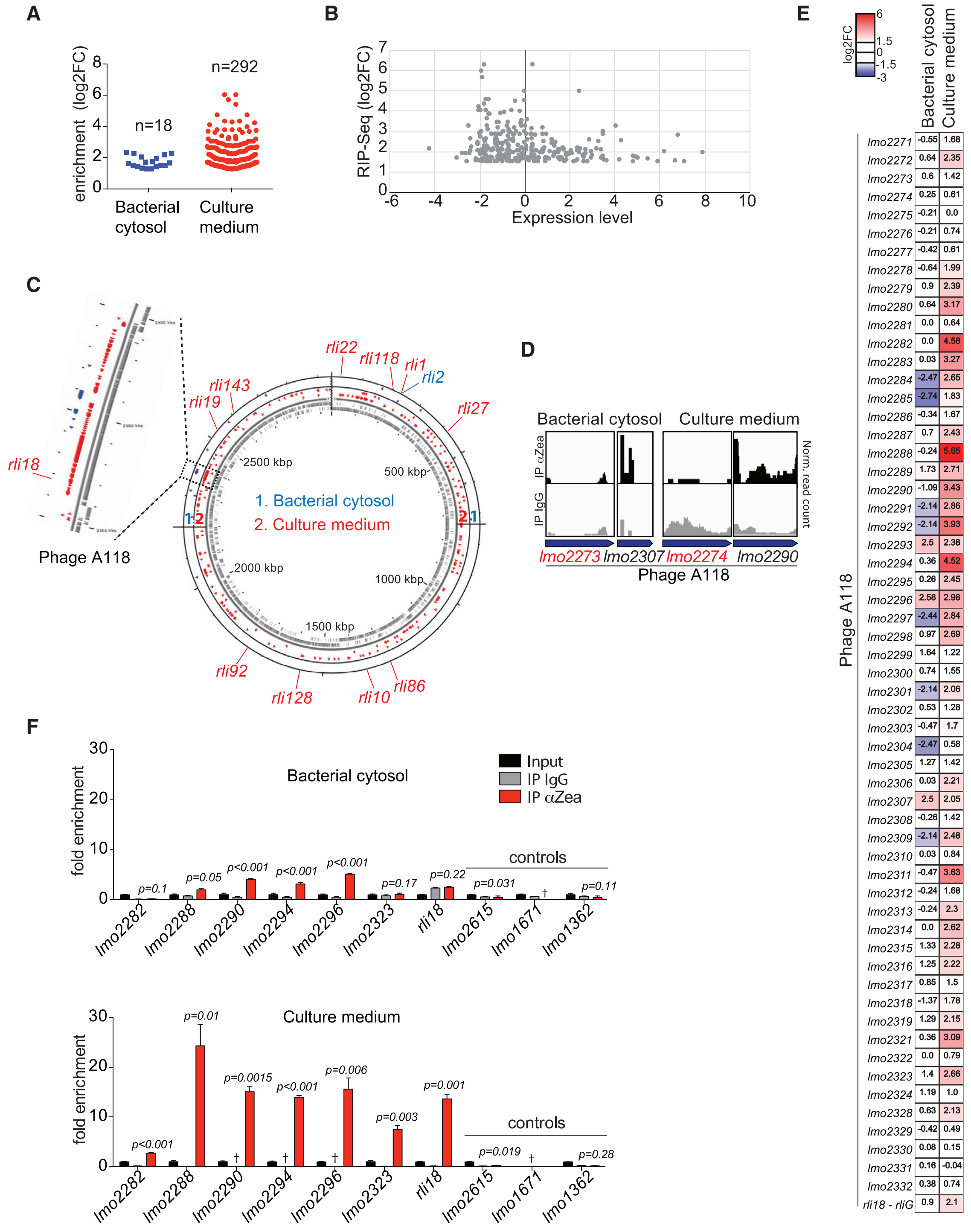

Figure 2. Zea Associates with RNA

(A) Enrichment of Zea-bound RNAs (n) from bacterial cytosol and culture medium. Blue squares and red circles depict individual RNAs. The y axis shows the enrichment of the Zea-interacting RNAs relative to immunoprecipitation with IgG.

(B) Expression of *L. monocytogenes* RNAs grown in BHI at stationary phase measured by tiling array compared with the enrichment of the Zea-bound RNAs.

(C) Circular genome map of *L. monocytogenes* showing the position of the Zea-interacting RNAs. The first two circles from the inside show the genes encoded on the + (inner track) and – (outer track) strands, respectively. The positions of Zea-interacting small RNAs (rlis) are pointed at outside of the circular map. Dotted lines highlight the phage A118 locus.

(D) Examples of normalized read coverage (reads per million) visualized by IGV from Zea and control (IgG) IP for a selection of phage A118 genes (blue arrows). Gene names marked in red show no significant enrichment in the Zea IP.

(legend continued on next page)

shown by X-ray crystallography. We noticed, however, that the molecular mass of the recombinant His-tagged Zea (HisZea) exceeded that of the hexameric Zea, indicating that high molecular weight assemblies composed of several hexameric units or, potentially, other components are formed.

We then examined whether Zea could bind RNA. We performed RNA immunoprecipitation (IP) of cytosolic extract and culture supernatant followed by high-throughput sequencing (RIP-seq) (Figure S2A). Given the low amount of Zea protein produced *in vitro*, we made use of a Zea-overexpressing strain (*zea*$^+$). Zea was immunoprecipitated from *L. monocytogenes* cytosol and culture medium, and the Zea-bound RNAs were subsequently extracted and sequenced. As a control, we performed a mock IP using an unrelated antibody of the same isotype. Remarkably, RIP-seq analysis revealed the presence of *L. monocytogenes* RNAs almost exclusively in the culture medium compared with the control samples (Figure 2A), indicating that Zea can form complexes with RNA extracellularly. An enrichment threshold of $\log_2$ fold change ($\log_2$FC) >1.5, corresponding to an almost 3-fold increase, was used for the identification of Zea-associated RNAs. Importantly, the enrichment of specific RNAs in the Zea IP was uncorrelated to their expression levels (Figure 2B) (Bécavin et al., 2017). Zea preferentially bound a subset of protein-coding mRNAs and small regulatory RNAs, to a lesser extent (Table S1).

We then analyzed the genomic distribution of Zea-bound RNAs on the *L. monocytogenes* chromosome (Figure 2C). It was striking that there was one region particularly overrepresented. This locus contains the prophage A118 (Figures 2C–2E). The phage A118 is a temperate phage belonging to the *Syphoviridae* family of double-stranded DNA bacterial viruses (Dorscht et al., 2009). Cluster of Orthologous Genes (COG) classification highlighted the phage A118 RNA as the most enriched class of Zea-bound RNAs in the culture medium (Figure S2B).

To validate the interaction of phage A118 RNA with Zea found by RIP-seq (Figures 2C–2E), we next performed RNA IP coupled with quantitative PCR (RIP-qPCR) analysis. RIP-qPCR confirmed a strong association of Zea to phage RNA, but we could not find any binding to control transcripts that were not enriched in our RIP-seq dataset (Figure 2F). In agreement with the RIP-seq data, the enrichment of phage RNA in the culture medium fraction was particularly strong, indicating that the phage RNA accumulates extracellularly together with Zea (Figure 2F). Taken together, our data revealed that Zea is an oligomeric protein that is found associated in the extracellular compartment with a subset of *L. monocytogenes* RNAs enriched in phage RNA.

Zea Directly Binds *L. monocytogenes* RNA

We next investigated whether Zea could directly bind RNA. We first performed electrophoretic mobility gel shift assay (EMSA) by using recombinant HisZea and *in vitro*-transcribed radiolabeled RNA. We selected *rli143* and *rli92*, two small RNAs that showed a significant enrichment in the RIP-seq dataset (8- and almost 3-fold enrichment compared with control immunoglobulin G [IgG] IP, respectively; see Figure 3A) and have a small size, which is appropriate for *in vitro* transcription. Incubation of *rli143* or *rli92* with HisZea produced several shifts, most likely due to the binding of different Zea oligomers to RNA (Figures 3A and 3B). Importantly, the binding of Zea with both *rli143* and *rli92* was specific, as it was displaced by the addition of increasing amounts of each unlabeled small RNA (Figures 3C and 3D).

To further prove direct binding of Zea with its target RNAs, we performed an RNA pull-down assay using *in vitro*-transcribed biotinylated RNA and recombinant HisZea. Here, in addition to *rli143* and *rli92*, we tested two other small RNAs (*rli18* and *rli1*), which also displayed specific binding to Zea in the RIP-seq dataset (Figure 2C). As a control, we employed a small RNA (*rli80*) that was not specifically bound by Zea. Using this alternative approach, we confirmed binding above background level for *rli143* (Figure 3E), which also showed the highest enrichment in the RIP-seq dataset. Collectively, these data clearly indicate that Zea directly binds RNA.

Extracellular Zea-Bound RNAs Do Not Derive from Bacterial Lysis

It was important to verify that the extracellular RNAs in complex with Zea were not due to bacterial lysis. Thus, we first analyzed the presence of one abundant cytosolic protein of *L. monocytogenes* (EF-Tu) in the culture medium. EF-Tu was undetectable in the culture medium, indicating that bacterial lysis under our experimental conditions was negligible (Figure 1H). As bacteria lyse after death, we also quantified both live and dead bacteria. Confocal microscopy analysis revealed less than 2% of dead bacteria, further confirming minimal bacterial lysis (Figure S3A). Finally, we designed an experiment in which we used the strict intracellular localization of the RBP Hfq and its RNA targets as a readout of bacterial lysis. In *L. monocytogenes*, Hfq has been shown to bind three small RNAs, *LhrA*, *LhrB*, and *LhrC* (Christiansen et al., 2006). We reasoned that if bacterial lysis occurred, we should find Hfq complexed to its RNA targets in the medium. We thus grew *L. monocytogenes* under the conditions used for the Zea RIP-seq experiment and then immunoprecipitated Hfq from the bacterial cytosol and culture medium by using an anti-Hfq antibody (Christiansen et al., 2006). Hfq was recovered from the bacterial cytosol but was undetectable in the culture medium, indicating minimal bacterial lysis (Figure S3B). RNA was extracted from the immunopurified Hfq ribonucleoprotein complexes and used to assess the abundance of the Hfq targets by qPCR. Given the low expression of *LhrB* and *LhrC* in stationary phase (Christiansen et al., 2006), we focused on *LhrA*. *LhrA* was detected in association with intracellular Hfq but remained undetectable in the culture medium (Figure S3C). Collectively, these results strongly indicate that extracellular RNAs complexed with Zea are not originating from lysed bacteria.

(E) Heatmap showing the fold enrichment of phage A118 transcripts in the Zea IP compared to control IP in the bacterial cytosol and culture medium.

(F) RIP-qPCR on RNAs isolated from Zea and control (IgG) immunoprecipitations in the bacterial cytosol (top) and culture medium (bottom). The enrichment of selected phage (*lmo2282* to *lmo2333*) and control genes was calculated after normalization to the corresponding input fractions. Values represent means ± SEM, n = 3. †, not detected. Statistical significance (between the IP IgG and IP αZea) determined by two-tailed t test. See also Figure S2.

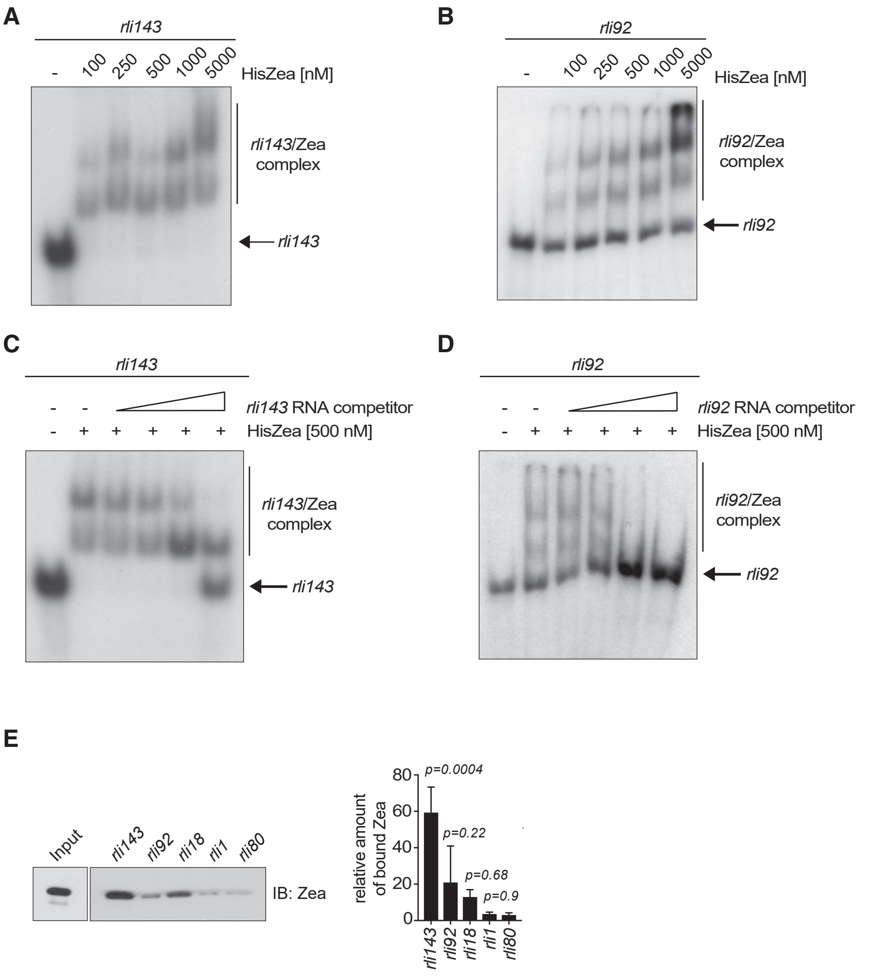

Figure 3. Zea Directly Binds RNA

(A and B) Electrophoretic mobility gel shift assay with *in vitro*-transcribed 5′ end radiolabeled *rli143* (n = 2) (A) and *rli92* (n = 3) (B) in the presence of increasing concentration of HisZea, as indicated.

(C and D) HisZea-*rli143* (n = 2) (C) and HisZea-*rli92* (n = 2) (D) complexes were incubated with increasing concentrations of the corresponding cold competitor RNA.

(E) Immunoblotting (n = 3) of streptavidin affinity pull-down of *in vitro*-transcribed biotinylated transcripts in the presence of HisZea (left); quantification of Zea binding to *rlis* (right). Statistical significance determined by ANOVA with multiple testing against *rli80*.

Zea Overexpression Induces Extracellular Accumulation of Zea-Binding RNAs

Because Zea binds RNA and is also secreted, we sought to determine whether it could affect the amount of RNA in the culture medium. Given the strong binding of Zea to phage RNA (Figures 2C–2F and S2B), we compared the amount of phage RNA present in the culture medium of *L. monocytogenes* wild-type (WT), *Δzea*, and *zea*$^+$. For this purpose, *L. monocytogenes* was grown in minimal medium (MM), because rich medium (BHI) contains RNA. qPCR analysis on RNA extracted from the culture medium revealed that overexpression of Zea increased the amount of the extracellularly detected phage RNA (Figure 4A). The intracellular abundance of the phage transcripts was comparable in the three *L. monocytogenes* strains studied (Figure S4A), indicating that Zea specifically affects the quantity of extracellular RNAs and not their expression level. However, when comparing WT and *Δzea* strains, we did not find remarkable changes in the amount of extracellular phage RNA. This is probably due to the low expression level of Zea by WT bacteria in MM, as revealed by qPCR (Figure S4B). We next evaluated the abundance of another class of highly enriched RNAs specifically bound to Zea: the lma-monocin RNAs (Figure S2B). The lma-monocin locus is considered to be a cryptic prophage whose function remains elusive (Lee et al., 2016; Göhmann et al., 1990). Overexpression of Zea increased the amount of the lma-monocin RNAs in the culture medium (Figure 4B) but not in bacteria (Figure S4C).

This approach could not be applied to secreted small RNAs detected by RIP-seq due to their low expression levels. To overcome this problem, we overexpressed *rli143* in WT, *Δzea*, and *zea*$^+$ *L. monocytogenes* and then measured *rli143* abundance in the culture medium. As a control, we generated a fourth strain overexpressing both *rli143* and Lmo2595, another secreted protein of *L. monocytogenes* (Glaser et al., 2001). In line with the above results, *rli143* accumulated in the culture medium when Zea was co-overexpressed (Figure 4C) but not when Lmo2595 was co-overexpressed. The intracellular expression level of *rli143* was comparable in all of the strains (Figure S4D).

To further establish a role for Zea in the regulation of the extracellular amount of RNA, we generated a *L. innocua* strain overexpressing either *rli143* alone or *rli143* with Zea and then measured the abundance of *rli143* in the culture medium. As a control, *rli143* was co-expressed with Lmo2595. We found that co-expression of Zea and *rli143* induced an even greater accumulation of *rli143* in the culture medium compared with *L. monocytogenes* (Figure 4D). The intrabacterial abundance of *rli143* was comparable in the three *L. innocua* strains (Figure S4E). Altogether, these data show that the overexpression of Zea induces accumulation in the culture medium of phage-derived and small RNAs that are Zea-binding RNA species.

Zea overexpression could increase the amount of extracellular RNA by promoting its export from bacteria and/or by promoting its stabilization in the culture medium. We found that Zea protected *rli143* from RNase-mediated degradation *in vitro* (Figure S5A), indicating that RNA protection may partially account for the increased amount of extracellular RNA.

As a last approach to definitively establish the impact of Zea on secreted RNA, we performed RNA-seq analysis on extracellular RNAs prepared from the WT and *zea*$^+$ strains. We reasoned that if Zea increases the secretion and/or protection of a specific

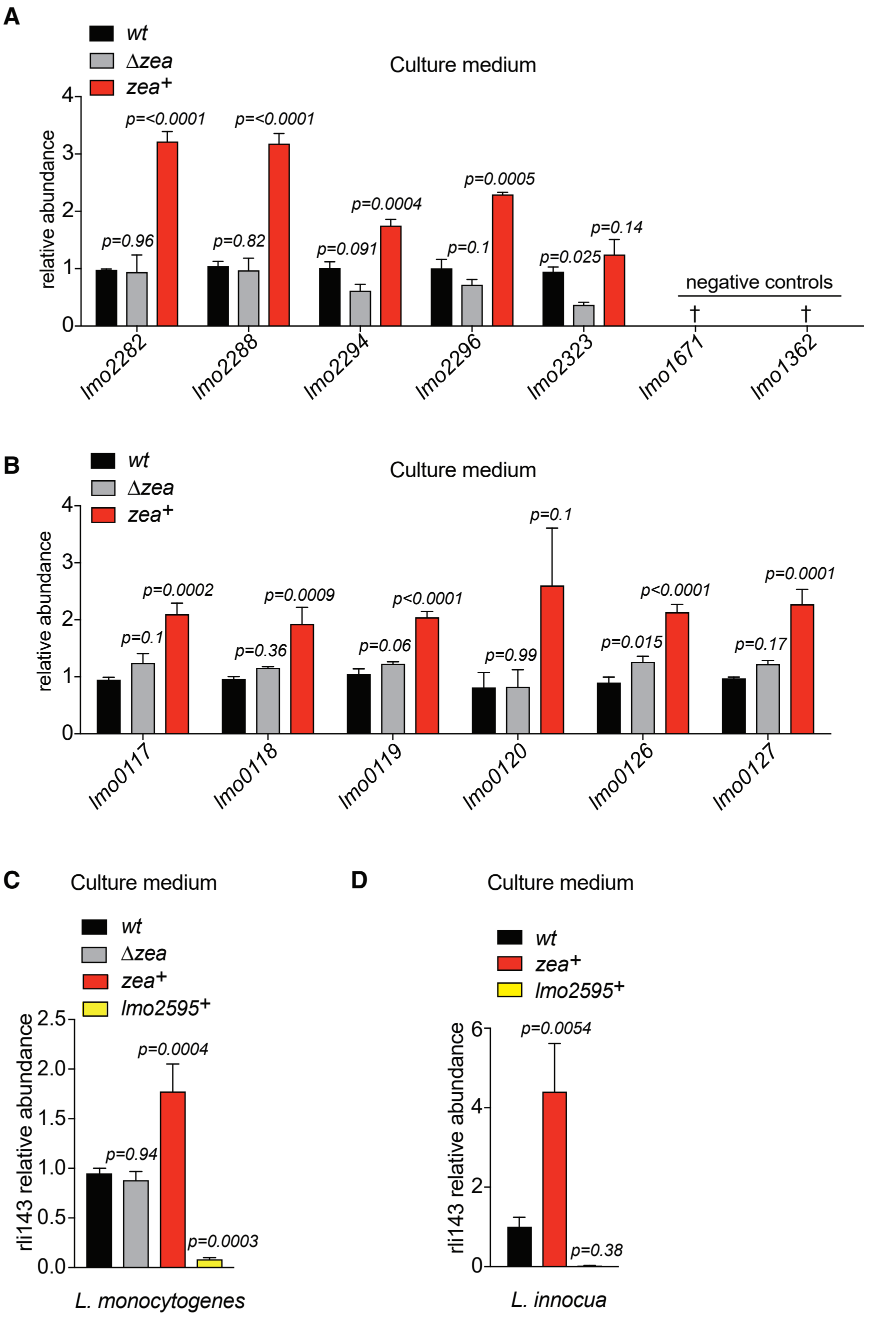

Figure 4. Zea Controls the Abundance of Its Target RNAs in the Culture Medium

(A–D) qPCR analysis on RNA extracted from the culture medium of different *L. monocytogenes* strains (as indicated) for (A) selected phage and control genes, (B) the lma-monocin locus, (C) *rli143* in *L. monocytogenes*, and (D) *rli143* in *L. innocua.* The relative abundance was calculated after normalization to the WT sample. Values represent means ± SEM, n = 3. †, not detected. Statistical significance determined by unpaired ANOVA with multiple testing against WT. See also Figures S4 and S5.

subset of extracellular *L. monocytogenes* RNAs, then its overexpression should increase the overall amount of those RNAs in the medium. We thus purified extracellular RNA from three independent samples (3 from WT and 3 from *zea*$^+$) and performed sequencing. Differential gene expression analysis revealed that, besides the overexpressed Zea RNA, 36 endogenous transcripts were significantly more abundant in the medium of the *zea*$^+$ strain (Figure S5B). The vast majority of these transcripts were mRNAs (78%), while a small percentage represented small non-coding RNAs (sRNAs) and antisense RNAs (10% and 8%, respectively) (Figure S5B). We then examined the correlation between Zea overexpression and the higher amount of extracellular RNA; we intersected the differential extracellular abundance dataset with the dataset of the Zea RIP-seq experiment performed in the culture medium (i.e., the RNAs in complex with Zea). Strikingly, we found that one-third (12 out of 37 RNAs) of the transcripts enriched in the culture medium when Zea was overexpressed were also associated with Zea in the RIP-seq dataset (Figure S5C). This indicates that a subset of transcripts found in complex with Zea becomes more abundant in the medium following Zea overexpression (exact right rank Fisher's test: $p = 7.18 \times 10^{-5}$). Of note, among these 12 enriched secreted RNAs, 8 RNAs proceeded from the A118 phage. qPCR analysis of intrabacterial phage RNA from the WT and *zea*$^+$ strains revealed similar amounts of the majority of the phage genes tested, indicating that Zea does not affect the expression of phage genes (Figure S5D). Altogether, our results show that Zea binds a subset of *L. monocytogenes* RNAs and that its overexpression increases their abundance in the extracellular medium.

Zea Affects *L. monocytogenes* Virulence

The absence of a Zea ortholog in *L. innocua* (Figure 1A) prompted us to assess whether Zea could impact *L. monocytogenes* virulence. We thus examined the properties of the WT and *Δzea* strains in a mouse infection model. After intravenous inoculation, the *Δzea* strain showed a significant increase in bacterial load after 72 h, both in the liver (Figure 5A) and in the spleen (Figure 5B). These results indicate that Zea is an effector that affects *L. monocytogenes* virulence.

Zea Modulates the Type I IFN Response in a RIG-I-Dependent Fashion

Three RBPs of the RIG-I-like receptor (RLR) family (RIG-I, MDA5, and LGP2) can sense non-self RNA in the cytoplasm, but only RIG-I and MDA5 can trigger the type I IFN signaling cascade

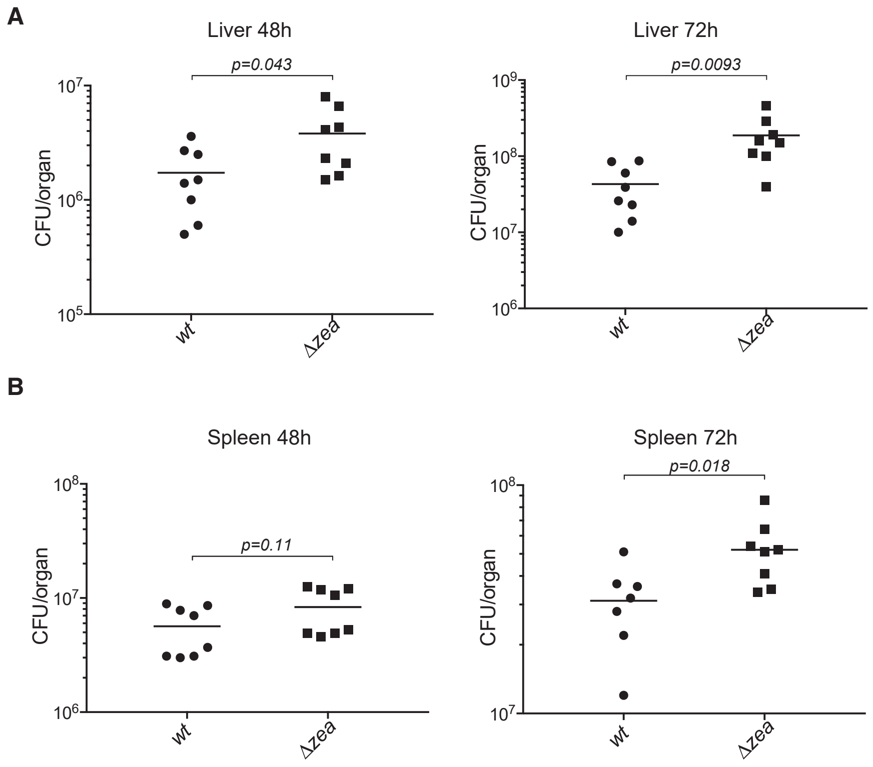

Figure 5. Zea Regulates *L. monocytogenes* Virulence

(A and B) BALB/c mice were inoculated intravenously with *L. monocytogenes* EGD-e (WT) or the *zea*-deleted strain (*Δzea*). After 48 h and 72 h post-infection, livers (A) and spleens (B) were recovered and CFUs assessed by serial dilution and plating. The number of bacteria in each organ is expressed as $\log_{10}$ CFUs. The lines denote the means ± SEM, n = 2. Statistical significance determined by two-tailed t test.

(Chow et al., 2018). A recent approach has successfully helped to identify viral RNA sequences bound to RIG-I, MDA5, or LGP2 during viral infections (Sanchez David et al., 2016; Chazal et al., 2018). This method is based on the affinity purification of stably expressed Strep-tagged RLRs followed by the sequencing of their specific viral RNA partners. We thus applied this approach to obtain *L. monocytogenes*-specific RNAs bound to each of the RLRs upon infection with *L. monocytogenes* WT. We infected HEK293 cells stably expressing Strep-tagged RLRs (or Strep-tagged mCherry as a negative control) with *L. monocytogenes* WT and pulled down the Strep-tagged proteins. Co-purified RNA molecules from three independent replicates were sequenced and mapped to the *L. monocytogenes* genome. We found 15 RNAs specifically enriched in the RIG-I pull-down, and 9 of them (60%) belonged to the phage A118 locus (Figure S6). We did not identify specific RNAs bound to MDA5 and LGP2 (Figure S6), in agreement with a previously suggested major role of RIG-I and minor role of MDA5, in *L. monocytogenes*-induced IFN response infection (Abdullah et al., 2012; Hagmann et al., 2013). These data indicate that during infection, *L. monocytogenes* phage RNAs gain access to the host cytoplasm, where they specifically bind to RIG-I.

Since Zea is a secreted protein and binds phage RNA, we investigated whether it could participate in RIG-I-dependent signaling. We first compared the expression of IFN-β in cells infected with *L. monocytogenes* WT versus cells infected with *zea*$^+$. qPCR analysis revealed that overexpression of Zea increased the amount of IFN-β while IFN-γ was undetectable (Figure 6A). Zea overexpression did not increase the expression of the proinflammatory cytokine interleukin 8 (IL-8). Notably, a *L. monocytogenes* strain overexpressing another secreted protein had no effect on INF-β expression (Figure 6A). Thus, Zea can modulate a type I IFN-β response. Next, to address whether the increased IFN response was mediated by RIG-I, we repeated the same experiment in RIG-I knockdown cells. The Zea-induced IFN-β upregulation was strongly impaired after RIG-I silencing (Figure 6B). These data indicate that Zea plays a role in the RIG-I-dependent type I IFN response.

These findings led us to examine whether Zea and RIG-I might share the same compartment in cells. Attempts to detect endogenous Zea in infected cells by immunoblotting or immunofluorescence were unsuccessful, as our antibodies cross-reacted with some mammalian proteins. We thus infected cells with *zea*^{FLAG} *L. monocytogenes* and used an anti-FLAG antibody for detection. Immunoblotting analysis of cytosolic and nuclear fractions prepared from infected cells revealed that Zea was present in both host cell compartments (Figure S7A), whereas RIG-I is mostly cytosolic (Sánchez-Aparicio et al., 2017; Liu et al., 2018). Transfected FLAG-tagged Zea also localized both to the cytoplasm and the nucleus. (Figure S7B). Thus, the fraction of Zea present in the cytosol might be compatible with the RIG-I-dependent signaling. Next, we tested whether Zea and RIG-I co-localized in cells. FLAG-tagged Zea partially co-localized with endogenous RIG-I, indicating a spatial vicinity of the two proteins (Figure 6C). Of note, a negative control FLAG-tagged mCherry protein did not co-localize with RIG-I (Figure 6C). These results prompted us to test whether Zea could interact with RIG-I. Immunopurified Zea pulled down Strep-tagged RIG-I from cell lysates, indicating interaction between the two proteins (Figure 6D). This interaction did not absolutely require the presence of *L. monocytogenes* RNA, as pre-treatment of immunopurified Zea with RNaseA reduced Zea-RIG-I binding without abolishing it (Figure 6D). In agreement with a minor role of RNA in the Zea-RIG-I interaction, transfection of mammalian cells with FLAG-tagged Zea, which is therefore not bound to *L. monocytogenes* RNA, was able to interact with co-expressed Strep-tagged RIG-I independently of RNA presence (Figure 6E). Altogether, our results show that Zea interacts with RIG-I and modulates RIG-I-dependent type I IFN response.

Since RIG-I activation implies RNA binding, we sought to determine whether Zea-interacting RNAs could trigger an IFN response. We used a reporter-cell line stably transfected with

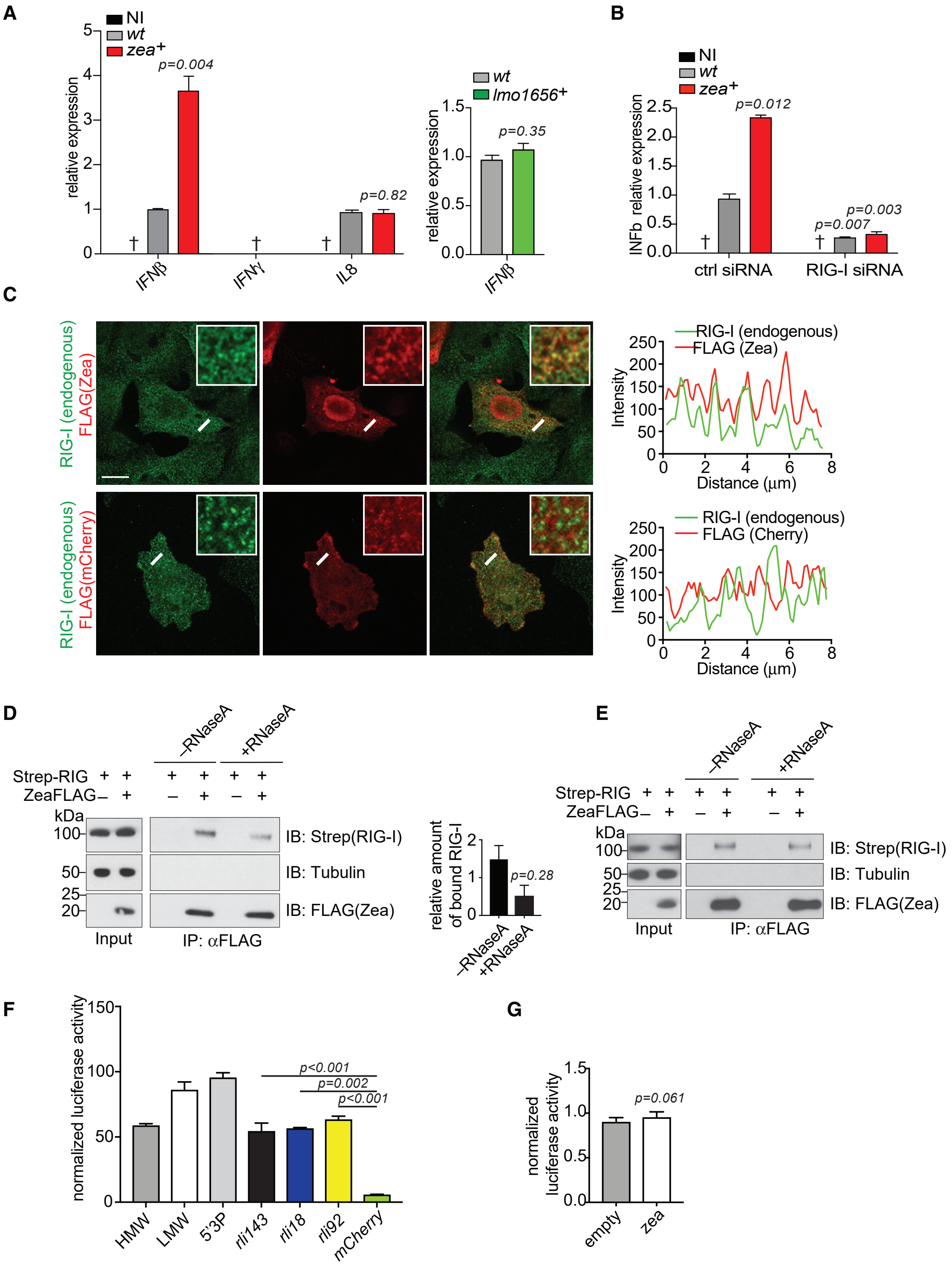

Figure 6. Zea Interacts with RIG-I and Modulates a RIG-I Dependent IFN Response

(A) qPCR analysis of IFN-β, IFN-γ, and interleukin 8 (*IL8*) (n = 3) expression in response to infection with WT and *zea*+ *L. monocytogenes* in LoVo cells (left); qPCR analysis of interleukin 8 (*IL8*) expression in response to infection with WT and *Lmo1656*+ *L. monocytogenes* in LoVo cells infected as above (right). The relative expression was calculated after normalization to (1) the GAPDH as a housekeeping gene and (2) to the WT sample. †, not detected. Statistical significance determined by two-tailed t test.

(legend continued on next page)

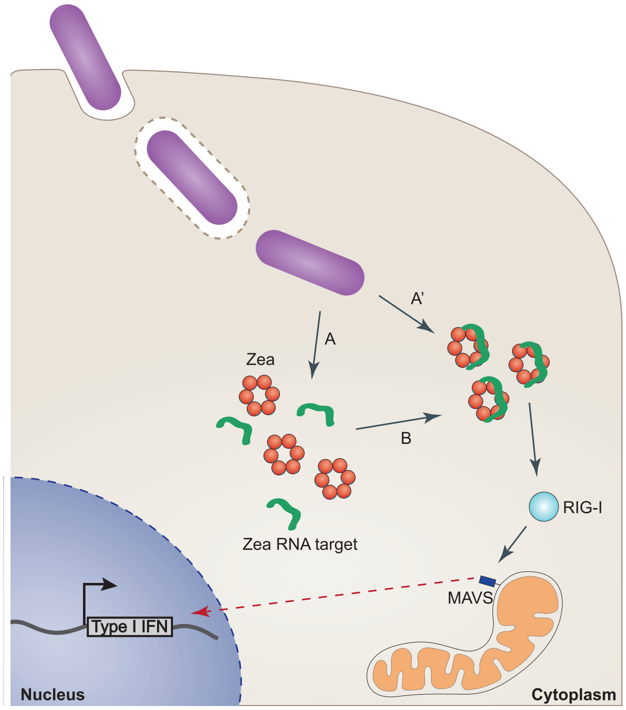

Figure 7. Proposed Model for Zea-Mediated Regulation of IFN Response

(A and B) During infection, *L. monocytogenes* secretes Zea and RNA into (A) the cytoplasm, which then assemble to form (B) a ribonucleoprotein complex. (A′) In alternative, Zea can be directly secreted in an RNA-bound form. Zea-containing ribonucleoprotein complexes then associate to RIG-I, triggering a signaling cascade which would then activate the type I IFN response.

a luciferase gene under the control of a promoter sequence containing five IFN-stimulated response elements (ISREs) (Lucas-Hourani et al., 2013). Transfection of the *in vitro*-transcribed Zea-interacting small RNAs showed strong immunostimulatory activity, while an mCherry control transcript did not (Figure 6F). This suggests that Zea can induce RIG-I activation in infected cells via its associated bacterial RNAs. The expression of Zea protein alone failed to induce any stimulation, indicating that despite its capability to physically interact with RIG-I, Zea cannot promote RIG-I activation by itself (Figure 6G). We conclude that during infection, Zea interacts with RIG-I and modulates RIG-I-dependent signaling. This modulation likely depends on Zea-bound RNA (Figure 7).

DISCUSSION

The major finding of this study is the identification of a secreted RBP from bacteria. We show that *L. monocytogenes* secretes Zea, a small RBP, which associates extracellularly with a subset of *L. monocytogenes* RNAs. We found that the overexpression of Zea correlates with an increased amount of its RNA ligands in the culture medium. This might be due to two non-mutually exclusive phenomena: (1) increased secretion of Zea-RNA complexes and (2) increased stabilization of Zea RNA targets following Zea accumulation in the extracellular medium. In the latter case, the pathway regulating RNA secretion would remain to be identified. The presence of Zea orthologs in other bacterial species indicates that the secretion of RBPs is a conserved phenomenon in prokaryotes. Our results suggest that additional RBPs remain to be identified in bacteria; however, their identification might be difficult, as bacterial RBPs are likely devoid of classical RBDs (Tawk et al., 2017). In agreement with this hypothesis, the analysis of the Zea protein sequence showed that Zea does not possess any recognizable RNA-binding region. Future work will shed light on which amino acids are important for RNA binding as well as whether specific RNA sequences or structures are recognized by Zea.

Our work reveals that the secretion of an RBP during bacterial infection regulates the induction of the INF response. We provide evidence that during *L. monocytogenes* infection, a Zea-containing ribonucleoprotein complex binds to RIG-I and modulates RIG-I-dependent signaling. In agreement with this hypothesis, Zea devoid of its RNA targets has no effect on RIG-I, while Zea-bound RNAs are able to induce an IFN response. In addition, Zea and RIG-I bind a similar subset of *L. monocytogenes* RNAs that is enriched in phage RNAs. We do not exclude that additional factors or signaling events might contribute to the Zea-mediated modulation of RIG-I signaling during *L. monocytogenes* infection. This study provides the identification of the bacterial RNA species recognized by RIG-I during bacterial infection and uncovers that RIG-I binds viral (phage) RNAs from an invading bacterium. These data reinforce the concept that RIG-I has primarily evolved to sense viral RNAs

(B) qPCR analysis of IFN-β expression in response to infection with WT and *zea*$^{+}$ *L. monocytogenes* in LoVo cells transfected with control siRNA (ctrl siRNA) or with RIG-I targeting siRNA (RIG-I siRNA) and infected as above (n = 3). Values represent means ± SEM. Statistical significance determined by two-tailed t test.
(C) Representative confocal images of LoVo cells transfected with FLAG-tagged Zea (top) or FLAG-tagged mCherry (bottom). The co-localization between Zea and RIG-I was assessed with a line scan (white line) whose fluorescence intensity is plotted in red for ZeaFLAG and in green for RIG-I. Top right insets: magnification of the region in which the line scan was performed. Scale bars, 10 μm.
(D) Representative coIP between FLAG-tagged Zea and Strep-tagged RIG-I (left, n = 2). Immunopurified ZeaFLAG treated (+ RNaseA) or not (−RNaseA) with RNase was incubated with a cell lysate from HEK293 cells stably expressing Strep-tagged RIG-I (Sanchez David et al., 2016). Quantification of Zea-bound RIG-I in presence or absence of RNase (right). Statistical significance determined by two-tailed t test.
(E) Representative coIP between FLAG-tagged Zea and Strep-tagged RIG-I (n = 2). LoVo cells were co-transfected with the plasmids encoding FLAG-tagged Zea and Strep-tagged RIG-I and ZeaFLAG was then immunoprecipitated and treated (+ RNaseA) or not (−RNaseA) with RNase before elution with an anti-FLAG peptide.
(F) The immunostimulatory activity of Zea-interacting small RNAs was assessed by transfection into ISRE reporter cells lines (Lucas-Hourani et al., 2013). Values represent means ± SEM, n = 3. Firefly luciferase activity was normalized to mock-transfected cells. *HMW* (high molecular weight), *LMW* (low molecular weight), and *5′3P* (5′ triphosphate-RNA) were used as positive controls. An *mCherry* RNA fragment served as a negative control. Statistical significance determined by two-tailed t test.
(G) The immunostimulatory activity of the Zea protein was assessed by transfection of a Zea-encoding plasmid (*zea*) into the ISRE reporter cells line (Lucas-Hourani et al., 2013). Values represent means ± SEM, n = 3. Statistical significance determined by two-tailed t test. See also Figures S6 and S7.

and highlight that phage RNAs from a bacterial pathogen contribute to RIG-I activation.

Our data show that Zea dampens *L. monocytogenes* virulence *in vivo* as its deletion results in an increased bacteria burden in the organs of infected mice. Strikingly, and in agreement with our results, Zea is absent in the hypervirulent strains of *L. monocytogenes* (lineage I), but it is well conserved in the strains from lineage II, which include a smaller number of clinical isolates. Given the complexity of the innate immune response to *L. monocytogenes* infection (Stockinger et al., 2009; Dussurget et al., 2014; O'Connell et al., 2004; Carrero et al., 2004; Auerbuch et al., 2004), it is difficult to establish the precise role of Zea *in vivo*. We show that Zea participates in the modulation of the IFN response, but we do not exclude that Zea might have additional roles during infection. We found that Zea also localizes to the nucleus of infected cells (Figures 6C and S7), possibly to affect host nuclear functions. It is conceivable that the phenotype observed *in vivo* is a consequence of these additional features of Zea. Notably, Zea orthologs are also present in other bacteria that normally reside in the environment and are rarely associated with disease. In addition to its role in *L. monocytogenes* virulence, Zea might also play a role during the saprophytic life of *L. monocytogenes*. In conclusion, this study revealed that RNA can traffic between different organisms and that an RBP mediates the transfer of this "social RNA," thereby triggering a host response. In line with these findings, a recent paper showed that honeybees secrete an RBP that stabilizes RNA in the environment and facilitates RNA sharing among individuals (Maori et al., 2019). We speculate that secreted RBPs will emerge as new players in host-pathogen crosstalk.

STAR★METHODS

Detailed methods are provided in the online version of this paper and include the following:

- KEY RESOURCES TABLE
- LEAD CONTACT AND MATERIALS AVAILABILITY
- EXPERIMENTAL MODEL AND SUBJECT DETAILS
  - Bacterial Strains and Cell Lines
  - Bacterial Mutant Generation
  - Mice
- METHOD DETAILS
  - Plasmid Vectors and Antibodies
  - Bacterial Fractionation
  - Expression and Purification of HisZea
  - RNA Extraction
  - In Vitro RNA Transcription
  - Electrophoretic Mobility Gel Shift Assay
  - Biotin Pull-Down Assay
  - RNase Protection Assay
  - Immunoprecipitations
  - Immunofluorescence
  - Cell Fractionations
  - Live/Dead Bacterial Staining
  - RIP-Seq
  - RIP-Seq Data Analysis
  - Sequencing of Total Secreted *L. monocytogenes* RNA
  - Sequencing of Secreted *L. monocytogenes* RNA
  - RIP-qPCR
  - Quantitative Real-Time PCRs
  - Purification of RLRs and RNA Extraction
  - Data Analysis of RLR-Associated RNAs
  - Transfection of Zea-Interacting RNAs
  - Mice Infections
- QUANTIFICATION AND STATISTICAL ANALYSIS
- DATA AND CODE AVAILABILITY

SUPPLEMENTAL INFORMATION

Supplemental Information can be found online at https://doi.org/10.1016/j.chom.2019.10.004.

ACKNOWLEDGMENTS

We thank all members of the Cossart laboratory for helpful discussions. We thank Dr. B. Kallipolitis for providing the anti-Listeria Hfq antibody and Dr. E. Hajnsdorf for the anti-*E.coli* Hfq. We acknowledge the platforms of the Grenoble Instruct-ERIC Center (ISBG; UMS 3518 CNRS-CEA-UGA-EMBL) within the Grenoble Partnership for Structural Biology (PSB). Platform access was supported by FRISBI (ANR-10-INBS-05-02) and GRAL, a project of the University Grenoble Alpes graduate school (Ecoles Universitaires de Recherche) CBH-EUR-GS (ANR-17-EURE-0003). The IBS acknowledges integration into the Interdisciplinary Research Institute of Grenoble (IRIG, CEA). We are grateful to R.Y. Sanchez David and R. Viber for technical support. Work in A.L.'s team was supported by Inserm ATIP-Avenir and Mairie de Paris (Programme Émergences—Recherche médicale). This work was supported by grants from the European Research Council (ERC) advanced grant BacCellEpi (670823) and the Fondation Le Roch to P.C.

AUTHOR CONTRIBUTIONS

A.P. designed and conducted most experiments, analyzed data, and wrote the manuscript with inputs from F.S., A.L., and A.V.K.; T.N.T. performed cloning, immunoblotting, and generated several bacterial strains; E.A. performed gel filtration and RIP-qPCRs; S.R. performed qPCRs and pull-downs; B.D. performed EMSA experiments; M.K. performed RLR pull-downs; Q.B. and A.D. purified HisZea and performed gel filtrations; C.B. performed bioinformatics analysis; V.N. performed RLR pull-downs and RNA purifications; M.-A.N. performed animal experiments; F.T. supervised A.V.K.; F.S. performed immunofluorescence experiments; S.B performed initial experiments; C.M. supervised E.A.; A.L. performed qPCRs and provided expertise on data analysis; A.V.K. provided expertise and tools for the RLR part; and P.C. proposed the project to A.P., supervised the project, and edited the manuscript.

DECLARATION OF INTERESTS

The authors declare no competing interests.

Received: May 24, 2019
Revised: August 21, 2019
Accepted: October 7, 2019
Published: November 21, 2019

REFERENCES

Abdullah, Z., Schlee, M., Roth, S., Mraheil, M.A., Barchet, W., Böttcher, J., Hain, T., Geiger, S., Hayakawa, Y., Fritz, J.H., et al. (2012). RIG-I detects infection with live Listeria by sensing secreted bacterial nucleic acids. EMBO J. *31*, 4153–4164.

Allemand, E., Myers, M.P., Garcia-Bernardo, J., Harel-Bellan, A., Krainer, A.R., and Muchardt, C. (2016). A Broad Set of Chromatin Factors Influences Splicing. PLoS Genet. *12*, e1006318.

Anders, S., Pyl, P.T., and Huber, W. (2015). HTSeq–a Python framework to work with high-throughput sequencing data. Bioinformatics *31*, 166–169.

Antson, A.A., Otridge, J., Brzozowski, A.M., Dodson, E.J., Dodson, G.G., Wilson, K.S., Smith, T.M., Yang, M., Kurecki, T., and Gollnick, P. (1995). The structure of trp RNA-binding attenuation protein. Nature *374*, 693–700.

Archambaud, C., Gouin, E., Pizarro-Cerda, J., Cossart, P., and Dussurget, O. (2005). Translation elongation factor EF-Tu is a target for Stp, a serine-threonine phosphatase involved in virulence of Listeria monocytogenes. Mol. Microbiol. *56*, 383–396.

Arnaud, M., Chastanet, A., and Débarbouillé, M. (2004). New vector for efficient allelic replacement in naturally nontransformable, low-GC-content, gram-positive bacteria. Appl. Environ. Microbiol. *70*, 6887–6891.

Arroyo, J.D., Chevillet, J.R., Kroh, E.M., Ruf, I.K., Pritchard, C.C., Gibson, D.F., Mitchell, P.S., Bennett, C.F., Pogosova-Agadjanyan, E.L., Stirewalt, D.L., et al. (2011). Argonaute2 complexes carry a population of circulating microRNAs independent of vesicles in human plasma. Proc. Natl. Acad. Sci. USA *108*, 5003–5008.

Auerbuch, V., Brockstedt, D.G., Meyer-Morse, N., O'Riordan, M., and Portnoy, D.A. (2004). Mice lacking the type I interferon receptor are resistant to Listeria monocytogenes. J. Exp. Med. *200*, 527–533.

Babitzke, P., Bear, D.G., and Yanofsky, C. (1995). TRAP, the trp RNA-binding attenuation protein of Bacillus subtilis, is a toroid-shaped molecule that binds transcripts containing GAG or UAG repeats separated by two nucleotides. Proc. Natl. Acad. Sci. USA *92*, 7916–7920.

Balestrino, D., Hamon, M.A., Dortet, L., Nahori, M.A., Pizarro-Cerda, J., Alignani, D., Dussurget, O., Cossart, P., and Toledo-Arana, A. (2010). Single-cell techniques using chromosomally tagged fluorescent bacteria to study Listeria monocytogenes infection processes. Appl. Environ. Microbiol. *76*, 3625–3636.

Batsché, E., Yaniv, M., and Muchardt, C. (2006). The human SWI/SNF subunit Brm is a regulator of alternative splicing. Nat. Struct. Mol. Biol. *13*, 22–29.

Bécavin, C., Koutero, M., Tchitchek, N., Cerutti, F., Lechat, P., Maillet, N., Hoede, C., Chiapello, H., Gaspin, C., and Cossart, P. (2017). Listeriomics: an Interactive Web Platform for Systems Biology of *Listeria*. mSystems *2*, e00186-16.

Carrero, J.A., Calderon, B., and Unanue, E.R. (2004). Type I interferon sensitizes lymphocytes to apoptosis and reduces resistance to Listeria infection. J. Exp. Med. *200*, 535–540.

Chazal, M., Beauclair, G., Gracias, S., Najburg, V., Simon-Lorière, E., Tangy, F., Komarova, A.V., and Jouvenet, N. (2018). RIG-I Recognizes the 5′ Region of Dengue and Zika Virus Genomes. Cell Rep. *24*, 320–328.

Chevalier, C., Geissmann, T., Helfer, A.C., and Romby, P. (2009). Probing mRNA structure and sRNA-mRNA interactions in bacteria using enzymes and lead(II). Methods Mol. Biol. *540*, 215–232.

Chow, K.T., Gale, M., Jr., and Loo, Y.M. (2018). RIG-I and Other RNA Sensors in Antiviral Immunity. Annu. Rev. Immunol. *36*, 667–694.

Christiansen, J.K., Nielsen, J.S., Ebersbach, T., Valentin-Hansen, P., Søgaard-Andersen, L., and Kallipolitis, B.H. (2006). Identification of small Hfq-binding RNAs in Listeria monocytogenes. RNA *12*, 1383–1396.

Criscuolo, A., and Brisse, S. (2013). AlienTrimmer: a tool to quickly and accurately trim off multiple short contaminant sequences from high-throughput sequencing reads. Genomics *102*, 500–506.

David, D.J., Pagliuso, A., Radoshevich, L., Nahori, M.A., and Cossart, P. (2018). Lmo1656 is a secreted virulence factor of *Listeria monocytogenes* that interacts with the sorting nexin 6-BAR complex. J. Biol. Chem. *293*, 9265–9276.

Dorscht, J., Klumpp, J., Bielmann, R., Schmelcher, M., Born, Y., Zimmer, M., Calendar, R., and Loessner, M.J. (2009). Comparative genome analysis of Listeria bacteriophages reveals extensive mosaicism, programmed translational frameshifting, and a novel prophage insertion site. J. Bacteriol. *191*, 7206–7215.

Dussurget, O., Bierne, H., and Cossart, P. (2014). The bacterial pathogen Listeria monocytogenes and the interferon family: type I, type II and type III interferons. Front. Cell. Infect. Microbiol. *4*, 50.

Ewels, P., Magnusson, M., Lundin, S., and Käller, M. (2016). MultiQC: summarize analysis results for multiple tools and samples in a single report. Bioinformatics *32*, 3047–3048.

Glaser, P., Frangeul, L., Buchrieser, C., Rusniok, C., Amend, A., Baquero, F., Berche, P., Bloecker, H., Brandt, P., Chakraborty, T., et al. (2001). Comparative genomics of Listeria species. Science *294*, 849–852.

Göhmann, S., Leimeister-Wächter, M., Schiltz, E., Goebel, W., and Chakraborty, T. (1990). Characterization of a Listeria monocytogenes-specific protein capable of inducing delayed hypersensitivity in Listeria-immune mice. Mol. Microbiol. *4*, 1091–1099.

Graves, L.M., Helsel, L.O., Steigerwalt, A.G., Morey, R.E., Daneshvar, M.I., Roof, S.E., Orsi, R.H., Fortes, E.D., Milillo, S.R., den Bakker, H.C., et al. (2010). Listeria marthii sp. nov., isolated from the natural environment, Finger Lakes National Forest. Int. J. Syst. Evol. Microbiol. *60*, 1280–1288.

Hagmann, C.A., Herzner, A.M., Abdullah, Z., Zillinger, T., Jakobs, C., Schuberth, C., Coch, C., Higgins, P.G., Wisplinghoff, H., Barchet, W., et al. (2013). RIG-I detects triphosphorylated RNA of Listeria monocytogenes during infection in non-immune cells. PLoS One *8*, e62872.

Holmqvist, E., and Vogel, J. (2018). RNA-binding proteins in bacteria. Nat. Rev. Microbiol. *16*, 601–615.

Langmead, B., and Salzberg, S.L. (2012). Fast gapped-read alignment with Bowtie 2. Nat. Methods *9*, 357–359.

Lee, H.H., Kim, H.S., Kang, J.Y., Lee, B.I., Ha, J.Y., Yoon, H.J., Lim, S.O., Jung, G., and Suh, S.W. (2007). Crystal structure of human nucleophosmin-core reveals plasticity of the pentamer-pentamer interface. Proteins *69*, 672–678.

Lee, G., Chakraborty, U., Gebhart, D., Govoni, G.R., Zhou, Z.H., and Scholl, D. (2016). F-Type Bacteriocins of Listeria monocytogenes: a New Class of Phage Tail-Like Structures Reveals Broad Parallel Coevolution between Tailed Bacteriophages and High-Molecular-Weight Bacteriocins. J. Bacteriol. *198*, 2784–2793.

Li, H., Handsaker, B., Wysoker, A., Fennell, T., Ruan, J., Homer, N., Marth, G., Abecasis, G., and Durbin, R.; 1000 Genome Project Data Processing Subgroup (2009). The Sequence Alignment/Map format and SAMtools. Bioinformatics *25*, 2078–2079.

Liao, Y., Smyth, G.K., and Shi, W. (2014). featureCounts: an efficient general purpose program for assigning sequence reads to genomic features. Bioinformatics *30*, 923–930.

Liu, G., Lu, Y., Thulasi Raman, S.N., Xu, F., Wu, Q., Li, Z., Brownlie, R., Liu, Q., and Zhou, Y. (2018). Nuclear-resident RIG-I senses viral replication inducing antiviral immunity. Nat. Commun. *9*, 3199.

Love, M.I., Huber, W., and Anders, S. (2014). Moderated estimation of fold change and dispersion for RNA-seq data with DESeq2. Genome Biol. *15*, 550.

Lucas-Hourani, M., Dauzonne, D., Jorda, P., Cousin, G., Lupan, A., Helynck, O., Caignard, G., Janvier, G., André-Leroux, G., Khiar, S., et al. (2013). Inhibition of pyrimidine biosynthesis pathway suppresses viral growth through innate immunity. PLoS Pathog. *9*, e1003678.

Maori, E., Navarro, I.C., Boncristiani, H., Seilly, D.J., Rudolph, K.L.M., Sapetschnig, A., Lin, C.C., Ladbury, J.E., Evans, J.D., Heeney, J.L., and Miska, E.A. (2019). A Secreted RNA Binding Protein Forms RNA-Stabilizing Granules in the Honeybee Royal Jelly. Mol. Cell *74*, 598–608.e6.

Mellin, J.R., Tiensuu, T., Bécavin, C., Gouin, E., Johansson, J., and Cossart, P. (2013). A riboswitch-regulated antisense RNA in Listeria monocytogenes. Proc. Natl. Acad. Sci. USA *110*, 13132–13137.

O'Connell, R.M., Saha, S.K., Vaidya, S.A., Bruhn, K.W., Miranda, G.A., Zarnegar, B., Perry, A.K., Nguyen, B.O., Lane, T.F., Taniguchi, T., et al. (2004). Type I interferon production enhances susceptibility to Listeria monocytogenes infection. J. Exp. Med. *200*, 437–445.

Pereira, J.M., Chevalier, C., Chaze, T., Gianetto, Q., Impens, F., Matondo, M., Cossart, P., and Hamon, M.A. (2018). Infection Reveals a Modification of SIRT2 Critical for Chromatin Association. Cell Rep. *23*, 1124–1137.

Prokop, A., Gouin, E., Villiers, V., Nahori, M.A., Vincentelli, R., Duval, M., Cossart, P., and Dussurget, O. (2017). OrfX, a Nucleomodulin Required for *Listeria monocytogenes* Virulence. MBio *8*, e01550-17.

Ramírez, F., Ryan, D.P., Grüning, B., Bhardwaj, V., Kilpert, F., Richter, A.S., Heyne, S., Dündar, F., and Manke, T. (2016). deepTools2: a next generation web server for deep-sequencing data analysis. Nucleic Acids Res. *44* (W1), W160-5.

Robinson, M.D., McCarthy, D.J., and Smyth, G.K. (2010). edgeR: a Bioconductor package for differential expression analysis of digital gene expression data. Bioinformatics *26*, 139–140.

Sánchez-Aparicio, M.T., Ayllón, J., Leo-Macias, A., Wolff, T., and García-Sastre, A. (2017). Subcellular Localizations of RIG-I, TRIM25, and MAVS Complexes. J. Virol. *91*, e01155-16.

Sanchez David, R.Y., Combredet, C., Sismeiro, O., Dillies, M.A., Jagla, B., Coppée, J.Y., Mura, M., Guerbois Galla, M., Despres, P., Tangy, F., and Komarova, A.V. (2016). Comparative analysis of viral RNA signatures on different RIG-I-like receptors. eLife *5*, e11275.

Shurtleff, M.J., Temoche-Diaz, M.M., Karfilis, K.V., Ri, S., and Schekman, R. (2016). Y-box protein 1 is required to sort microRNAs into exosomes in cells and in a cell-free reaction. eLife *5*, e19276.

Stockinger, S., Kastner, R., Kernbauer, E., Pilz, A., Westermayer, S., Reutterer, B., Soulat, D., Stengl, G., Vogl, C., Frenz, T., et al. (2009). Characterization of the interferon-producing cell in mice infected with Listeria monocytogenes. PLoS Pathog. *5*, e1000355.

Tawk, C., Sharan, M., Eulalio, A., and Vogel, J. (2017). A systematic analysis of the RNA-targeting potential of secreted bacterial effector proteins. Sci. Rep. *7*, 9328.

Thomsen, N.D., and Berger, J.M. (2009). Running in reverse: the structural basis for translocation polarity in hexameric helicases. Cell *139*, 523–534.

Trieu-Cuot, P., Carlier, C., Poyart-Salmeron, C., and Courvalin, P. (1991). Shuttle vectors containing a multiple cloning site and a lacZ alpha gene for conjugal transfer of DNA from Escherichia coli to gram-positive bacteria. Gene *102*, 99–104.

Varet, H., Brillet-Guéguen, L., Coppée, J.Y., and Dillies, M.A. (2016). SARTools: A DESeq2- and EdgeR-Based R Pipeline for Comprehensive Differential Analysis of RNA-Seq Data. PLoS One *11*, e0157022.

Vickers, K.C., Palmisano, B.T., Shoucri, B.M., Shamburek, R.D., and Remaley, A.T. (2011). MicroRNAs are transported in plasma and delivered to recipient cells by high-density lipoproteins. Nat. Cell Biol. *13*, 423–433.

Vogel, J., and Luisi, B.F. (2011). Hfq and its constellation of RNA. Nat. Rev. Microbiol. *9*, 578–589.

Wang, K., Zhang, S., Weber, J., Baxter, D., and Galas, D.J. (2010). Export of microRNAs and microRNA-protective protein by mammalian cells. Nucleic Acids Res. *38*, 7248–7259.

Wu, T.D., Reeder, J., Lawrence, M., Becker, G., and Brauer, M.J. (2016). GMAP and GSNAP for Genomic Sequence Alignment: Enhancements to Speed, Accuracy, and Functionality. Methods Mol. Biol. *1418*, 283–334.

Wurtzel, O., Sesto, N., Mellin, J.R., Karunker, I., Edelheit, S., Bécavin, C., Archambaud, C., Cossart, P., and Sorek, R. (2012). Comparative transcriptomics of pathogenic and non-pathogenic Listeria species. Mol. Syst. Biol. *8*, 583.

STAR★METHODS

KEY RESOURCES TABLE

REAGENT or RESOURCE	SOURCE	IDENTIFIER
**Antibodies**		
Rabbit anti-Lmo2686	This study	N/A
Rabbit anti-EF-Tu	Prokop et al., 2017	N/A
Rabbit anti-InlC	Prokop et al., 2017	N/A
Mouse anti-Flag M2	Sigma	Cat#F3165; RRID: AB_259529
Rabbit anti-HA	AbCam	Cat#Ab137838; RRID: AB_2810986
Rabbit anti-Hfq	Christiansen et al., 2006	N/A
Mouse anti-Strep tag	QIAGEN	Cat#34850; RRID: AB_2810987
Mouse anti-RIG-I	Millipore	Cat#MABF297; RRID: AB_2650546
Mouse anti-Tubulin	Sigma	Cat#T9026; RRID: AB_477593
Mouse anti-H3	Cell Signaling	Cat#3638; RRID: AB_1642229
**Bacterial and Virus Strains**		
L. monocytogenes EGD-e (wt strain)	Pasteur Institute	N/A
L. monocytogenes *Δzea* (*zea* deleted strain)	This study	N/A
L. monocytogenes Δzea+zea^{+} (zea overexpressing strain in *Δzea* background)	This study	N/A
L. monocytogenes zea^{+} (zea overexpressing strain in wt background)	This study	N/A
L. monocytogenes zea^{Flag} +Zea^{HA}-*pP1* (zea^{Flag} and Zea^{HA}overexpressing strain in wt background)	This study	N/A
L. monocytogenes zea^{Flag} (zea^{Flag} overexpressing strain in wt background)	This study	N/A
L. monocytogenes zea^{HA} (zea^{HA} overexpressing strain in wt background)	This study	N/A
L. monocytogenes *rli143* (rli143 overexpressing strain in wt background)	This study	N/A
L. monocytogenes *rli143*+zea^{+} (rli143 and zea overexpressing strain in wt background)	This study	N/A
L. monocytogenes *rli143*+$lmo2595^{+}$ (rli143 and lmo2595 overexpressing strain in wt background)	This study	N/A
L. innocua *rli143* (rli143 overexpressing strain in wt background)	This study	N/A
L. innocua *rli143*+zea^{+} (rli143 and zea overexpressing strain in wt background)	This study	N/A
L. innocua *rli143* +$lmo595^{+}$ (rli143 and lmo2595 overexpressing strain in wt background)	This study	N/A
**Chemicals, Peptides, and Recombinant Proteins**		
Brain Heart Infusion medium	GIBCO	Cat#237500
Terrific broth	GIBCO	Cat#A1374301
Ham's F-12K medium	GIBCO	Cat#21127-022
DMEM	GIBCO	Cat#61965-026
Glutamax	GIBCO	Cat#35050-038

(*Continued on next page*)

Continued

REAGENT or RESOURCE	SOURCE	IDENTIFIER
Penicillin-Streptomycin	GIBCO	Cat#15140-122
G418	GIBCO	Cat#10131-027
Lipofectamine LTX	Invitrogen	Cat#155338-100
Lipofectamine 2000	Invitrogen	Cat#11668-019
NuPage LDS sample buffer	Invitrogen	Cat#NP0007
cOmplete protease inhibitor	Roche	Cat#11697498001
NuPage 4%-12% Bis-Tris gel	Life Technologies	Cat#NP0335BOX
Novex TBE 8% gel	Thermo Fisher	Cat#EC62155BOX
Novex TBE 6% gel	Thermo Fisher	Cat#EC6865BOX
Lysing matrix tubes	MP Biomedicals	Cat#6911-500
Amicon Ultra (centrifugal filters) 3K	Millipore	Cat#UFC800324
Amicon Ultra (centrifugal filters) 10K	Millipore	Cat#UFC901024
Streptavidin magnetic beads	New England Biolabs	Cat#S1420S
Flag M2 magnetic beads	Sigma	Cat#M8823-1ML
Normal rabbit IgG	Cell Signaling	Cat#0712017
Protein A Sepharose beads	GE Healtcare	Cat#17-0780-01
Protein A dynabeads	Invitrogen	Cat#10002D
Streptactin Sepharose	GE Healtcare	Cat#28-9355-99
NiNTA agarose beads	QIAGEN	Cat#30210
RNase A	Thermo Scientific	Cat#EN0531
RNasein	Promega	Cat#N2518
Turbo DNase	Invitrogen	Cat#AM2238
Proteinase K	Roche	Cat#03115828001
3xFlag peptide	Sigma	Cat#F4799
Thrichloroacetic acid	Sigma	Cat#T0699
Acetone	Sigma	Cat#200-662-2
Chloroform	Sigma	Cat#288306
Isopropanol	Sigma	Cat#59300
Nuclease-free water	Ambion	Cat#AM9932
TRIzol	Ambion	Cat#15596018
Tri reagent LS	Sigma	Cat#T3934
Acid Phenol	Sigma	Cat#P4682
Sodium Acetate	Ambion	Cat#AM9740
Glycogen	Invitrogen	Cat#AM9510
Fragmentation reagents	Thermo Scientific	Cat#AM8740
Biotin 16 UTP	Invitrogen	Cat#AM8452
Yeast tRNA	Invitrogen	Cat#AM7119
Clean Cap OVAmRNA	TriLink	Cat#TD-OB07A
BSA fatty acid-free	Sigma	Cat#A6003
Passive lysis buffer	Promega	Cat#E1941
Critical Commercial Assays		
RNA 6000 Nano assay	Agilent	Cat#5067-1511
RNA 6000 Pico assay	Agilent	Cat#5067-1513
NEBNext Multiplex Small RNA Library Prep Set for Illumina (Set 1)	NEB	Cat#E7300
Maxiscript™ T7 Transcription Kit	Invitrogen	Cat#AM1312
Gel Extraction kit	QIAGEN	Cat#28115
Brillant III Ultra Fast SYBR-Green QPCR Master Mix	Agilent	Cat#600882
DNA-*free*™ DNA Removal Kit	Invitrogen	Cat#AM1906

(*Continued on next page*)

Continued

REAGENT or RESOURCE	SOURCE	IDENTIFIER
Ribo-Zero rRNA Removal Kit	Illumina	Cat#MRZMB126
QuantiTect Reverse Transcription Kit	QIAGEN	Cat#205311
Bright-Glo Luciferase Assay System	Promega	Cat#E2650
LIVE/DEAD *Bac*Light Bacterial Viability Kit	Thermo Fisher	L7012
Deposited Data		
RIP-Seq	This paper	EMBL-EBI https://www.ebi.ac.uk/arrayexpress; ArrayExpress: E-MTAB-7665
RNA Seq	This paper	EMBL-EBI https://www.ebi.ac.uk/arrayexpress; ArrayExpress: E-MTAB-7665
RLRs purification and sequencing	This paper	EMBL-EBI https://www.ebi.ac.uk/arrayexpress; ArrayExpress: E-MTAB-7665
RIP-Seq, RNA Seq, RLRs purification and sequencing analysis	This paper	https://github.com/becavin-lab/RIPSeq-Listeria
Experimental Models: Cell Lines		
LoVo cells	ATCC	CCL-229
HEK293 Strep-mCherry	Sanchez David et al., 2016	N/A
HEK293 Strep-RIG-I	Sanchez David et al., 2016	N/A
HEK293 Strep-MDA5	Sanchez David et al., 2016	N/A
HEK293 Strep-LGP2	Sanchez David et al., 2016	N/A
STING-37	Lucas-Hourani et al., 2013	N/A
Experimental Models: Organisms/Strains		
Mouse: BALB/c	Charles River	028
Oligonucleotides		
For more oligonucleotides, see Table S2	N/A	N/A
Zea fw (for pAD cloning) GAGTCACGGCCGATAAAGCAAGCATATAATA	This study	N/A
Zeaflag rv (for pAD cloning) ACGTGTCGACTTActtgtcatcgtcgtccttgtagtcTTTAAAACTTGTAGTTAACTTTTTCCCGCT	This study	N/A
Zea no tag rv (for pAD cloning) ACGTGTCGACTTATTTAAAACTTGTAGTTAACTTTTTCCCGCT	This study	N/A
Lmo2595 fw (for pAD cloning) GAGTCACGGCCGATAAAGCAAGCATATAATA	This study	N/A
Lmo2595 rv (for pAD cloning) ACGTGTCGACTTATTTAAAACTTGTAGTTAACTTTTTCCCGCT	This study	N/A
ZeaHA fw (for pP18 cloning) GCAGGGATCCATGAAGGAATTTTTATTTTTTGCTGTATTTACT	This study	N/A
ZeaHA rw (for pP1 cloning) ACGTGTCGACTTACGCGTAATCAGGCACATCATACGGGTATTTAAAACTTGTAGTTAACTTTTTCCCGCT	This study	N/A
Zea fw (for pet28a cloning) CGCGGGATCCATGAAGGAATTTTTATTTTTTGC	This study	N/A
ZeaHis rv (for pet28a cloning) CGCGCTCGAGTTATTTAAAACTTGTAGTTAACTTTTTCCCGC	This study	N/A
Lmo2282 qPCR fw CAAATATTGAACCTTCAATAATCGAAAACGGC	This study	N/A

(*Continued on next page*)

Continued		
REAGENT or RESOURCE	SOURCE	IDENTIFIER
Software and Algorithms		
AlienTrimmer 0.4.0	Criscuolo and Brisse, 2013	ftp://ftp.pasteur.fr/pub/gensoft/projects/AlienTrimmer/
Bowtie2 2.1.0	Langmead and Salzberg, 2012	https://sourceforge.net/projects/bowtie-bio/files/bowtie2/2.2.1/
SAMtools 0.1.19	Li et al., 2009	https://sourceforge.net/projects/samtools/files/samtools/0.1.19/
FastQC 0.10.1	Ewels et al., 2016	https://github.com/s-andrews/FastQC/releases
MultiQC 0.7	Ewels et al., 2016	https://multiqc.info/docs/
FeatureCount	Liao et al., 2014	https://sourceforge.net/projects/subread/files/subread-1.4.6-p3/
SARTools	Varet et al., 2016	https://github.com/PF2-pasteur-fr/SARTools
TMM (edgeR package)	Robinson et al., 2010	https://bioconductor.org/packages/release/bioc/html/edgeR.html
GSNAP (v2018-07-04)	Wu et al., 2016	http://research-pub.gene.com/gmap/src/gmap-gsnap-2018-07-04.tar.gz
HTSeq 0.9.1	Anders et al., 2015	https://htseq.readthedocs.io/en/release_0.11.1/install.html
deepTools 3.1.3	Ramírez et al., 2016	https://deeptools.readthedocs.io/en/develop/content/installation.html

LEAD CONTACT AND MATERIALS AVAILABILITY

Further information and requests for resources and reagents should be directed to and will be fulfilled by the Lead Contact, Pascale Cossart (pcossart@pasteur.fr).

EXPERIMENTAL MODEL AND SUBJECT DETAILS

Bacterial Strains and Cell Lines

L. monocytogenes EGD-e strain was used as the parental strain (detailed informations on the strains used in this study are provided in the Key Resources Table). *L. monocytogenes* strains were grown in brain heart infusion (BHI) medium (GIBCO) with shaking at 200 rpm at 37°C. *E. coli* cells were grown in LB broth. When required, antibiotics were added (chloramphenicol at 35 μg/mL for *E. coli* or 7 μg/mL for *L. monocytogenes,* erythromycin 5 μg/mL for *L. monocytogenes*). LoVo cells were maintained in Ham's F-12K medium (GIBCO) supplemented with 20% fetal calf serum and Glutamax (GIBCO). Strep-tagged RIG-I, MDA5, LGP2 and mCherry cell lines (Sánchez-Aparicio et al., 2017) were maintained in Dulbecco's modified Eagle medium (GIBCO) supplemented with 10% heat-inactivated fetal calf serum (GE Healthcare) and 10,000 U/mL of Penicillin-Streptomycin (Life Technologies) and G418 (Sigma) at 500 μg/mL. The ISRE reporter cell line (STING-37) corresponding to HEK293 cells stably transfected with an ISRE-luciferase reporter-gene was previously described (Lucas-Hourani et al., 2013). All cell lines were maintained and propagated at 37°C with 10% CO_2.

Bacterial Mutant Generation

For the deletion of *zea*, PCR products comprising ~500 bp upstream and downstream of the zea open reading frame (ORF) were fused via splicing by overlap extension PCR and cloned with appropriate restriction sites into the integrative suicide vector pMAD as previously described (Arnaud et al., 2004).

Mice

BALB/c mice (8-week-old female) were purchased by Charles River, Inc. All animal experiments were carried out in strict accordance with the French national and European laws and conformed to the Council Directive on the approximation of laws, regulations, and administrative provisions of the Member States regarding the protection of animals used for experimental and other scientific purposes (86/609/Eec). Experiments that relied on laboratory animals were performed in strict accordance with the Institut Pasteur's regulations for animal care and use protocol, which was approved by the Animal Experiment Committee of the Institut Pasteur (approval no. 03-49).

METHOD DETAILS

Plasmid Vectors and Antibodies

Information about the oligonucleotides used for cloning are provided in the Key Resources Table. To create the plasmids for the overexpression in *L. monocytogenes* of both full-length ZeaFlag, full-length untagged Zea and Lmo2595, the entire ORFs (with or without a Flag tag at the C terminus for Zea) were synthesized as a gBlocks (Integrated DNA Technologies) and subcloned into the integrative plasmid pAD, downsteam of the *Phyper* promoter (Balestrino et al., 2010). The same strategy was used to generate a plasmid overexpressing ZeaHA (C-terminal HA-tag), but the cloning was subsequently performed in the pP1 plasmid [pAT18 derivative (Trieu-Cuot et al., 1991)]. To create the plasmid for the overexpression of rli143, a fusion fragment corresponding to the entire rli143 gene downstream the *pHyper* promoter was synthesized as a gBlock (Integrated DNA Technologies) and cloned in the pP1 plasmid with the appropriate restriction enzymes. To create a plasmid for the overexpression of HisZea in *E. coli* (N-terminal His-tag), the *zea ORF* was amplified by PCR from *L. monocytogenes* genomic DNA and cloned with the appropriate restriction enzymes into the pET28a plasmid, downstream of the polyhistidine tag. To create a plasmid for the overexpression of ZeaFlag in mammalian cells, the cDNA encoding the predicted mature form of Zea was codon-optimized for human expression and synthesized (GeneCust) with a 2xFlag tag at the N terminus. The resulting construct was then subcloned into pcDNA3.1(+) using the appropriate restriction sites. Modified pCineo plasmid carrying GW cassette (pCineoGW) and the Cherry coding sequence was provided by Dr.Yves Jacob (Institut Pasteur). pEXPR-IBA105-RIG-I and pEXPR-IBA105-mCherry plasmids for the overexpression of Strep-RIG-I and Strep-mCherry, respectively, were previously described (Sanchez David et al., 2016).

Anti-Zea polyclonal antibodies were raised against three synthetic peptides spanning the C terminus of the protein (CSFNAKINVSKGKGKITS; FYSPGLDVKKSKLSKTS; TLKASVSGKKLTTSFK). Two rabbits were injected with each antigen supplemented with Freund's adjuvant (Covalabs, Villeurbanne, France). The total IgG fractions were affinity-purified via a resin column containing the antigenic peptide. The affinity-purified antibodies were dialyzed against PBS and 50% glycerol and stored at -20°C. A mix of the three antibodies (1 μg/ mL in total) was used for immunoblotting. The specificity of the anti-Zea antibodies in immunoblotting and immunoprecipitation was verified by comparing bacterial extract and culture medium prepared from the zea^+ and the *Δzea* strains.

Bacterial Fractionation

For detection of endogenous Zea in culture medium, *L. monocytogenes* was grown to exponential phase (OD_{600} = 1). Bacteria were harvested by centrifugation (4000 × g, 30 min, 4°C) and proteins in the culture medium fraction were precipitated by addition of 40% ammonium sulfate and incubated at 4°C (overnight, gentle shaking). Protein were recovered by centrifugation (30 min, 16,000 × *g*, 4°C) and resuspended in water. Samples were dialyzed against water (overnight, 4°C), concentrated using an Amicon centrifugal filter units (3K cut-off, Millipore) and resuspended in LDS-PAGE sample loading buffer (NuPage, Life Technologies). The bacterial pellet was washed twice in PBS and resuspended in lysis buffer [20mM Tris pH 8.0, 1 mM $MgCl_2$, 150 mM KCl supplemented with protease inhibitors mixture (Complete, EDTA-free, Roche)]. Bacteria were transferred to 2 mL lysing matrix tubes (MP Biomedicals) and mechanically lysed by bead beating in a FastPrep apparatus (45 s, speed 6.5 three cycles). Subsequently, tubes were centrifuged (10 min at 16,000 × *g*, 4°C) to remove cellular debris. To quantify the partition of Zea between bacterial cytosol and culture medium, equal volumes of culture supernatant and bacterial cytosol were analyzed by gradient SDS-PAGE and subjected to immunoblotting via wet transfer onto 0.45 μm nitrocellulose membrane (Millipore). EF-Tu and InlC proteins were used as marker of intracellular and extracellular fractions (Prokop et al., 2017). Detection of overexpressed ZeaFlag in *L. monocytogenes* was performed as described above, except for the protein precipitation from culture medium which was performed as previously described (Archambaud et al., 2005). Briefly, 16% of trichloroacetic acid (TCA) (Sigma) was added to the filtered culture medium and samples were left on ice for 2 h. Precipitated proteins were recovered by centrifugation (20 min, 16,000 × *g*, 4°C). The protein pellets were washed twice with ice-cold acetone and dried at 95°C for 5 min. Proteins were resuspended in NuPage LDS sample buffer and an equal percentage of bacterial cytosol and culture medium were subjected to immunoblotting as above.

Expression and Purification of HisZea

pET28a-HisZea (described above) was used to transform *E. coli* C43 bacteria which were grown at 37°C in Terrific broth (TB) (Thermo Fisher Scientific) supplemented with 50 μg/mL kanamycin. Expression was induced by the addition of IPTG to a final concentration of 1 mM at OD_{600nm} = 0.7 AU. Cultures were incubated overnight, and cells were harvested by centrifugation (5,500 × *g*, 20 min, 4°C). The bacterial pellet was resuspended in Buffer A (50 mM potassium phosphate pH 7.0, 300 mM NaCl, 10% glycerol, 20 mM imidazole, 2 mM beta-mercaptoethanol). All subsequent steps were performed at 4°C. Cell lysis was carried out by passing the samples three times through a pre-cooled microfluidizer operating at 17,000 psi. The soluble fraction was then obtained by centrifugation at 39,000 × *g* for 45 min at 4°C. Subsequently, the supernatant was loaded onto a pre-equilibrated Ni-NTA column (QIAGEN) at 0.5 mL/min with a peristaltic pump at 4°C. The washing and elution steps were performed on an AKTA system using steps of 35% and 100% Buffer B (50 mM potassium phosphate pH 7.0, 300 mM NaCl, 10% glycerol, 300 mM imidazole, 2 mM beta-mercaptoethanol). The fractions containing Zea were pooled, concentrated with an Amicon centrifugal filter unit (10K cut-off, Millipore), and further purified by size-exclusion chromatography on a Hi Load S200 10/300 column (GE Healthcare) pre-equilibrated in Buffer C (50 mM potassium phosphate pH 7.0, 300 mM NaCl, 10% glycerol, 2 mM beta-mercaptoethanol). Peak fractions were pooled, concentrated to 10 mg/mL, and subsequently dialyzed against Buffer D (50 mM potassium phosphate pH 7.0, 100 mM NaCl,

10% glycerol, 2 mM beta-mercaptoethanol). After dialysis, protein concentration was assessed again and the sample was flash-frozen in liquid nitrogen. During purification, the purity and homogeneity of the sample were monitored by SDS-PAGE.

RNA Extraction

Total RNA from *L. monocytogenes* was extracted as previously described (Mellin et al., 2013). Briefly, bacteria grown either to exponential phase (OD_{600nm} = 0.8-1.0 for growth cultures in BHI or OD_{600nm} = 0.4 for growth cultures in MM) or stationary phase (overnight culture: OD_{600nm} = 3.0-3.5 for growth cultures in BHI, or OD_{600nm} = 1.0 for growth cultures in MM) were pelleted by centrifugation (2862 × *g*, 20 min, 4°C). Pellets were resuspended in 1 mL TRIzol Reagent (Ambion), transferred to 2 mL Lysing Matrix tubes and mechanically lysed by bead beating in a FastPrep apparatus (45 s, speed 6.5 followed by an additional 30 s, speed setting 6.5). Subsequently tubes were centrifuged (5 min at 8,000 × *g*, 4°C) in a tabletop centrifuge and lysates were transferred to a 2 mL Eppendorf tube. RNA isolation proceeded according to the manufacturer's instructions. Briefly, 200 μL of chloroform (Sigma) were added to the lysate, shaken and incubated for 10 min at room temperature, followed by centrifugation (15 min at 13,000 × *g*, 4°C). The upper aqueous phase was removed and transferred to a new 1.5 mL Eppendorf tube and RNA was precipitated by the addition of 500 μL isopropanol and incubation at room temperature for 5– 10 min. RNA pellets (10 min at 13,000 × *g*, 4°C) were washed twice with 75% ethanol and resuspended in 50 μL of nuclease-free water (Ambion).

To extract total secreted RNA from MM, *L. monocytogenes* was grown until exponential phase (OD_{600nm} = 0.4). Medium was recovered by centrifugation (2862 × *g*, 20 min, 4°C), filtered (0.22 μm) and concentrated 10 times using an Amicon centrifugal filter unit (3K cut-off). Medium was then brought back to the initial volume by adding nuclease-free water and concentrated again. This desalting process was repeated three times to avoid co-precipitation of salts during the subsequent RNA isolation. RNA was then extracted twice with acid phenol/chloroform, precipitated with ethanol/0.3 M sodium acetate and resuspended in nuclease-free water.

RNA extraction from LoVo cell monolayers in 6-well plates was performed by using TRIzol Reagent. Briefly, cells were washed once with ice-cold PBS and directly lysed in the well by adding 1 mL of TRIzol and gentle pipetting. Samples were vortexed thoroughly for 30 s before the addition of 200 μL chloroform and then incubated 3 min at room temperature. After centrifugation (15 min, 12 000 × *g*, at 4°C), the upper aqueous phase was transferred to a new Eppendorf tube and RNA was precipitated by the addition of an equal volume of isopropanol and incubation at room temperature for 10 min. RNA pellet was washed twice with 70% ethanol and resuspended in 50 μL of nuclease-free water.

In Vitro RNA Transcription

cDNA templates of the *L. monocytogenes* small RNAs fused with a T7 promoter were obtained by PCR amplification from genomic DNA with the appropriate primers. The cDNA quality was verified on a 1% agarose gel and visualized by ethidium bromide staining. cDNA was purified from agarose gel with a Gel extraction kit (QIAGEN) and resuspended in nuclease-free water. Purified cDNA (200 ng) was transcribed *in vitro* by using the MAXIscript T7 *in vitro* transcription kit (Invitrogen) according to the manufacturer's recommendation. The quality of the *in vitro*-transcribed RNA was verified by SYBR Gold (Life Technologies) staining after running on 6% Novex TBE-Urea gel (Thermo Fisher Scientific) or by the Bioanalyser RNA nano kit (Agilent). The p2RZ vector expressing a part of Cherry protein transcript was described elsewhere (Chazal et al., 2018) and linearized by XhoI before performing *in vitro* transcription, as described above. The biotinylated small RNAs were also *in vitro*-transcribed as above, except that 0.35 mM of biotin-16-UTP (Roche) was included in the reaction mixture.

Electrophoretic Mobility Gel Shift Assay

In vitro formation of HisZea - RNA complexes was assessed by electrophoretic mobility gel shift assay (EMSA). For *in vitro* RNA synthesis, 1 μg of cDNA template carrying a T7 promoter was amplified by PCR and *in vitro*-transcribed using the MAXIscript T7 *in vitro* transcription kit according to the manufacturer's instructions. The quality of the *in vitro*-transcribed RNA was verified as described above. RNA was purified and concentrated using "RNA clean & concentrator" (Zymo research) before dephosphorylation and 5′ end labeling as previously described (Chevalier et al., 2009). Labeled RNA was denatured for 1 min at 95°C, chilled on ice (5 min) and renatured by slowly cooling down to 25°C. Upon addition of HisZea (concentrations as indicated in the figure legends) the complex was formed in 20 μL of binding buffer [50 mM Tris pH 8.0, 300 mM NaCl, 10% glycerol, 50 μg/mL fatty acid-free BSA (Roche), supplemented with 1 μg of yeast tRNA (Invitrogen)] for 20 min at room temperature. Unlabeled competitor was added and samples were incubated for an additional 20 min. Samples were mixed with loading buffer (50% glycerol, 0.5% tris-borate EDTA and 0.1% xylene cyanol) before running on native 8% Novex TBE gels (Thermo Fisher Scientific). Signals were detected by autoradiography (at least one-h exposure at −80°C in presence of an intensifying screen).

Biotin Pull-Down Assay

HisZea (2.5 μg) was incubated with 50 μL of equilibrated streptavidin magnetic beads (BioLabs) in 250 μL of binding buffer (150 mM KCl, 25 mM Tris pH 8.0, 5 mM EDTA, 0.5 mM DTT, 0.5% NP40) for 45 min at 4°C with shaking. Beads were recovered by centrifugation (500 × *g*, 5 min, 4°C) and discarded. The HisZeap-containing supernatant was used in the subsequent steps. This preclearing step was performed in order to remove the Zea fraction which aggregated non-specifically onto the beads. Biotinylated RNA (500 nM) was added to the HisZea-containing supernatant and incubated for 30 min, 4°C with shaking. Then, 50 μL of equilibrated streptavidin magnetic beads were added for a further incubation (30 min, 4°C with shaking). The beads were then washed four times in binding buffer and bound HisZea was recovered by addition of NuPage LDS sample buffer.

RNase Protection Assay

Equimolar concentrations of HisZea or GST (1 μM) were mixed with ^{32}P radiolabelled rli143 in 20 μL of binding buffer (50 mM Tris pH 8.0, 300 mM NaCl, 10% glycerol and 50 μg/mL fatty acid-free BSA) and incubated at 25°C for 30 min. Then, 0.0033U of RNaseI (Ambion) were added before incubation for either 1 or 3 min at 37°C. Reactions were stopped by addition of NuPage LDS sample buffer and samples were loaded on 8% Novex TBE-Urea gels (Thermo Fisher Scientific). Signals were detected by autoradiography (at least one-h exposure at −80°C in presence of an intensifying screen).

Immunoprecipitations

To assess the interaction between ZeaFlag and ZeaHA, 25 mL of *L. monocytogenes* overnight cultures expressing either ZeaFlag alone, or both ZeaFlag and ZeaHA were centrifuged (2862 × *g*, 20 min, 4°C) to collect bacteria and culture medium. The recovered medium was filtered by using Millex-GP 0.22 μm filters (Millipore), supplemented with 0.2% of Triton X-100, before adding 20 μL of M2 Flag magnetic beads (Sigma). Samples were shaken for 2 h at 4°C and then washed four times with lysis buffer (20mM Tris pH 8.0, 1 mM $MgCl_2$, 150 mM KCl, supplemented with protease inhibitors mixture). The immunoprecipitated material was finally eluted using 100 μg/mL of 3xFlag peptide (Sigma) according to the manufacturer's instructions. Bacterial pellet was washed twice in ice-cold PBS, resuspended in lysis buffer and lysed in a FastPrep apparatus (45 s, speed 6.5, thrice). The samples were then clarified by centrifugation (14000 × *g*, 10 min, 4°C, twice) and protein concentration determined by Bradford assay. The same percentage of bacterial cytosol compared to the culture medium was used to immunoprecipitate ZeaFlag, under the same condition used for the culture medium. Equal amounts of eluted proteins were subjected to immunoblotting via wet transfer onto a 0.45 μm nitrocellulose membrane.

To assess the interaction between ZeaFlag and Strep-RIG-I, 25 mL of culture medium from *L. monocytogenes wt* and *zea^{Flag}* overnight cultures were recovered by centrifugation (2862 × *g*, 30 min, 4°C) and filtered by using Millex-GP 0.22-μm filters. Filtered culture medium was supplemented with 0.2% Igepal. Then, 25 μL of M2 Flag magnetic beads were added and samples were incubated overnight at 4°C with shaking. Magnetic beads were recovered and washed four times with washing buffer (20 mM MOPS-KOH pH 7.4, 120 mM KCl, 0.2% Igepal, 2 mM beta-mercaptoethanol) and left on ice while preparing the cell lysate. HEK293 cells stably transfected with Strep-RIG-I were lysed in lysis buffer [20 mM MOPS-KOH pH 7.4, 120 mM KCl, 0.2% Igepal, 2 mM beta-mercaptoethanol, supplemented with a protease inhibitors mixture and 12.5 U/μl RNasin (Promega)], sonicated twice for 15 s at 20% amplitude and incubated on ice for 30 min. The cell lysate was cleared by centrifugation (14000 × *g*, 10 min, 4°C) with the supernatant assayed for protein concentration with Bradford assay and used fresh. At least 1 mg of cell lysate was added to the Zea-containing washed Flag magnetic beads (prepared above) and incubated overnight at 4°C with shaking. Beads were washed four times in washing buffer and twice in washing buffer without Igepal. For RNaseA treatment, 100 μg/mL of RNaseA (Roche) in lysis buffer without Igepal were added to the beads (30 min, ice) followed by two further washes in the same buffer. Zea was eluted from the magnetic beads with 3xFlag peptide at 100 μg/mL, according to the manufacturer's recommendations, in a total volume of 50 μL. Samples were then subjected to immunoblotting via wet transfer onto a 0.45-μm nitrocellulose membrane.

For ZeaFlag immunoprecipitation from mammalian cells, LoVo cells in 10-cm^2 dishes were transiently co-transfected with 7 μg of each DNA (ZeaFlag and Strep-RIG-I) using 24 μL of Lipofectamine LTX (Thermo Fisher Scientific). 24 h after transfection, the cells were washed twice with PBS and lysed using 1 mL lysis buffer per dish (20 mM MOPS-KOH pH 7.4, 120 mM KCl, 0.2% Igepal, 2 mM beta-mercaptoethanol, supplemented with protease inhibitors mixture). The lysate was sonicated for 15 s, at 20% amplitude and incubated on ice for 30 min with shaking. The lysate was then clarified (13,000 × *g*, 10 min, 4°C) and assayed for protein concentration (Bradford). 0.5 mg of total lysate was incubated with 15 μL of M2 Flag magnetic beads (overnight, 4°C, shaking). Beads were recovered and washed three times in lysis buffer before treatment with RNase A and elution with the 3xFlag peptide (both performed as above). Samples were then subjected to immunoblotting via wet transfer onto a 0.45-μm nitrocellulose membrane.

For Hfq immunoprecipitation, bacteria were grown in 20 mL of BHI until stationary phase and pelleted (2862 × *g*, 30 min, 4°C). The bacterial pellet was washed twice with ice-cold PBS and mechanically lysed in 1 mL of lysis buffer (20 mM Tris pH 8.0, 1 mM $MgCl_2$, 150 mM KCl, 1 mM DTT, supplemented with protease inhibitors) in 2 mL Lysing Matrix tubes by bead beating in a FastPrep apparatus (45 s, speed 6.5, thrice). Bacterial lysate was clarified by centrifugation (18407 × *g,* 20 min, 4°C) and protein concentration was determined by Bradford assay. The culture medium was filtered by using Millex-GP 0.22-μM filters. 5 μL of anti-Hfq anti-serum were added to an equal percentage of bacterial cytosol and culture medium and incubated overnight at 4°C under shaking condition. Then, 50 μL of protein A Sepharose beads (GE Healtcare) were added for a further h of incubation (4°C, shaking). The immune complexes were collected by centrifugation (500 × *g*, 5 min, 4°C). After three washes with lysis buffer, the bound protein was eluted from the protein A Sepharose beads by boiling (10 min) in 50 μL LDS sample buffer.

For immunoprecipitation of ZeaFlag from nuclear and cytosolic fractions of infected LoVo cells (6 h, MOI 50), 20 μL of pre-equilibrated M2 Flag magnetic beads were added to equal percentage of cytosolic and nuclear fractions. Samples were incubated overnight at 4°C with shaking. After three washes in lysis buffer (20 mM Tris pH 8.0, 150 mM NaCl, 1 mM DTT, 1% Igepal), immune complexes were retrieved by adding 100 mg/mL of 3xFlag peptide. Samples were then subjected to immunoblotting via wet transfer onto a 0.45-μM nitrocellulose membrane.

Immunofluorescence

Immunofluorescence was performed as previously described (David et al., 2018). Briefly, cells were fixed for 10 min in 4% paraformaldehyde/PBS at room temperature, permeabilized for 5 min in 0.1% Triton X-100/PBS and blocked for 10 min in 1% BSA, 10%

goat serum/PBS. Cells were then incubated for 1 h with primary antibody, washed in PBS, incubated for 45 min with secondary antibody/DAPI, washed again as above and mounted in Vectashield.

Cell Fractionations

Fractionation of cultured cell lines was performed as previously described (Pereira et al., 2018). Cell were resuspended in buffer A (20 mM HEPES pH 7, 0.15 mM EDTA, 0.15 mM EGTA, 10 mM KCl). 1% NP40 was added, followed by SR buffer (50 mM HEPES pH 7, 0.25 mM EDTA, 10 mM KCl, 70% (m/v) saccharose). Samples were centrifuged for 500 × *g*, 4°C). The supernatant was isolated as the cytosolic fraction and recentrifuged as before to eliminate cell nuclear debris. The pellet was washed in buffer B (10 mM HEPES pH 8, 0.1 mM EDTA, 100 mM NaCl, 25% (v/v) glycerol) and centrifuged 5 min at 500 × *g*, 4°C. Buffer A, B and SR were supplemented with 0.15 mM spermidine, 0.15 mM spermine, 1 mM DTT and protease inhibitor. The washed pellet was resuspended in sucrose buffer (20 mM Tris pH 7.65, 60 mM NaCl, 15 mM KCl, 0.34 M sucrose, 0.15 mM spermidine, 0.15 mM spermine), followed by the addition of a high-salt buffer (20 mM Tris pH 7.65, 0.2 mM EDTA, 25% glycerol, 900 mM NaCl, 1.5 mM $MgCl_2$) to obtain a final salt concentration of 250 mM. Samples were incubated for 25 min and centrifuged for 10 min at 13,000 × *g*, 4°C. The supernatant was isolated as the nuclear soluble fraction from the pellet which represents chromatin and nuclear insoluble material. The pellet was resuspended in sucrose buffer supplemented with 0.0025 U/ μL) and 1 mM $CaCl_2$ and was incubated at 37°C for 10 min. 4mM EDTA was added and samples were sonicated using the Bioruptor (Diagenode) for 7.5 min (15 s on and 1 min off) and centrifuged for 15 min at 13,000 × *g*, 4°C. The supernatants represent a soluble chromatin fraction.

Live/Dead Bacterial Staining

L. monocytogenes was grown in 20 mL of BHI until stationary phase. Bacteria were pelleted (2862 × *g*, 20 min, room temperature) and then stained with *LIVE/DEAD BacLight* (Molecular Probes), following the manufacturer's recommendation. Bacterial suspension (20 μL) was then deposited on a glass coverslip and immediately imaged by using a Zeiss AxioObserver.Z1 inverted fluorescence microscope equipped with an Evolve EM-CCD camera (Photometrics). Images were acquired with a 100 × N.A. 1.4 oil objective using MetaMorph.

RIP-Seq

50 mL *Δzea+zea*$^{+}$ *L. monocytogenes* stationary phase (OD_{600nm} = 3.5) culture (a zea-deletion strain in which one copy of the zea gene was integrated in the *L. monocytogenes* genome under the control of a constitutive promoter) was centrifuged (2862 × *g*, 20 min, 4°C) to recover the culture medium and the bacterial pellet. 10 mL culture medium were filtered (0.22-μm) and concentrated to 1 mL by using Amicon centrifugal filter unit (3K cut-off). Concentrated medium was then supplemented with 0,05% of Triton X-100, centrifuged again (18407 × *g,* 20 min, 4°C) and left on ice while preparing bacterial cytosolic extracts. The bacterial pellet was washed thrice in ice-cold PBS and lysed by mechanical shaking in a FastPrep apparatus (described above) in 1 mL of lysis buffer (25mM Tris pH 7.4, 150mM KCl, 1mM DTT, 0.05% Triton X-100) supplemented with protease inhibitors mixture. Bacterial cytosol was recovered by centrifugation (two serial centrifugations at 18407 × *g,* 20 min, 4°C) and protein concentration determine by Bradford assay. One-milliliter of bacterial cytosol and concentrated culture medium (corresponding to 50 mL and 10 mL of the bacterial cytosol and culture medium, respectively) were individually loaded on a Superose 6 10/300 GL column pre-equilibrated with lysis buffer without Triton X-100. About 161 fractions of 220 μL were collected, and one out of every seven fractions was concentrated by acetone precipitation and the presence of Zea and EF-Tu analyzed by immunoblotting after wet transfer onto a nitrocellulose membrane. Fractions containing the complexes (A or B) were then pooled and processed for immunoprecipitation assays. Briefly, each sample was incubated overnight at 4°C with shacking, with a mix of 30 μg of anti-Zea antibodies (10 μg of each antibody) or 30 μg of normal rabbit IgG (CellSignaling) . Then, 50 μL of Protein A Sepharose were added for further 2 h to recover immunocomplexes. The beads were washed four times with lysis buffer and treated with Turbo DNase (Ambion) for 10 min at 37°C in 200 μL 1X Turbo DNase buffer. Samples were vortexed for 30 s after the addition of 200 μL of acid phenol (Ambion). Then, 50 μL chloroform (Sigma) were added and samples were centrifuged for 5 min at 10 000 × *g* at 4°C. The aqueous upper phase was recovered and transferred to a new Eppendorf tube. RNA was precipitated by adding the same volume of isopropanol and 0.3 M sodium acetate (Ambion) and 1 μL of glycogen (Invitrogen). Samples were centrifuged (30 min, 10 000 × *g*, 4°C), and RNA was washed once with 70% ethanol before being resuspended in 25 μL of nuclease-free water. RNA was analyzed with the Bioanalyser RNA pico kit (Agilent). Purified RNA was fragmented with the "RNA fragmentation reagents" (Thermofisher), purified by ethanol precipitation and quality-controlled with the Bioanalyser, as described above. Directional RNA-seq libraries were prepared with 30 ng of purified RNA for each sample by using NEBNext Multiplex Small RNA Library Prep Set for Illumina (New England Biolabs) according to the manufacturer's instructions. When required, the RNA samples were spiked-in with a synthetic *in vitro*-transcribed AdML splicing reporter (Allemand et al., 2016) in order to have 30 ng of total RNA. Libraries were sequenced on an Illumina HiSeq 2500 platform (SR100).

RIP-Seq Data Analysis

The *L. monocytogenes* EGD-e genome (NC_003210) and a list of 3160 transcripts (genes, small-RNAs, tRNAs, and rRNAs) were downloaded from the Listeriomics database (Bécavin et al., 2017). After the sequencing of all RIP-Seq samples, the resulting reads were trimmed (AlienTrimmer 0.4.0, default parameters) (Criscuolo and Brisse, 2013). They were mapped on the EGD-e genome using Bowtie2 2.1.0 (very-sensitive parameter) (Langmead and Salzberg, 2012). Mapping files were filtered to keep uniquely mapped reads using SAMtools 0.1.19 (*samtools view -b –q 1* parameters) (Li et al., 2009), and saved to BAM files after indexation. Read Per Million

coverage files were saved in BigWig format using bamCoverage package from deepTools 3.1.3 (Ramírez et al., 2016). The quality of the sequencing and mapping was assessed using FastQC 0.10.1 and MultiQC 0.7 (Ewels et al., 2016). The number of reads per transcript (mRNA, sRNA, tRNA, rRNA) was counted using FeatureCount (1.4.6-p3 default parameters) (Liao et al., 2014). Statistical analysis was performed using SARTools package (Varet et al., 2016) and in-house R scripts (https://github.com/becavin-lab/RIPSeq-Listeria). Data were normalized with the TMM (Robinson et al., 2010) (edgeR package) normalization method. Finally, the $\log_2$(Fold changes) were calculated by subtraction of $\log_2$(TMM) normalized expression values.

Sequencing of Total Secreted *L. monocytogenes* RNA

To extract total secreted RNA from the culture medium, *L. monocytogenes* strains (*wt* and *Δzea+zea*$^+$) were grown to exponential phase (OD_{600nm} = 0.4) in 14 mL of MM under microaerophilic conditions using Oxoid AnaeroGen 2.5L gas packs (Thermo Fisher) at 25°C. Under this condition *zea* appeared slightly upregulated compared to standard growth conditions (i.e 37°C, BHI medium) (Bécavin et al., 2017). A parallel culture (same conditions) was set-up to check the OD and arrest the bacterial growth when the strains reached the same OD. The culture medium was then recovered by centrifugation (2862 × *g*, 20 min, 4°C) and filtered (0.22 μm). The bacterial pellet was stored at −80°C for subsequent RNA extraction. 10 mL of the filtered culture medium were desalted and the RNA was extracted as described above. The quality of the RNA was checked by using the Bioanalyser RNA nano kit. The amount of recovered RNA was similar in all the samples. Total secreted RNA (5 μg) was ribodepleted by using the Ribo Zero rRNA removal kit (Illumina) following the manufacturer's instructions. Ribodepletion was controlled by the Bioanalyser RNA pico kit. Directional RNA-seq libraries were prepared with 100 ng of purified RNA for each sample by using NEBNext Multiplex Small RNA Library Prep Set for Illumina according to the manufacturer's instructions. Libraries were sequenced on an Illumina NextSeq500 platform (SR75).

Sequencing of Secreted *L. monocytogenes* RNA

The RNA-seq datasets were first trimmed to keep only reads longer than 45bp (AlienTrimmer 0.4.0, *-l 45*) (Criscuolo and Brisse, 2013). They were mapped on the EGD-e genome using Bowtie2 2.1.0 (very-sensitive parameter) (Langmead and Salzberg, 2012). Mapping files were filtered to keep uniquely mapped reads using SAMtools 0.1.19 (*samtools view -b –q 1* parameters) (Li et al., 2009), and saved to BAM files after indexation. Read Per Million coverage files were saved in BigWig format using bamCoverage package from deepTools 3.1.3 (Ramírez et al., 2016). The quality of the sequencing and mapping was assessed using FastQC 0.10.1 and MultiQC 0.7(Ewels et al., 2016). The number of reads per transcript (mRNA, sRNA) was counted using HTSeq 0.9.1(*-s no -m union–nonunique all* parameters) (Anders et al., 2015). Differential analysis was performed using SARTools (Varet et al., 2016) and DESeq2 R (Love et al., 2014) packages.

RIP-qPCR

L. monocytogenes bacterial cultures (*Δzea+zea*$^+$ strain) were processed essentially as described for the RIP-seq experiment unless otherwise stated. In summary, *L. monocytogenes* was grown until the stationary phase (OD_{600nm} = 3.5) and, for every sample, 50 mL of bacterial culture were processed as follows. Bacteria were pelleted at 2862 × *g*, 20 min, 4°C and culture supernatant was filtered and processed (5 mL) for total RNA purification (input, 10%), by performing two sequential phenol/chloroform extractions followed by ethanol/sodium acetate precipitation. The RNA pellet was washed once with ethanol 70% and resuspended in 20 μL nuclease-free water. Purified RNA was then treated with Turbo DNase and purified again, as described above. The remaining medium (45 mL, 90% of the initial sample) was processed for Zea immunoprecipitation. Briefly, 20 μg of a mix of Zea antibodies (6.6 μg of each antibody) were coupled to 100 μL of Protein A Dynabeads (Invitrogen) for 2 h in 500 μL of PBS (room temperature, shaking). Beads were then washed twice with PBS and once with lysis buffer (25mM Tris pH 7.4, 150mM KCl, 1mM DTT, 0.05% Triton X-100). Culture medium was supplemented with 0.05% Triton X-100 before the addition of the anti-Zea antibody-coupled beads. Samples were then incubated overnight (4°C, shaking). Beads were washed four times with lysis buffer, treated with Turbo DNase and processed for RNA extraction. Purified RNA was stored at −80°C until use. The bacterial pellet was washed thrice in ice-cold PBS and then mechanically lysed by using FastPrep apparatus in 1 mL of lysis buffer supplemented with protease inhibitors mixture and RNasin (Promega) at 12.5 U/μL. Bacterial lysate was clarified by two sequential centrifugations and the final volume was carefully measured. A volume corresponding to 10% of the total sample (input) was treated with DNase and processed for RNA isolation by phenol/chloroform extraction and ethanol/sodium acetate precipitation. Purified RNA was resuspended in nuclease-free water and stored at −80°C until use. The remaining bacterial cytosol was incubated with an anti-Zea antibody coupled to Protein A as described above (overnight, 4°C, shaking). Beads were washed four times with lysis buffer, treated with Turbo DNase and processed for RNA extraction. For qPCR analysis, 100 ng of purified RNA were subjected to reverse transcription in 20 μL final volume using the Reverse Transcription Kit (QIAGEN) according to the manufacturer's instructions. Reactions were then diluted by adding 180 μL of nuclease-free water. qPCR was assayed in 10 μL reactions with Brillant III Ultra Fast SYBR-Green qPCR Master Mix (Agilent). Reactions were carried out in a Stratagene MX3005p system with the following thermal profile: 5 min at 95°C, 37 cycles of 10 s at 95°C and 12 s at 60°C. Results were analyzed with an MxPro software, as described earlier (Batsché et al., 2006).

Quantitative Real-Time PCRs

For qPCR of *L. monocytogenes* secreted RNA (phage, lma-monocin and rli143 RNAs), bacterial strains were grown in MM until exponential phase (OD_{600nm} = 0.4). *L. monocytogenes wt*, *Δzea* and *Δzea+zea*$^+$ strains were used for the phage and lma-monocin

quantification; *L. monocytogenes wt*, *Δzea*, *zea*$^+$ and *lmo2595*$^+$ *L. monocytogenes* strains were used for the quantification of rli143; *L. innocua wt*, *zea-pAD* and *lmo2595-pAD* strains were employed for the quantification of rli143. The bacterial OD was measured, and the cultures were recovered when OD was equal for all the strains. MM was collected by centrifugation, filtered and processed for RNA extraction (as described above). Purified RNA (5-10 μg) was subjected to DNase treatment using the DNase treatment and removal kit (Ambion). Treated RNA (500 ng) was mixed with an equal amount of CleanCap^{TM} OVA mRNA (TriLink) which serves as an internal control for normalization, and processed for reversed transcription and qPCR, as above. Gene expression levels were normalized to the OVA mRNA, and the fold change was calculated using the ΔΔCT method.

For qPCR of *Listeria* genes from total (intracellular) *L. monocytogenes* RNA, the RNA was extracted, as described in the RNA extraction section and treated, as described above, except that the OVA mRNA was not included in the reverse transcription reaction. Gene expression levels were normalized to the *rpob* gene, and the fold change was calculated using the ΔΔCT method.

For qPCR of Hfq-associated RNAs, RNA was extracted from immunoprecipitated Hfq, by using the protocol described for the RIP-seq of Zea. DNase-treated RNA (120 ng) was subjected to reverse transcription. Gene expression levels were normalized to the input fractions, and the fold change was calculated using the ΔΔCT method.

For qPCR of IFNβ, IFNγ and IL-8, mammalian RNA was extracted, as described in the RNA extraction section. Purified RNA (5-10 μg) was subjected to DNase treatment, and 1 μg processed for reverse transcription, as described above. Gene expression levels were normalized to the actin mRNA and to the uninfected samples, and the fold change was calculated using the ΔΔCT method.

Purification of RLRs and RNA Extraction

Four 15-cm^2 tissue culture dishes per cell line were pretreated with 0.1 mg/mL poly-L-Lysine-hydrobromide (Sigma), rinsed with distilled water and dried for 1 h before plating the cells. Cells (30-40x10^6) were plated per dish in 20 mL of DMEM medium for 24 h before infection. Overnight *L. monocytogenes* EGD-e cultures in BHI were diluted 1/20 in fresh BHI the day of infection and grown up to OD_{600nm} = 1. Each plate was infected with an MOI of 50 for 1 h before replacing the media with complete DMEM containing 10 μg/mL of gentamicin to kill extracellular bacteria. After an additional 3 h (in total 4 h of infection), plates were rinsed twice with ice-cold PBS, crosslinked at 400 mJ/cm^2 in 10 mL of ice-cold PBS/plate and cells were then scraped, pelleted and resuspended in 8 mL of MOPS lysis buffer (20 mM MOPS-KOH pH 7.4, 120 mM KCl, 0.5% Igepal, 2 mM beta-mercaptoethanol, supplemented with protease inhibitors mixture and RNasin at 0.2 U/μl and protease inhibitors mixture (Roche). Cell lysates were incubated on ice for 20 min with gentle mixing every 5 min and then clarified by centrifugation at 16000 × *g* for 15 min at 4°C. Streptactin Sepharose beads (GE Healthcare, 100 μL/dish) were washed in MOPS washing buffer (20 mM MOPS-KOH pH 7.4, 120 mM KCl, 2mM beta-mercaptoethanol, supplemented with RNasin 0,2 U/μL and protease inhibitors mixture and finally resuspended in 1 mL of MOPS lysis buffer per initial culture dish. Clarified cell lysate was incubated with Streptactin beads for 2 h at 4°C. The beads were washed three times with MOPS washing buffer and centrifuged at 1600 × *g*, 5 min at 4°C. Strep-tagged proteins were then eluted twice for 15 min at 4°C in 250 μL/dish of 1X elution buffer (IBA, Biotin Elution Buffer 10X). Each sample was treated with proteinase K (Roche) in v/v of 2X proteinase K buffer (200 mM Tris pH 8, 100 mM NaCl, 20 mM EDTA, 4M urea) for 20 min at 4°C that has been preincubated 20 min at 37°C to remove RNase contamination. RNA purification was performed using TRI Reagent LS (Sigma). RNA was dissolved in 50 μL of DNase-free and RNase-free ultrapure water. Extracted RNAs were analyzed using Nanovue (GE Healthcare) and Bioanalyser RNA nano kit (Agilent) before being processed for next-generatio sequencing (HiSeq 2500, SR50).

Data Analysis of RLR-Associated RNAs

Due to the high number of eukaryotic RNAs in the datasets and presence of insertions and deletions, the reads were trimmed (AlienTrimmer 0.4.0) (Criscuolo and Brisse, 2013), and mapped using GSNAP (v2018-07-04) (Wu et al., 2016), a special mapping software allowing variability in reads sequence. Mapping files were filtered to keep uniquely mapped reads using SAMtools 0.1.19 (*samtools view -b –q 1* parameters) (Li et al., 2009), and saved to BAM files after indexation. Read Per Million coverage files were saved in BigWig format using bamCoverage package from deepTools 3.1.3 (Ramírez et al., 2016). The quality of the sequencing and mapping was assessed using FastQC 0.10.1 and MultiQC 0.7 (Ewels et al., 2016). The number of reads per transcript (mRNA, sRNA, tRNA, rRNA) was counted using HTSeq 0.9.1(*-s no -m union–nonunique all* parameters) (Anders et al., 2015). Differential analysis was performed using SARTools (Varet et al., 2016) and EdgeR packages (Robinson et al., 2010) (https://github.com/becavin-lab/RIPSeq-Listeria).

Transfection of Zea-Interacting RNAs

The ISRE reporter cells (STING-37 cell line) (Lucas-Hourani et al., 2013) were seeded in 24-well plates and 2 h later transfected with 100 ng of *in vitro*-transcribed rli143, rli18, rli92 and 250 nucleotides-long fragments of mCherry RNAs (Chazal et al., 2018) using Lipofectamine 2000 (Thermofisher Scientific). 100 ng of high molecular weight (HMW, tlrl-pic, Invivogen) and low molecular weight Poly(I:C) (LMW, tlrl-picw, Invivogen), and 100 ng of short 5′3P RNA (produced as previously described (Lucas-Hourani et al., 2013)) were used as positive controls. Cells were lysed 24 h post-transfection with 200 μL Passive Lysis buffer (Promega). The Firefly luciferase activity was measured using the Bright-Glo Luciferase Assay System (Promega) following the manufacturer's recommendation.

Mice Infections

L. monocytogenes was thawed from glycerol stocks stored at −80°C and diluted in phosphate-buffered saline (PBS) before injection. A sublethal dose (10^4 *L. monocytogenes*) was injected into the lateral vein of the tail of each mouse. The number of bacteria in the inoculum was confirmed by plating serial dilutions of the bacterial suspension onto BHI agar plates. For determination of bacterial loads, livers and spleens were recovered and disrupted in PBS at the indicated time points post-infection. Serial dilutions of organ homogenates were plated onto BHI agar plates, and colony forming units (CFUs) were counted after growth at 37°C for 48 h.

QUANTIFICATION AND STATISTICAL ANALYSIS

All data are expressed as mean and standard error of the mean. Student's t test or ANOVA were used for statistical analysis. Differences in means were considered statistically significant at $p < 0.05$. Sample number (n) indicates the number of independent biological samples in each experiment, for each set of experiments this information is provided in the figure legends.

DATA AND CODE AVAILABILITY

The accession number for RIP-Seq, RNA-seq and RLRs purification and sequencing data reported in this paper is [Array Express at EMBL-EBI]: [E-MTAB-7665]. All scripts used for the analysis have been deposited on the Institut Pasteur GitLab: https://github.com/becavin-lab/RIPSeq-Listeria.

Cell Host & Microbe, Volume *26*

Supplemental Information

An RNA-Binding Protein

Secreted by a Bacterial Pathogen

Modulates RIG-I Signaling

Alessandro Pagliuso, To Nam Tham, Eric Allemand, Stevens Robertin, Bruno Dupuy, Quentin Bertrand, Christophe Bécavin, Mikael Koutero, Valérie Najburg, Marie-Anne Nahori, Frédéric Tangy, Fabrizia Stavru, Sergey Bessonov, Andréa Dessen, Christian Muchardt, Alice Lebreton, Anastassia V. Komarova, and Pascale Cossart

SUPPLEMENTAL INFORMATION

Figure S1. Protein sequence alignment of Zea orthologues, related to Figure 1

Multiple alignment of Zea orthologues. Conserved residues are highlighted with colors. The signal peptides at the N-terminus are highlighted in yellow.

Figure S2. RIP-Seq protocol and COG analysis of Zea-interacting RNAs, related to Figure 2

(**A**) Scheme representing the RIP-Seq protocol (see Methods). (**B**) COG functional classes assigned to Zea-bound. Fisher's exact right rank test was run and q-values [$-\log_{10}$(p-value)] were calculated to detect over-represented COG categories.

Figure S3. Extracellular Zea-bound RNAs do not derive from bacterial lysis, related to Figure 1 and Figure 2

(**A**) Confocal microscopy image of *L. monocytogenes* viability by using live/dead staining assay. Live bacteria are stained with SYTO9 (green in the merged channel) and dead bacteria are stained with propidium iodide (PI, red in the merged channel). (**B**) Representative immunoprecipitation (IP) with an anti-Hfq antibody of *L. monocytogenes* bacterial cytosolic extract and culture medium. Hfq runs as an hexamer (60kD). Asterisks show the positions of the IgG heavy. (**C**) RIP-qPCR (n=2) for *LhrA* on RNA isolated from Hfq and control (IgG) immunoprecipitations in the bacterial cytosol and culture medium. The enrichment of *LhrA* was calculated after normalization to the input fraction.; statistical significance determined by two tailed *t*-test between the IgG IP and Hfq IP; †, not detected.

Figure S4. Phage A118, lma-operon, *rli143* and *zea* expression, related to Figure 4

qRT-PCR analysis (n=3) on total RNA extracted from the indicated *L. monocytogenes* strains grown to the exponential phase in MM. Data were normalized to *16S* rRNA level. (**A**) Quantification of selected phage and control genes; values reprents means ± SEM, n=3. (**B**) Quantification of *zea* expression in MM. (**C**) Quantification of selected genes from the lma-monocin locus. Quantification

of (**D**) *rli143* in *L. monocytogenes* and (**E**) *rli143* in *L. innocua*. Data were normalized to *16S* rRNA and level in the *wt* strain. Statistical significance determined by either unpaired ANOVA with multiple testing against *wt* (for A, C and D) or two tailed *t*-test (for B).

Figure S5. Zea protects *rli143* from RNase degradation and regulates the extracellular amount of a subset of *L. monocytogenes* RNA, related to Figure 4

(**A**) *In vitro*-transcribed radiolabeled *rli143* was incubated with either HisZea or GST. RNase I was subsequently added and samples were incubated at 37 ˚C for the indicated time. (**B**) Differential analysis of RNA-Seq quantification of secreted RNAs from *L. monocytogenes wt* and *Δzea+zea+* strains (see methods). (**C**) Overlap between the RIP-Seq and the RNA-Seq datasets. A Fisher's exact right rank test shows that the ratio of proportion of RNAs differently expressed and enriched in the RIP-seq is greater than 1 (p = 7.18x10^{-5}). The list highlights the genes found in both datasets. (**D**) qRT-PCR analysis on total intracellular RNA extracted from *L. monocytogenes wt* and *Δzea+zea+* for the indicated phage genes. Data were normalized to *16S* rRNA. Statistical significance determined by two tailed *t*-test.

Figure S6. RIG-I binds the *L. monocytogenes* A118 phage RNA during infection, related to Figure 6

Interaction circo plots of *L. monocytogenes* RNAs specifically bound to RIG-I, MDA5 and LGP2 during *L. monocytogenes* infection. A line from the schematized RIG-I to a genome locus indicates a preferential binding to a transcript compared to the negative control mCherry. The asterisks highlight the transcripts that are also enriched in the Zea RIPseq dataset.

Figure S7. Zea localizes both to the host cell cytoplasm and nucleus, related to Figure 6

(A) IP analysis with an anti-Flag antibody of cytosolic and nuclear fractions from LoVo cells infected with *wt* and *zea$_{Flag}$* bacteria. (**B**) Confocal images of LoVo cells transfected with Flag-tagged Zea,

fixed and processed for immunofluorescence by using an anti-Flag antibody (red); nuclei are stained with DAPI. Scale bar, 10 μm.

Figure S1

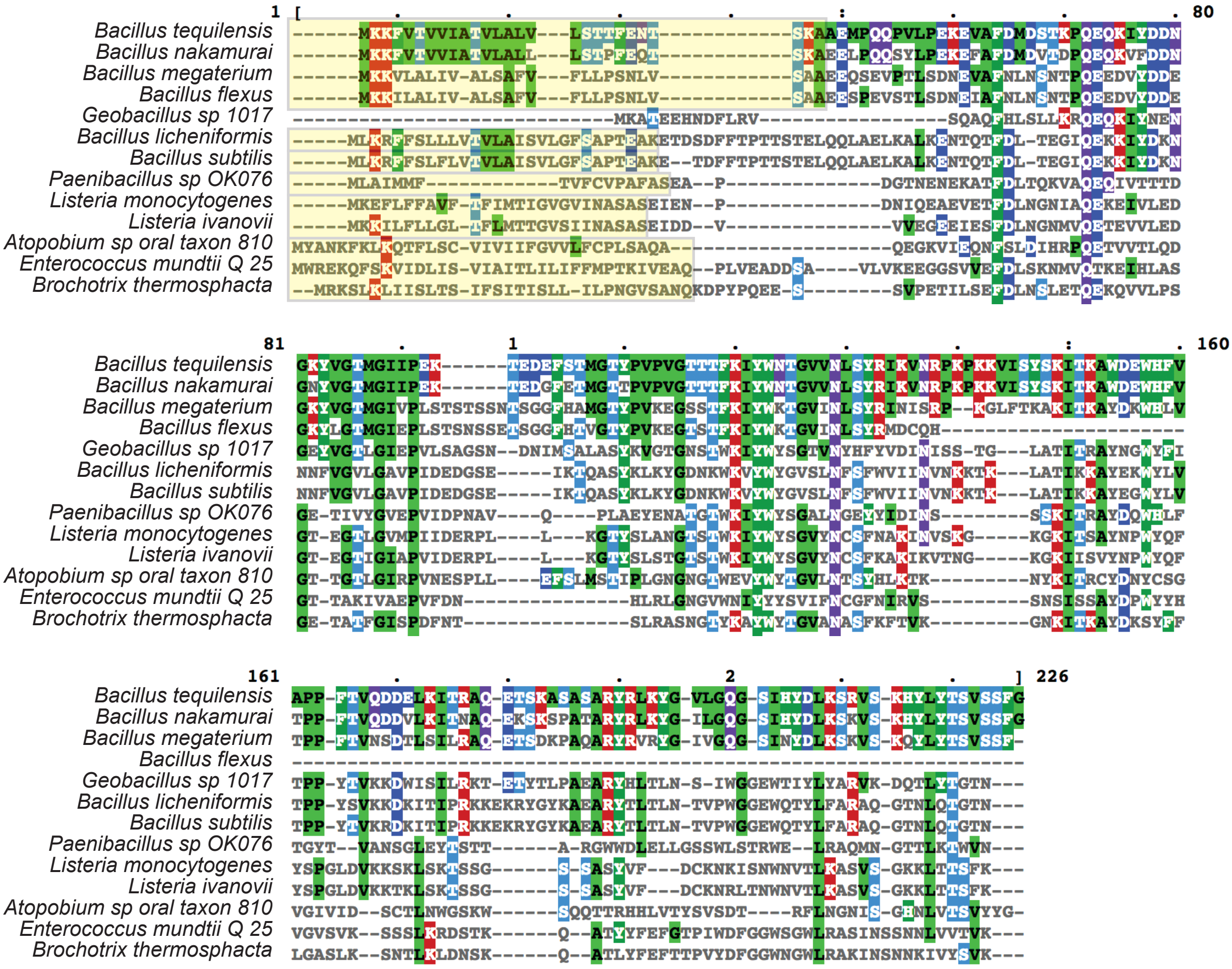
Bacillus tequilensis
Bacillus nakamurai
Bacillus megaterium
Bacillus flexus
Geobacillus sp 1017
Bacillus licheniformis
Bacillus subtilis
Paenibacillus sp OK076
Listeria monocytogenes
Listeria ivanovii
Atopobium sp oral taxon 810
Enterococcus mundtii Q 25
Brochotrix thermosphacta

Figure S2

A

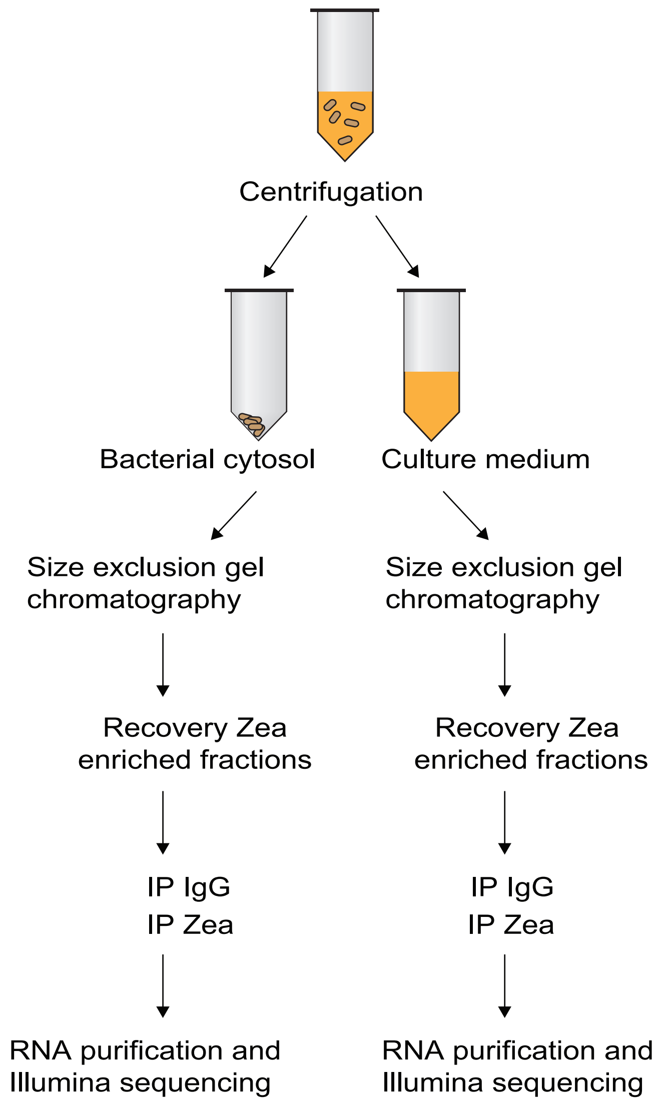
Centrifugation
Bacterial cytosol
Culture medium
Size exclusion gel chromatography
Size exclusion gel chromatography
Recovery Zea enriched fractions
Recovery Zea enriched fractions
IP IgG
IP Zea
IP IgG
IP Zea
RNA purification and Illumina sequencing
RNA purification and Illumina sequencing

B

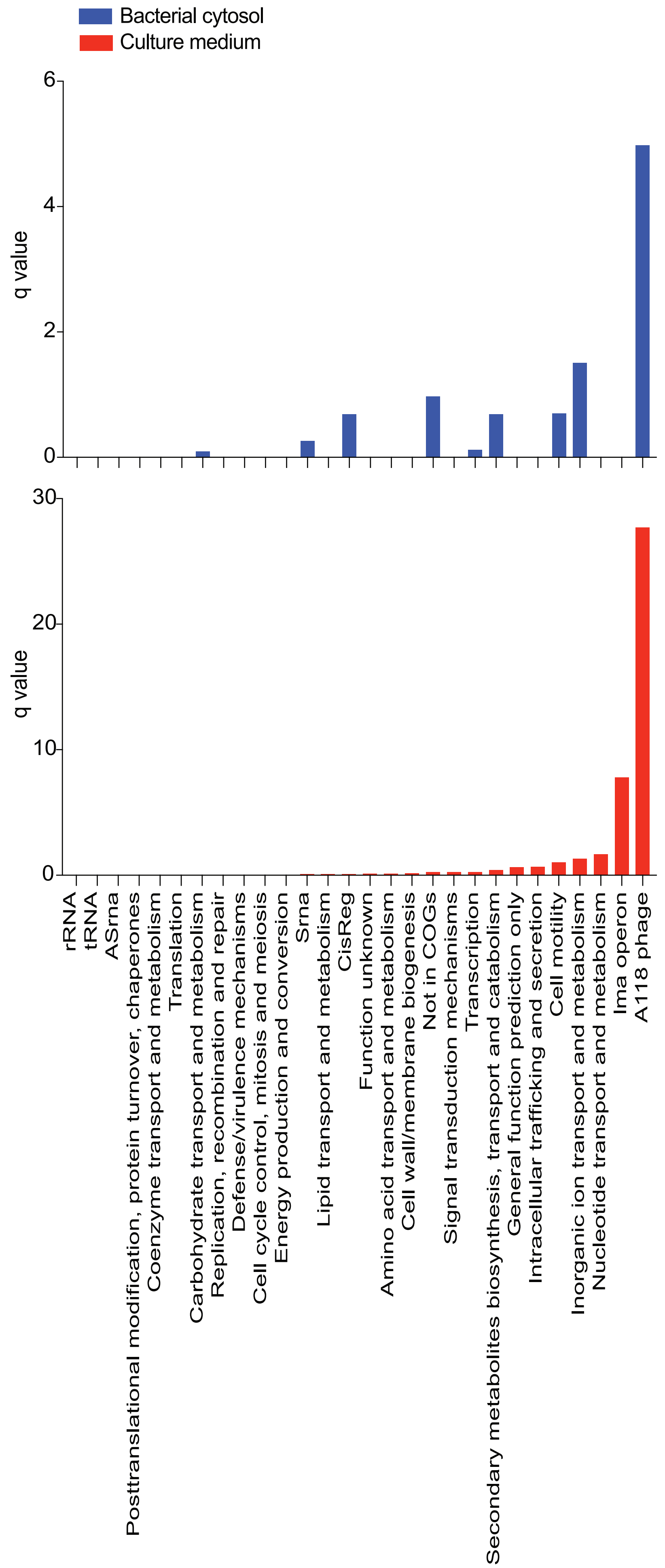
Bacterial cytosol
Culture medium
q value
6
4
2
0
q value
30
20
10
0
rRNA
tRNA
ASrna
Posttranslational modification, protein turnover, chaperones
Coenzyme transport and metabolism
Translation
Carbohydrate transport and metabolism
Replication, recombination and repair
Defense/virulence mechanisms
Cell cycle control, mitosis and meiosis
Energy production and conversion
Srna
Lipid transport and metabolism
CisReg
Function unknown
Amino acid transport and metabolism
Cell wall/membrane biogenesis
Not in COGs
Signal transduction mechanisms
Transcription
Secondary metabolites biosynthesis, transport and catabolism
General function prediction only
Intracellular trafficking and secretion
Cell motility
Inorganic ion transport and metabolism
Nucleotide transport and metabolism
lma operon
A118 phage

Figure S3

A

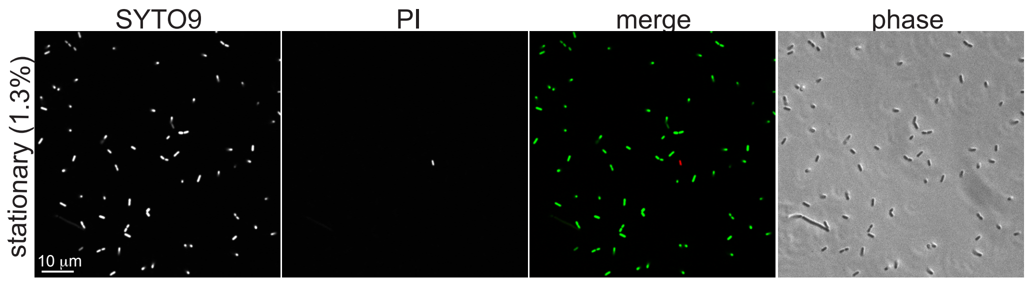

B

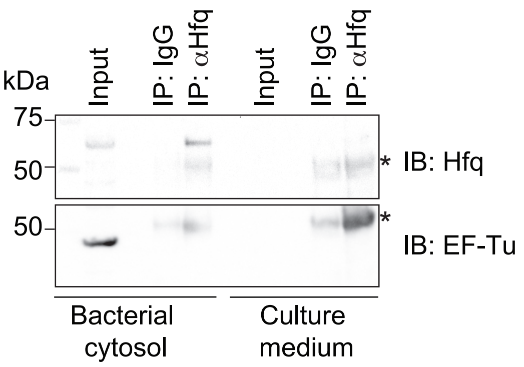

C

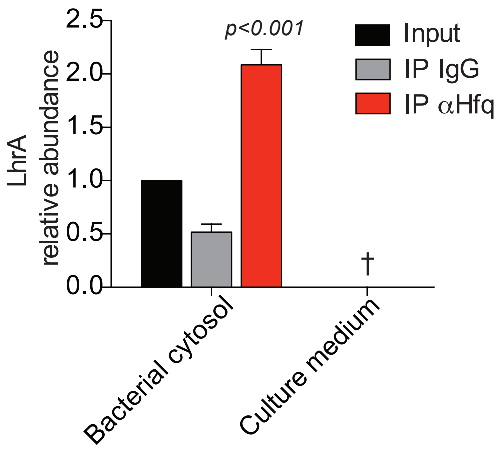

Figure S4

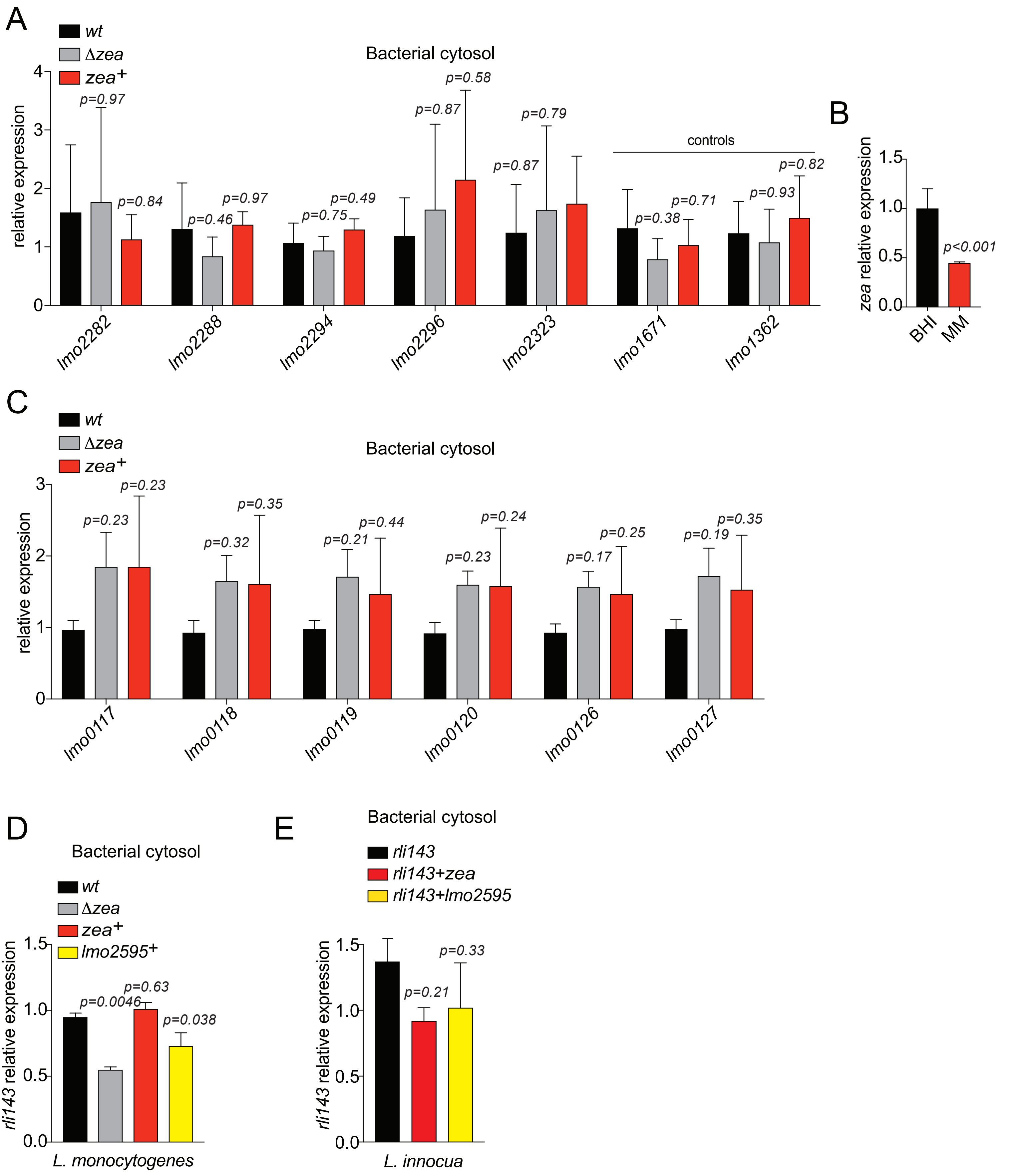
A
wt
Δzea
zea+
Bacterial cytosol
relative expression
p=0.97
p=0.84
p=0.46
p=0.97
p=0.75
p=0.49
p=0.87
p=0.58
p=0.87
p=0.79
controls
p=0.38
p=0.71
p=0.93
p=0.82
lmo2282
lmo2288
lmo2294
lmo2296
lmo2323
lmo1671
lmo1362
B
zea relative expression
p<0.001
BHI
MM
C
wt
Δzea
zea+
Bacterial cytosol
relative expression
p=0.23
p=0.23
p=0.32
p=0.35
p=0.21
p=0.44
p=0.23
p=0.24
p=0.17
p=0.25
p=0.19
p=0.35
lmo0117
lmo0118
lmo0119
lmo0120
lmo0126
lmo0127
D
Bacterial cytosol
wt
Δzea
zea+
lmo2595+
rli143 relative expression
p=0.0046
p=0.63
p=0.038
L. monocytogenes
E
Bacterial cytosol
rli143
rli143+zea
rli143+lmo2595
rli143 relative expression
p=0.21
p=0.33
L. innocua

Figure S5

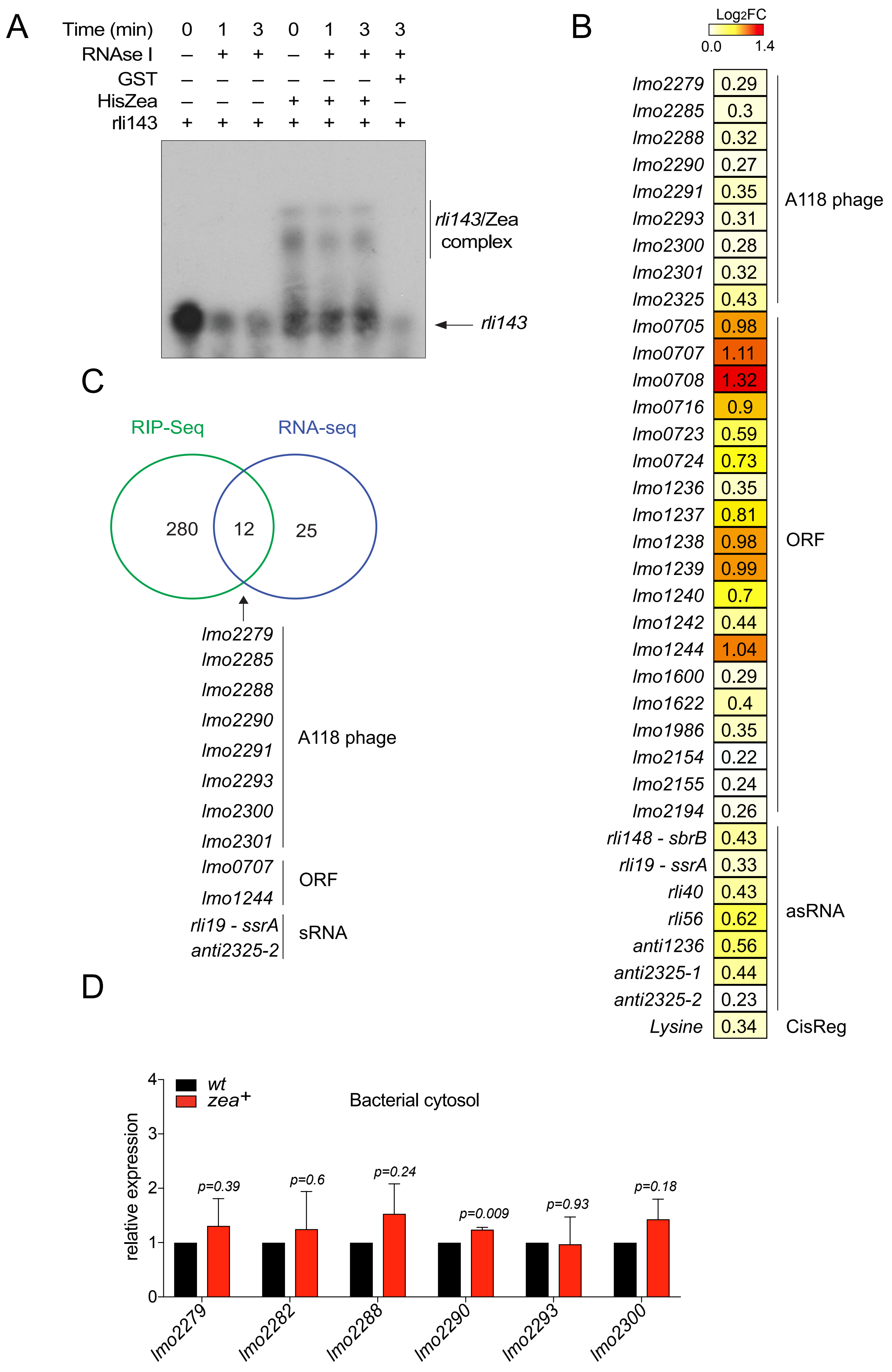
A
Time (min) 0 1 3 0 1 3 3
RNAse I – + + – + + +
GST – – – – – – +
HisZea – – – + + + –
rli143 + + + + + + +
rli143/Zea complex
rli143
B
Log2FC
0.0 1.4
lmo2279 0.29
lmo2285 0.3
lmo2288 0.32
lmo2290 0.27
lmo2291 0.35
lmo2293 0.31
lmo2300 0.28
lmo2301 0.32
lmo2325 0.43
A118 phage
lmo0705 0.98
lmo0707 1.11
lmo0708 1.32
lmo0716 0.9
lmo0723 0.59
lmo0724 0.73
lmo1236 0.35
lmo1237 0.81
lmo1238 0.98
lmo1239 0.99
lmo1240 0.7
lmo1242 0.44
lmo1244 1.04
lmo1600 0.29
lmo1622 0.4
lmo1986 0.35
lmo2154 0.22
lmo2155 0.24
lmo2194 0.26
ORF
rli148 - sbrB 0.43
rli19 - ssrA 0.33
rli40 0.43
rli56 0.62
anti1236 0.56
anti2325-1 0.44
anti2325-2 0.23
asRNA
Lysine 0.34
CisReg
C
RIP-Seq
RNA-seq
280
12
25
lmo2279
lmo2285
lmo2288
lmo2290
lmo2291
lmo2293
lmo2300
lmo2301
A118 phage
lmo0707
lmo1244
ORF
rli19 - ssrA
anti2325-2
sRNA
D
wt
zea+
Bacterial cytosol
relative expression
0
1
2
3
4
p=0.39
p=0.6
p=0.24
p=0.009
p=0.93
p=0.18
lmo2279
lmo2282
lmo2288
lmo2290
lmo2293
lmo2300

Figure S6

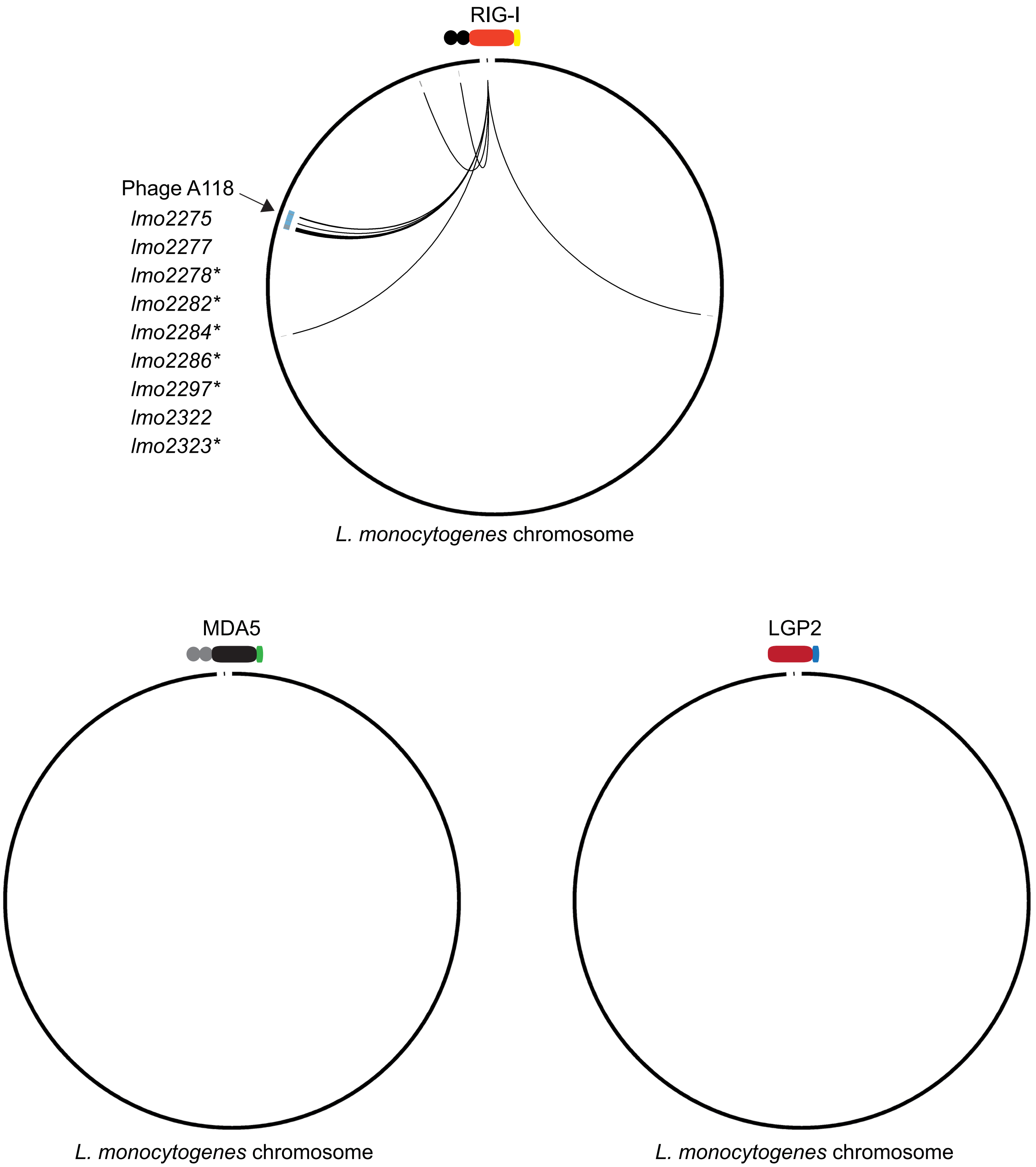
RIG-I
Phage A118
lmo2275
lmo2277
lmo2278*
lmo2282*
lmo2284*
lmo2286*
lmo2297*
lmo2322
lmo2323*
L. monocytogenes chromosome
MDA5
L. monocytogenes chromosome
LGP2
L. monocytogenes chromosome

Figure S7

A

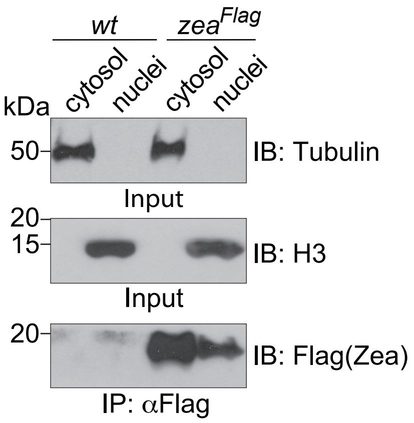
wt
zeaFlag
cytosol
nuclei
cytosol
nuclei
kDa
50
IB: Tubulin
Input
20
15
IB: H3
Input
20
IB: Flag(Zea)
IP: αFlag

B

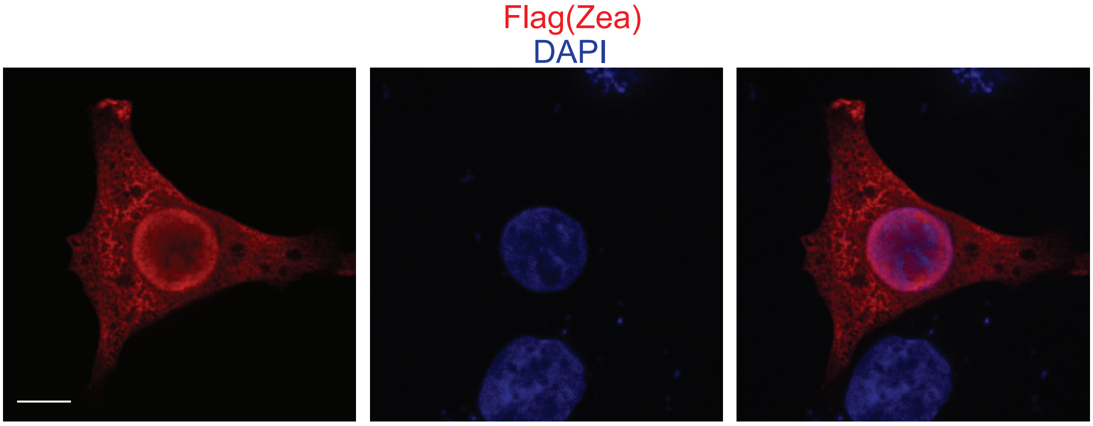
Flag(Zea)
DAPI

Table S1. Identification of Zea-binding RNAs, related to Figure 2

BACTERIAL CYTOSOL	CULTURE MEDIUM
ORF: 89% (Phage 27.7%)	ORF: 94.5% (Phage 14.4%; Lma-monocin locus 3%)
ncRNAs: 11%	ncRNA: 5.5%

Table S2. List of oligonucleotides used in this study, related to STAR Methods

Lmo2282 qPCR rv CCCATCTTTTCTTGTCTTCTTTCCAAATACTTC
Lmo2288 qPCR fw GACAACGTTCTAAGAGTGATTGATTTAACGG
Lmo2288 qPCR rv GCGAGGTTAGCTCTGAACACATCAGAC
lmo2290 qPCR fw GCGTTTGGTTGCCGCATCGCTTATGATG
lmo2290 qPCR rv CCACTGGCAATACAGAAGCATTAATAGTAAATG
lmo2294 qPCR fw GAAAGTAGCAAAACCGATAACAAATGC
lmo2294 qPCR rv CCCGCGATTTGATTTGATTTTGAGAAATCAACAC
lmo2296 qPCR fw CACAGCGGGCGGATTACTTGTTAATATGC
lmo2296 qPCRrv GCATTCAAAGGGTCGTCTCCCGAGATG
lmo2323 qPCR fw GCCGCGATGCAAGTTTTAGCAGAAACTC
lmo2323 qPCR rv CGCGGTTCGTTGTAACTATCAATTAGC
rli18 qPCR fw GATGATATAGCAGGAGATTGCTATG
rli18 qPCR rv GCGTGTTTTACATCCAGTATGGATAG
lmo2615 qPCR fw AACCGTGTTGCTAAAGTAGTTAAAGGTGG
lmo2615 qPCR rv GCATCCTCAACAGCTTTACGGATAGC
lmo1671 qPCR fw TGGTTGCTCAGACGAGAGCGACACAAAAG
lmo1671 qPCR rv CAGAACCAGGCGGATAAATGCTGTG
lmo1362 qPCR fw GGTAGTGCCTCACTTGAAGATTCTCTCG
lmo1362 qPCR rv CACCATCCGCTTCAAAAGGAATTTCTTCTC
Zea qPCR fw GGGAATATAGCTCAAGAGAAAGAAATTG
Zea qPCR rv ACCCTTACTAACATTAATCTTAGCATTAAACGA
rli143 qPCR fw AGTAGAAAAGACCAATGTGGGTTCT
rli143 qPCR rv CAGTTTTTCATCTATAGGTTAATTGCATTAC
lmo0117 qPCR fw CCTACAACGATGATCGAGATGATTG
lmo0117 qPCR rv GCGTATCTATCTTTTCGACTCCC
lmo0118 qPCR fw GCCAACAAGGTCTAACTGTAAAC
lmo0118 qPCR rv CGAAATATTTCGCCAATTCTTTGTGAG
lmo0119 qPCR fw GGAATTAACATCAGGGATTAGTTTAGTTGC
lmo0119 qPCR rv CAGGTACTAAGCCTTTTTCTTCCAC
lmo0120 qPCR fw GAAATGAACCATGATTTTGATTTGGA
lmo0120 qPCR rv CGAAGCATAGTAGATTCAAAAATCACC
lmo126 qPCR fw CGGGTTGACAGAATATGTGTCAG
lmo126 qPCR rv CTCATAAAGCCATCCGTAGTGTATG
lmo127 qPCR fw TTGTGTCGGTTTCAGAGGAAGAG
lmo127 qPCR rv CACCGTTCCTTCACCTATCGTATC
LhrA qPCR fw GGGGATAAGACCCTATCATGGTTATAATGAC
LhrA qPCR rv GACGCTCGAAATAAACATTTCCAGCG
IFNβ qPCR fw CAGCAATTTTCAGTGTCAGAAGC
IFNβ qPCR rv TCATCCTGTCCTTGAGGCAGT
IFNγ qPCR fw GCATCGTTTTGGGTTCTCTTG
IFNγ qPCR rv AGTTCCATTATCCGCTACATCTG
IL8 qPCR fw CAAGAGCCAGGAAGAAACCA
IL8 qPCR rv AGCACTCCTTGGCAAAACTG
rli143 T7 fw GAATTTAATACGACTCACTATAGGAGTAGAAAAGACCAATGTGGGTTC
rli143 T7 rv ACAGTTTTTCATCTATAGGTTAATTGC
rli92 T7 fw GAATTTAATACGACTCACTATAGGGTGTAAAATGCAGCTTGAGTAAAATGAATGAG
rli92 T7 rv GTATAAAACAAACGTGACGTAAAACCAAATC
